# Exploiting individual U–Pb zircon ages and Ti-in-zircon crystallization temperature data to identify high zircon-production events in the Xolapa terrane

**DOI:** 10.1016/j.dib.2019.103933

**Published:** 2019-05-02

**Authors:** T.A. Peña-Alonso, G.P. Villalobos-Escobar, R.M. Molina-Garza

**Affiliations:** a Facultad de Ingeniería, Universidad Autónoma de Tamaulipas, Centro Universitario Tampico-Madero, Tampico, Tamaulipas 89339, Mexico; b Centro de Geociencias, Universidad Nacional Autónoma de México, Blvd. Juriquilla No. 3001, Juriquilla, Querétaro 76230, Mexico

## Abstract

In this article we present a compilation of U–Pb zircon ages of the whole Xolapa terrane in coastal southern Mexico (dataset 1) as a curved line, obtained from plotting individual zircon grains versus its corresponding age. We identified five low-slope segments of the curved line, each one assigned to a high zircon-production (or preservation) event (HZE). Crystallization temperatures (CT) from Ti-in-zircon geothermometer data on Xolapa rocks were estimated separately from individual zircon grains (dataset 2), in order to compare CT ranges corresponding to each HZE identified. Datasets 1 and 2 are discussed for tectonic implications in the research article “The opening and closure of the Jurassic-Cretaceous Xolapa basin, southern Mexico” Peña-Alonso et al., 2017.

**Table:** Specifications table

Subject area	*Geology*
More specific subject area	*Tectonics*
Type of data	*1 Table, 3 Figures, 1 Text file (*Appendix A*), 2 Excel files (*Appendixes B and C*)*.
How data was acquired	*U–Pb age data was acquired from research and technical publications (*Appendixes A and B*). Ti zircon content was acquired from data repositories and an unpublished thesis (*Appendix C*)*.
Data format	*Raw, filtered, processed.*
Experimental factors	*U–Pb zircon ages and Ti zircon contents was collected on distinct datasets.*
Experimental features	*U–Pb age data (dataset 1) vs individual zircon grains is plotted in chronological order to identify low-slope segments of a curved line. Crystallization temperatures (dataset 2) are calculated from the modified Ti-in-zircon geothermometer of**[2]*. *Crystallization temperature ranges are assigned to each low-slope segment of the curved line and described*.
Data source location	*The Xolapa terrane, which covers the states of Guerrero and Oaxaca along the Sierra Madre del Sur, Mexico.*
Data accessibility	*Data are presented in this article.*
Related research article	*[1]**Peña-Alonso, T. A., Molina-Garza, R. S., Villalobos-Escobar, G.P., Estrada-Carmona, J., Levresse, G., & Solari, L. (2018). The opening and closure of the Jurassic-Cretaceous Xolapa basin, southern Mexico. Journal of South American Earth Sciences, 88, 599–620.*

**Table:** 

**Value of the data** • Dataset 1 (2306 U–Pb ages of individual zircon grains from 53 samples collected across the entire Xolapa terrane in southern Mexico) and Dataset 2 (crystallization temperatures from 15 meta-igneous and meta-sedimentary Xolapa rocks, calculated from the Ti-in-zircon geothermometer of [2]) are available as quantitative data to address other research questions relative to crustal accretion. • Individual zircon grains plotted versus their age allow the identification of high zircon-production (or preservation) events (HZE) that can contribute to deduce the accretion and tectonic evolution of the Pacific margin in Mexico. • The procedure performed with the data presented may be used with sets of similar U–Pb ages and Ti-in-zircon geothermometer data to identify HZE from different regions or terranes, and to estimate their corresponding temperature conditions.

## 1 Data

Dataset 1 consists of the age of individual zircon grains from samples obtained in the literature during the last 15 years (Appendixes A and B). Zircon ages are obtained by the Laser Ablation Inductively Coupled Plasma Mass Spectrometry (LA-ICPMS) method using the U–Pb geochronometer.

**Fig. 1:**
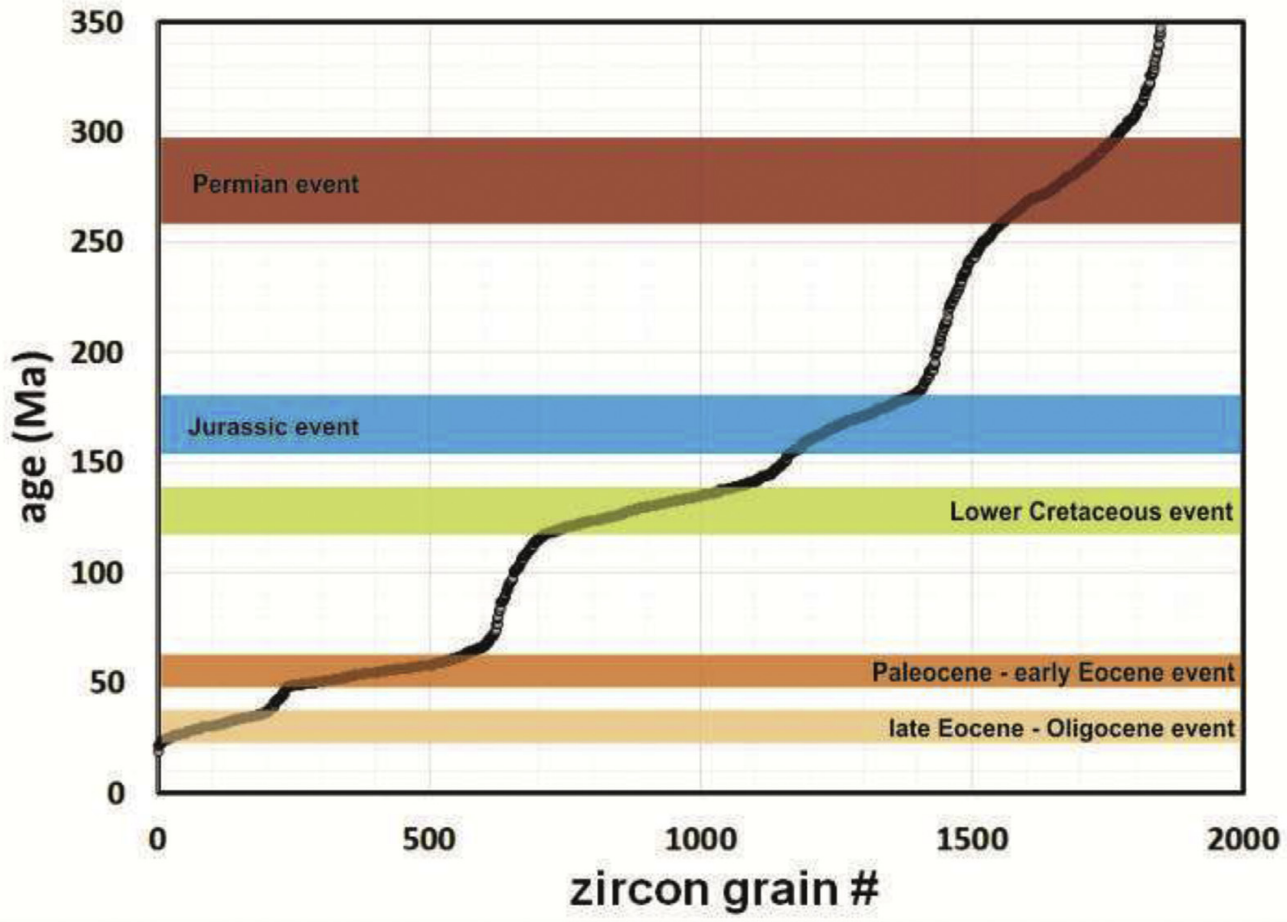
Individual zircon grains versus their corresponding U–Pb zircon ages. Each age datum is represented as a small circle. All plotted points delineate a positive-slope curved line when settled in descending order. Segments of the curved line exhibiting low slopes are defined by a relatively large number of zircon grains yielding similar ages, revealing consequently events of relatively high zircon production or preservation. Geochronological data from Refs. [1], [4], [5], [6], [7], [8], [9], [10], [11].

Each age datum is first recognized as a concordant age and represented in Fig. 1 as a small circle. They are then settled in descending age order in such a way that all plotted circles delineate a positive-slope curved line. One advantage of this figure is that all age data obtained so far can be visualized at once. Another advantage is that segments of the curved line showing low or high slopes are defined by a relatively large or reduced number of zircon grains yielding similar ages, revealing consequently time periods of relatively high and low melt production or crustal preservation (e.g. Ref. [3]), respectively. Five high-zircon production (or preservation) events (HZE) are identified in this dataset, and assigned, according to their age range, to the (1) Permian, (2) Jurassic, (3) Lower Cretaceous, (4) Paleocene-early Eocene, and (5) late Eocene-Oligocene (Fig. 1).

**Fig. 2:**
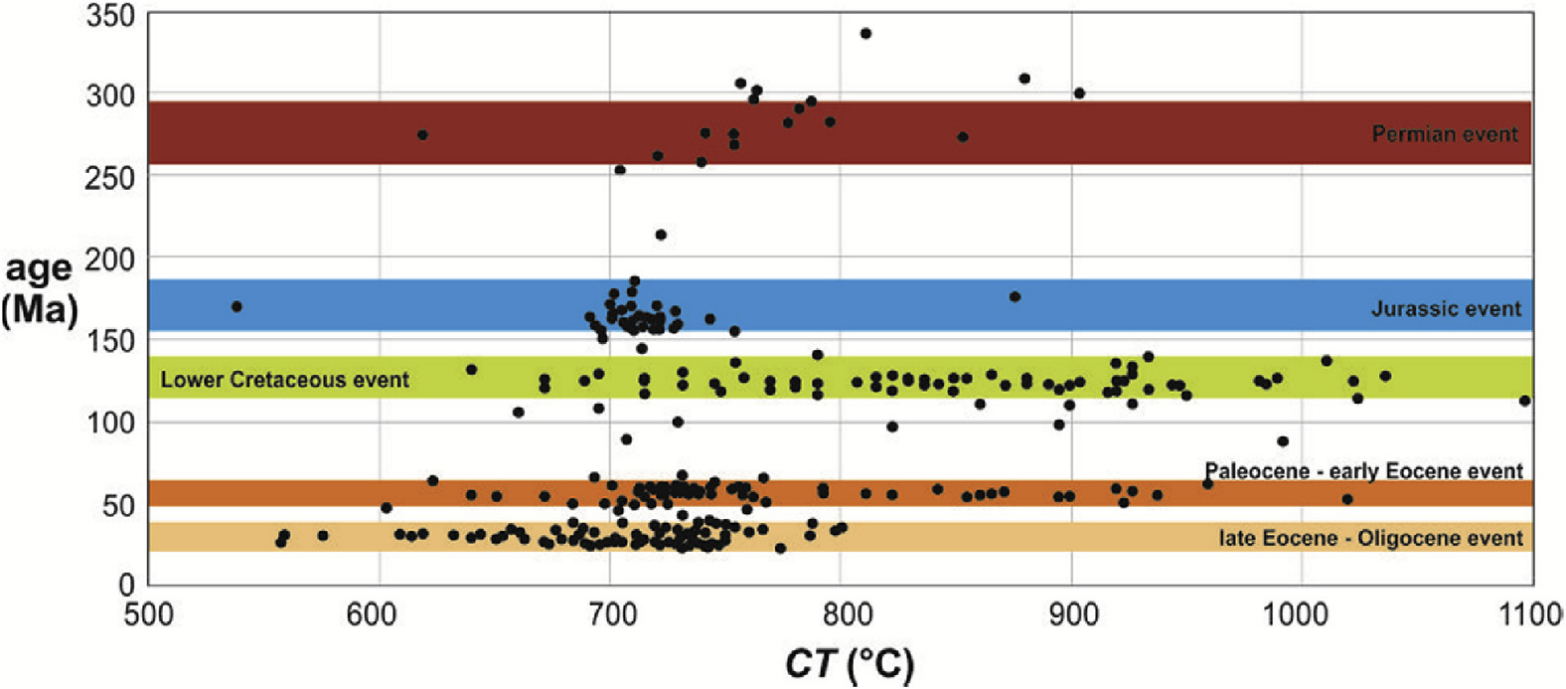
Crystallization temperature (CT) of zircon grains (whose Ti content was acquired) estimated from the modified Ti-in-zircon geothermometer of [2], versus their corresponding U–Pb zircon ages.

Dataset 2 consists of crystallization temperatures (CT) estimated from previously obtained zircon grains whose Ti content was acquired ([1], [8], [10], [11]; Appendix C) using the modified Ti-in-zircon geothermometer of [2]. Crystallization temperature (CT; x-axis) is plot versus the corresponding age of each zircon grain (Fig. 2) to assign them to a HZE. Almost all the zircon grains representing the Permian event show CT between 740.2 and 782.9 °C (except for one zircon showing a relatively low CT of 619.0 °C). Temperature range shown by the Jurassic event (from 42 of 44 grains) is well restrained between 691.4 and 754.8 °C (except for two zircons showing relatively low and high CT of 538.7 and 875.8 °C, respectively); in general, lower than in the Permian event. Zircon grains representing the Lower Cretaceous event exhibit, however, a contrasting wider range of well-distributed CT, from 640.3 to 1036.9 °C (except for a zircon showing an anomalously high temperature of 1733.5 °C not included in Fig. 2). In the case of the Tertiary events, the temperature values shown by the Paleocene-early Eocene event exhibit a wide distribution similar to the Cretaceous event, but within a slightly lower range from 603.3 to 937.4 °C (including a high temperature of 1020.3 °C). Finally, CT of the late Eocene-Oligocene event are lower, and their distribution more confined, than the Paleocene-early Eocene event, bracketed between 557.6 and 800.7 °C.

## 2 Experimental design, materials and methods

### 2.1 Dataset 1

**Fig. 3:**
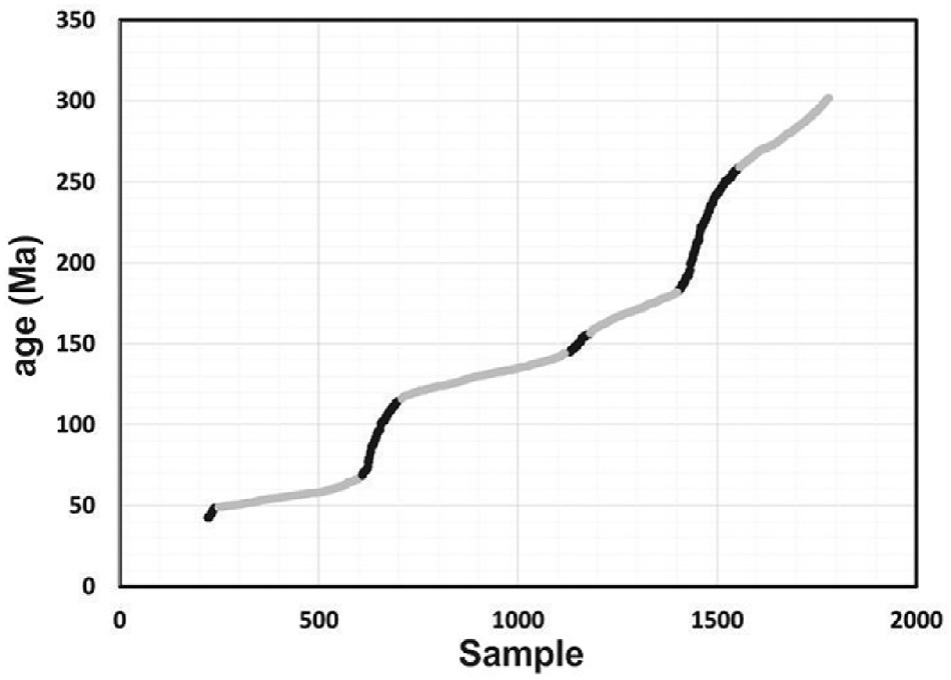
Zircon age of individual grains organized chronologically. X-axis corresponds to sample number in ascendant order while Y-axis is age of the sample (millions of years). Steady flat slope-segments of the curve (in grey) represent events of high zircon production or preservation (HZE).

Dataset 1 was chronologically organized from the most recent through the most ancient. Each data point (individual zircon grain) is referred by a respective sample number (x-axis) and plotted versus its corresponding age (y-axis) in Fig. 3. Flat slope-segments of the curve in grey represent events of HZE. Five flat slope-portions were visually identified according to the staggering of the data.

In order to determine the beginning and end of each segment (and associate it with a geological period of time) we used the following procedure:

1. An average slope of the visual selected portion was calculated. We will call it “segment slope”.
2. A slope every 2 points was also calculated. We will call them “punctual slopes”.
3. We determined the average slope of a moving window every 11, 15 and 21 punctual slopes to smooth isolated peaks. We will call them “window slopes”. We selected the moving window every 21 points because the slopes were smooth enough to follow a pattern but also sensitive enough to identify sudden changes.
4. An error was calculated as the percentile difference between the window slopes and the segment slopes obtained in steps 1 and 3. When the error was greater than 35%, in more than 25 consecutive window slopes, the segment was excluded, otherwise was included.
5. Once the segment was selected, average and standard deviation were calculated (and included in Table 1), and standard deviation was subtracted from the beginning and added to the ending of the segment. The suggested time lapse is also included in Table 1.

**Table 1:** Suggested time lapses from a slope analysis. It shows (in millions of years) the initial segment obtained from slope analysis, the average age of the segment, the standard deviation, and the suggested segment that includes ± σ.

Period/epoch (Segment of the curve)	Beginning - Ending (Slope analysis) (Ma)	Average (Ma)	Standard Deviation σ (Ma)	Suggested event age (Ma) ± σ
Permian	257.2–294.99	275.51	10.26	246.94–305.25
Jurassic	155.18–180.61	169.08	6.98	148.20–187.59
Lower Cretaceous	119.1–140.22	129.93	5.99	113.11–146.21
Paleocene - Early Eocene	49.0–58.05	53.65	2.79	46.21–60.84
Late Eocene - Oligocene	25.91–35.74	30.94	2.88	23.03–38.62

### 2.2 Dataset 2

We used Ti content on individual zircon included in dataset 2 to estimate crystallization temperatures from the modified Ti-in-zircon geothermometer of [2]:

log (ppm Ti-in-zircon) = (5.711 ± 0.0772) – [(4800 ± 86)/T] – log aSiO_2_ + log aTiO_2_

where T is in Kelvin but will be reported hereafter as Celsius, and aTiO_2_ and aSiO_2_ are the activities of titanium oxide and silica in the melt, respectively.

Titanium oxide activity depends on rutile saturation, and silica activity is dependent on quartz saturation. Because quartz is present in all samples, aSiO_2_ was set equal to 1.0 by definition. We do not report rutile in our samples, but titanite is commonly found as an accessory phase, so we assume that aTiO_2_ ≈ 0.6–1.0 during crystallization of zircon, and thus a value of 0.7 for calculations from a compilation reported by Ref. [12] was finally used. These data are comparable to those estimated by Ref. [13] in the Xolapa Complex. Accurate values obtained with the geothermometer used depend, however, on the fact that Ti substitution in zircon should be an equilibrium process (whose occurrence we cannot prove). Similarly, Ti-in-zircon values usually underestimate the crystallization temperature of zircon [14].
