# Supplementary material for: Exploiting individual U–Pb zircon ages and Ti-in-zircon crystallization temperature data to identify high zircon-production events in the Xolapa terrane
Source: Data Brief. 2019 May 2;24:103933. doi: 10.1016/j.dib.2019.103933 (PMC6525283; doi:10.1016/j.dib.2019.103933)
Supplement: Multimedia component 1 [file mmc1.pdf]

Conflict of Interest and Authorship Conformation Form

Please check the following as appropriate:

- All authors have participated in (a) conception and design, or analysis and interpretation of the data; (b) drafting the article or revising it critically for important intellectual content; and (c) approval of the final version.
- This manuscript has not been submitted to, nor is under review at, another journal or other publishing venue.
- The authors have no affiliation with any organization with a direct or indirect financial interest in the subject matter discussed in the manuscript
- The following authors have affiliations with organizations with direct or indirect financial interest in the subject matter discussed in the manuscript: "*Exploiting individual U-Pb zircon ages and Ti-in-zircon crystallization temperature data to identify high zircon-production events in the Xolapa terrane.*" submitted to Data in Brief journal.

Author's name	Affiliation	Signature
Dr. Tomás A. Peña Alonso	UAT	[signature]
Dr. Gina P. Villalobos Escobar	UAT	[signature]
Dr. Roberto S. Molina Garza	CGEO, UNAM	[signature]
