# Supplementary material for: Exploiting individual U–Pb zircon ages and Ti-in-zircon crystallization temperature data to identify high zircon-production events in the Xolapa terrane
Source: Data Brief. 2019 May 2;24:103933. doi: 10.1016/j.dib.2019.103933 (PMC6525283; doi:10.1016/j.dib.2019.103933)
Supplement: Multimedia component 1 [file mmc2.docx]

Appendix A. Age of individual zircon grains from Xolapa samples obtained in the literature

| Sample ID | Rock type | Best age | error | sigma | distance (km) from | Reference |
| --- | --- | --- | --- | --- | --- | --- |
|  |  | (Ma) |  |  | Tecpan to Huatulco |  |
| Xo04-1 | meta-sedimentary | 1127.7 | 34.76 | 2s | 117.35 | Talavera-Mendoza et al. (2013) |
| Xo04-2 | meta-sedimentary | 34.2 | 1.44 | 2s | 117.35 | Talavera-Mendoza et al. (2013) |
| Xo04-3 | meta-sedimentary | 34.7 | 2.85 | 2s | 117.35 | Talavera-Mendoza et al. (2013) |
| Xo04-5 | meta-sedimentary | 242.6 | 20.95 | 2s | 117.35 | Talavera-Mendoza et al. (2013) |
| Xo04-6 | meta-sedimentary | 275.9 | 13.56 | 2s | 117.35 | Talavera-Mendoza et al. (2013) |
| Xo04-7 | meta-sedimentary | 270.7 | 11.69 | 2s | 117.35 | Talavera-Mendoza et al. (2013) |
| Xo04-10 | meta-sedimentary | 35.1 | 2.07 | 2s | 117.35 | Talavera-Mendoza et al. (2013) |
| Xo04-12 | meta-sedimentary | 276.5 | 5.55 | 2s | 117.35 | Talavera-Mendoza et al. (2013) |
| Xo04-13 | meta-sedimentary | 1099.9 | 94.36 | 2s | 117.35 | Talavera-Mendoza et al. (2013) |
| Xo04-14 | meta-sedimentary | 33.6 | 2.80 | 2s | 117.35 | Talavera-Mendoza et al. (2013) |
| Xo04-15 | meta-sedimentary | 33.8 | 1.15 | 2s | 117.35 | Talavera-Mendoza et al. (2013) |
| Xo04-18 | meta-sedimentary | 30.5 | 0.75 | 2s | 117.35 | Talavera-Mendoza et al. (2013) |
| Xo04-20 | meta-sedimentary | 256.6 | 11.77 | 2s | 117.35 | Talavera-Mendoza et al. (2013) |
| Xo04-21 | meta-sedimentary | 34.6 | 2.01 | 2s | 117.35 | Talavera-Mendoza et al. (2013) |
| Xo04-22 | meta-sedimentary | 34.4 | 0.88 | 2s | 117.35 | Talavera-Mendoza et al. (2013) |
| Xo04-23 | meta-sedimentary | 50.4 | 1.92 | 2s | 117.35 | Talavera-Mendoza et al. (2013) |
| Xo04-24 | meta-sedimentary | 34.5 | 0.94 | 2s | 117.35 | Talavera-Mendoza et al. (2013) |
| Xo04-25 | meta-sedimentary | 287.9 | 8.48 | 2s | 117.35 | Talavera-Mendoza et al. (2013) |
| Xo04-29 | meta-sedimentary | 532.7 | 40.70 | 2s | 117.35 | Talavera-Mendoza et al. (2013) |
| Xo04-30 | meta-sedimentary | 269.0 | 12.12 | 2s | 117.35 | Talavera-Mendoza et al. (2013) |
| Xo04-31 | meta-sedimentary | 35.8 | 1.73 | 2s | 117.35 | Talavera-Mendoza et al. (2013) |
| Xo04-35 | meta-sedimentary | 276.0 | 3.73 | 2s | 117.35 | Talavera-Mendoza et al. (2013) |
| Xo04-36 | meta-sedimentary | 35.7 | 2.00 | 2s | 117.35 | Talavera-Mendoza et al. (2013) |
| Xo04-39 | meta-sedimentary | 296.4 | 6.78 | 2s | 117.35 | Talavera-Mendoza et al. (2013) |
| Xo04-40 | meta-sedimentary | 32.8 | 1.55 | 2s | 117.35 | Talavera-Mendoza et al. (2013) |
| Xo04-41 | meta-sedimentary | 283.1 | 13.43 | 2s | 117.35 | Talavera-Mendoza et al. (2013) |
| Xo04-42 | meta-sedimentary | 270.6 | 14.07 | 2s | 117.35 | Talavera-Mendoza et al. (2013) |
| Xo04-43 | meta-sedimentary | 574.4 | 27.37 | 2s | 117.35 | Talavera-Mendoza et al. (2013) |
| Xo04-45 | meta-sedimentary | 284.3 | 4.95 | 2s | 117.35 | Talavera-Mendoza et al. (2013) |
| Xo04-46 | meta-sedimentary | 268.9 | 11.90 | 2s | 117.35 | Talavera-Mendoza et al. (2013) |
| Xo04-47 | meta-sedimentary | 270.3 | 10.75 | 2s | 117.35 | Talavera-Mendoza et al. (2013) |
| Xo04-48 | meta-sedimentary | 455.6 | 13.33 | 2s | 117.35 | Talavera-Mendoza et al. (2013) |
| Xo04-49 | meta-sedimentary | 1050.1 | 44.40 | 2s | 117.35 | Talavera-Mendoza et al. (2013) |
| Xo04-50 | meta-sedimentary | 44.7 | 1.99 | 2s | 117.35 | Talavera-Mendoza et al. (2013) |
| Xo04-57 | meta-sedimentary | 35.9 | 1.10 | 2s | 117.35 | Talavera-Mendoza et al. (2013) |
| Xo04-58 | meta-sedimentary | 282.3 | 6.93 | 2s | 117.35 | Talavera-Mendoza et al. (2013) |
| Xo04-59 | meta-sedimentary | 564.8 | 51.59 | 2s | 117.35 | Talavera-Mendoza et al. (2013) |
| Xo04-61 | meta-sedimentary | 276.5 | 20.27 | 2s | 117.35 | Talavera-Mendoza et al. (2013) |
| Xo04-62 | meta-sedimentary | 39.0 | 2.75 | 2s | 117.35 | Talavera-Mendoza et al. (2013) |
| Xo04-63 | meta-sedimentary | 33.4 | 2.22 | 2s | 117.35 | Talavera-Mendoza et al. (2013) |
| Xo04-64 | meta-sedimentary | 31.7 | 2.66 | 2s | 117.35 | Talavera-Mendoza et al. (2013) |
| Xo04-65 | meta-sedimentary | 35.7 | 0.53 | 2s | 117.35 | Talavera-Mendoza et al. (2013) |
| Xo04-67 | meta-sedimentary | 42.2 | 2.75 | 2s | 117.35 | Talavera-Mendoza et al. (2013) |
| Xo04-70 | meta-sedimentary | 36.6 | 1.56 | 2s | 117.35 | Talavera-Mendoza et al. (2013) |
| Xo04-71 | meta-sedimentary | 33.1 | 1.30 | 2s | 117.35 | Talavera-Mendoza et al. (2013) |
| Xo04-72 | meta-sedimentary | 279.6 | 9.41 | 2s | 117.35 | Talavera-Mendoza et al. (2013) |
| Xo04-74 | meta-sedimentary | 1441.8 | 55.88 | 2s | 117.35 | Talavera-Mendoza et al. (2013) |
| Xo04-75 | meta-sedimentary | 33.8 | 1.81 | 2s | 117.35 | Talavera-Mendoza et al. (2013) |
| Xo04-76 | meta-sedimentary | 36.9 | 1.20 | 2s | 117.35 | Talavera-Mendoza et al. (2013) |
| Xo04-77 | meta-sedimentary | 1249.7 | 24.92 | 2s | 117.35 | Talavera-Mendoza et al. (2013) |
| Xo04-78 | meta-sedimentary | 33.7 | 0.95 | 2s | 117.35 | Talavera-Mendoza et al. (2013) |
| Xo04-79 | meta-sedimentary | 960.4 | 19.90 | 2s | 117.35 | Talavera-Mendoza et al. (2013) |
| Xo04-80 | meta-sedimentary | 303.8 | 12.28 | 2s | 117.35 | Talavera-Mendoza et al. (2013) |
| Xo04-86 | meta-sedimentary | 36.6 | 0.91 | 2s | 117.35 | Talavera-Mendoza et al. (2013) |
| Xo04-87 | meta-sedimentary | 33.9 | 0.98 | 2s | 117.35 | Talavera-Mendoza et al. (2013) |
| Xo04-88 | meta-sedimentary | 521.9 | 14.89 | 2s | 117.35 | Talavera-Mendoza et al. (2013) |
| Xo04-90 | meta-sedimentary | 290.2 | 8.94 | 2s | 117.35 | Talavera-Mendoza et al. (2013) |
| Xo04-94 | meta-sedimentary | 291.0 | 7.34 | 2s | 117.35 | Talavera-Mendoza et al. (2013) |
| Xo04-96 | meta-sedimentary | 34.9 | 1.31 | 2s | 117.35 | Talavera-Mendoza et al. (2013) |
| Xo04-97 | meta-sedimentary | 35.4 | 1.55 | 2s | 117.35 | Talavera-Mendoza et al. (2013) |
| Xo04-98 | meta-sedimentary | 37.4 | 1.41 | 2s | 117.35 | Talavera-Mendoza et al. (2013) |
| Xo04-99 | meta-sedimentary | 293.3 | 4.44 | 2s | 117.35 | Talavera-Mendoza et al. (2013) |
| Xo04-100 | meta-sedimentary | 51.5 | 3.38 | 2s | 117.35 | Talavera-Mendoza et al. (2013) |
| PO111-1 | meta-sedimentary | 1162.0 | 36.35 | 2s | 87.60 | Talavera-Mendoza et al. (2013) |
| PO111-2 | meta-sedimentary | 1124.2 | 25.95 | 2s | 87.60 | Talavera-Mendoza et al. (2013) |
| PO111-3 | meta-sedimentary | 534.3 | 11.90 | 2s | 87.60 | Talavera-Mendoza et al. (2013) |
| PO111-4 | meta-sedimentary | 281.7 | 12.18 | 2s | 87.60 | Talavera-Mendoza et al. (2013) |
| PO111-5 | meta-sedimentary | 2248.4 | 63.09 | 2s | 87.60 | Talavera-Mendoza et al. (2013) |
| PO111-6 | meta-sedimentary | 2088.1 | 45.03 | 2s | 87.60 | Talavera-Mendoza et al. (2013) |
| PO111-7 | meta-sedimentary | 515.7 | 7.98 | 2s | 87.60 | Talavera-Mendoza et al. (2013) |
| PO111-8 | meta-sedimentary | 499.8 | 8.13 | 2s | 87.60 | Talavera-Mendoza et al. (2013) |
| PO111-9 | meta-sedimentary | 2689.3 | 52.57 | 2s | 87.60 | Talavera-Mendoza et al. (2013) |
| PO111-10 | meta-sedimentary | 276.5 | 14.10 | 2s | 87.60 | Talavera-Mendoza et al. (2013) |
| PO111-11 | meta-sedimentary | 240.9 | 4.87 | 2s | 87.60 | Talavera-Mendoza et al. (2013) |
| PO111-13 | meta-sedimentary | 457.0 | 34.77 | 2s | 87.60 | Talavera-Mendoza et al. (2013) |
| PO111-14 | meta-sedimentary | 239.0 | 6.76 | 2s | 87.60 | Talavera-Mendoza et al. (2013) |
| PO111-15 | meta-sedimentary | 318.8 | 3.11 | 2s | 87.60 | Talavera-Mendoza et al. (2013) |
| PO111-16 | meta-sedimentary | 284.4 | 4.31 | 2s | 87.60 | Talavera-Mendoza et al. (2013) |
| PO111-17 | meta-sedimentary | 1093.6 | 53.34 | 2s | 87.60 | Talavera-Mendoza et al. (2013) |
| PO111-18 | meta-sedimentary | 225.7 | 17.83 | 2s | 87.60 | Talavera-Mendoza et al. (2013) |
| PO111-19 | meta-sedimentary | 213.6 | 7.52 | 2s | 87.60 | Talavera-Mendoza et al. (2013) |
| PO111-20 | meta-sedimentary | 264.8 | 4.31 | 2s | 87.60 | Talavera-Mendoza et al. (2013) |
| PO111-21 | meta-sedimentary | 245.3 | 7.44 | 2s | 87.60 | Talavera-Mendoza et al. (2013) |
| PO111-24 | meta-sedimentary | 269.8 | 7.61 | 2s | 87.60 | Talavera-Mendoza et al. (2013) |
| PO111-25 | meta-sedimentary | 293.6 | 4.54 | 2s | 87.60 | Talavera-Mendoza et al. (2013) |
| PO111-26 | meta-sedimentary | 287.2 | 4.10 | 2s | 87.60 | Talavera-Mendoza et al. (2013) |
| PO111-27 | meta-sedimentary | 231.6 | 3.98 | 2s | 87.60 | Talavera-Mendoza et al. (2013) |
| PO111-28 | meta-sedimentary | 254.3 | 2.49 | 2s | 87.60 | Talavera-Mendoza et al. (2013) |
| PO111-29 | meta-sedimentary | 281.1 | 5.61 | 2s | 87.60 | Talavera-Mendoza et al. (2013) |
| PO111-30 | meta-sedimentary | 530.0 | 17.71 | 2s | 87.60 | Talavera-Mendoza et al. (2013) |
| PO111-31 | meta-sedimentary | 270.1 | 3.76 | 2s | 87.60 | Talavera-Mendoza et al. (2013) |
| PO111-32 | meta-sedimentary | 259.1 | 14.93 | 2s | 87.60 | Talavera-Mendoza et al. (2013) |
| PO111-33 | meta-sedimentary | 243.3 | 3.06 | 2s | 87.60 | Talavera-Mendoza et al. (2013) |
| PO111-34 | meta-sedimentary | 236.9 | 5.35 | 2s | 87.60 | Talavera-Mendoza et al. (2013) |
| PO111-35 | meta-sedimentary | 259.4 | 3.74 | 2s | 87.60 | Talavera-Mendoza et al. (2013) |
| PO111-36 | meta-sedimentary | 311.4 | 6.05 | 2s | 87.60 | Talavera-Mendoza et al. (2013) |
| PO111-37 | meta-sedimentary | 1084.6 | 30.98 | 2s | 87.60 | Talavera-Mendoza et al. (2013) |
| PO111-38 | meta-sedimentary | 183.5 | 4.23 | 2s | 87.60 | Talavera-Mendoza et al. (2013) |
| PO111-39 | meta-sedimentary | 264.7 | 2.85 | 2s | 87.60 | Talavera-Mendoza et al. (2013) |
| PO111-41 | meta-sedimentary | 1170.2 | 66.24 | 2s | 87.60 | Talavera-Mendoza et al. (2013) |
| PO111-43 | meta-sedimentary | 1059.5 | 42.94 | 2s | 87.60 | Talavera-Mendoza et al. (2013) |
| PO111-45 | meta-sedimentary | 300.2 | 4.87 | 2s | 87.60 | Talavera-Mendoza et al. (2013) |
| PO111-46 | meta-sedimentary | 289.8 | 8.39 | 2s | 87.60 | Talavera-Mendoza et al. (2013) |
| PO111-47 | meta-sedimentary | 265.7 | 7.44 | 2s | 87.60 | Talavera-Mendoza et al. (2013) |
| PO111-49 | meta-sedimentary | 266.0 | 6.18 | 2s | 87.60 | Talavera-Mendoza et al. (2013) |
| PO111-50 | meta-sedimentary | 1401.9 | 60.76 | 2s | 87.60 | Talavera-Mendoza et al. (2013) |
| PO111-53 | meta-sedimentary | 704.4 | 46.31 | 2s | 87.60 | Talavera-Mendoza et al. (2013) |
| PO111-56 | meta-sedimentary | 250.8 | 5.44 | 2s | 87.60 | Talavera-Mendoza et al. (2013) |
| PO111-57 | meta-sedimentary | 1159.5 | 31.41 | 2s | 87.60 | Talavera-Mendoza et al. (2013) |
| PO111-58 | meta-sedimentary | 266.6 | 2.61 | 2s | 87.60 | Talavera-Mendoza et al. (2013) |
| PO111-59 | meta-sedimentary | 250.7 | 4.45 | 2s | 87.60 | Talavera-Mendoza et al. (2013) |
| PO111-60 | meta-sedimentary | 280.1 | 5.32 | 2s | 87.60 | Talavera-Mendoza et al. (2013) |
| PO111-61 | meta-sedimentary | 1197.3 | 72.54 | 2s | 87.60 | Talavera-Mendoza et al. (2013) |
| PO111-62 | meta-sedimentary | 412.4 | 3.99 | 2s | 87.60 | Talavera-Mendoza et al. (2013) |
| PO111-63 | meta-sedimentary | 387.8 | 6.55 | 2s | 87.60 | Talavera-Mendoza et al. (2013) |
| PO111-64 | meta-sedimentary | 226.0 | 6.59 | 2s | 87.60 | Talavera-Mendoza et al. (2013) |
| PO111-65 | meta-sedimentary | 370.8 | 12.86 | 2s | 87.60 | Talavera-Mendoza et al. (2013) |
| PO111-66 | meta-sedimentary | 242.6 | 18.31 | 2s | 87.60 | Talavera-Mendoza et al. (2013) |
| PO111-67 | meta-sedimentary | 232.2 | 3.92 | 2s | 87.60 | Talavera-Mendoza et al. (2013) |
| PO111-68 | meta-sedimentary | 588.7 | 13.00 | 2s | 87.60 | Talavera-Mendoza et al. (2013) |
| PO111-69 | meta-sedimentary | 937.2 | 15.26 | 2s | 87.60 | Talavera-Mendoza et al. (2013) |
| PO111-70 | meta-sedimentary | 246.1 | 5.50 | 2s | 87.60 | Talavera-Mendoza et al. (2013) |
| PO111-71 | meta-sedimentary | 894.5 | 10.94 | 2s | 87.60 | Talavera-Mendoza et al. (2013) |
| PO111-72 | meta-sedimentary | 1034.0 | 65.69 | 2s | 87.60 | Talavera-Mendoza et al. (2013) |
| PO111-73 | meta-sedimentary | 1594.5 | 31.29 | 2s | 87.60 | Talavera-Mendoza et al. (2013) |
| PO111-74 | meta-sedimentary | 1346.3 | 31.73 | 2s | 87.60 | Talavera-Mendoza et al. (2013) |
| PO111-75 | meta-sedimentary | 276.4 | 4.38 | 2s | 87.60 | Talavera-Mendoza et al. (2013) |
| PO111-76 | meta-sedimentary | 286.3 | 15.35 | 2s | 87.60 | Talavera-Mendoza et al. (2013) |
| PO111-78 | meta-sedimentary | 552.1 | 5.29 | 2s | 87.60 | Talavera-Mendoza et al. (2013) |
| PO111-79 | meta-sedimentary | 264.3 | 4.74 | 2s | 87.60 | Talavera-Mendoza et al. (2013) |
| PO111-80 | meta-sedimentary | 505.7 | 11.48 | 2s | 87.60 | Talavera-Mendoza et al. (2013) |
| PO111-81 | meta-sedimentary | 817.2 | 14.51 | 2s | 87.60 | Talavera-Mendoza et al. (2013) |
| PO111-82 | meta-sedimentary | 527.2 | 11.85 | 2s | 87.60 | Talavera-Mendoza et al. (2013) |
| PO111-83 | meta-sedimentary | 236.8 | 4.32 | 2s | 87.60 | Talavera-Mendoza et al. (2013) |
| PO111-84 | meta-sedimentary | 448.7 | 15.30 | 2s | 87.60 | Talavera-Mendoza et al. (2013) |
| PO111-85 | meta-sedimentary | 259.6 | 4.86 | 2s | 87.60 | Talavera-Mendoza et al. (2013) |
| PO111-86 | meta-sedimentary | 259.8 | 5.91 | 2s | 87.60 | Talavera-Mendoza et al. (2013) |
| PO111-88 | meta-sedimentary | 1464.8 | 41.50 | 2s | 87.60 | Talavera-Mendoza et al. (2013) |
| PO111-90 | meta-sedimentary | 242.4 | 4.71 | 2s | 87.60 | Talavera-Mendoza et al. (2013) |
| PO111-89 | meta-sedimentary | 207.8 | 7.91 | 2s | 87.60 | Talavera-Mendoza et al. (2013) |
| PO111-93 | meta-sedimentary | 259.5 | 7.35 | 2s | 87.60 | Talavera-Mendoza et al. (2013) |
| PO136-1 | meta-sedimentary | 260.7 | 6.92 | 2s | 121.57 | Talavera-Mendoza et al. (2013) |
| PO136-2 | meta-sedimentary | 305.1 | 7.69 | 2s | 121.57 | Talavera-Mendoza et al. (2013) |
| PO136-3 | meta-sedimentary | 260.1 | 2.55 | 2s | 121.57 | Talavera-Mendoza et al. (2013) |
| PO136-4 | meta-sedimentary | 279.7 | 10.32 | 2s | 121.57 | Talavera-Mendoza et al. (2013) |
| PO136-5 | meta-sedimentary | 498.0 | 10.83 | 2s | 121.57 | Talavera-Mendoza et al. (2013) |
| PO136-6 | meta-sedimentary | 257.6 | 6.09 | 2s | 121.57 | Talavera-Mendoza et al. (2013) |
| PO136-7 | meta-sedimentary | 266.3 | 6.63 | 2s | 121.57 | Talavera-Mendoza et al. (2013) |
| PO136-8 | meta-sedimentary | 482.7 | 22.93 | 2s | 121.57 | Talavera-Mendoza et al. (2013) |
| PO136-9 | meta-sedimentary | 249.6 | 4.85 | 2s | 121.57 | Talavera-Mendoza et al. (2013) |
| PO136-10 | meta-sedimentary | 227.3 | 2.35 | 2s | 121.57 | Talavera-Mendoza et al. (2013) |
| PO136-11 | meta-sedimentary | 810.1 | 13.25 | 2s | 121.57 | Talavera-Mendoza et al. (2013) |
| PO136-12 | meta-sedimentary | 256.2 | 10.52 | 2s | 121.57 | Talavera-Mendoza et al. (2013) |
| PO136-13 | meta-sedimentary | 271.3 | 2.66 | 2s | 121.57 | Talavera-Mendoza et al. (2013) |
| PO136-14 | meta-sedimentary | 295.7 | 4.54 | 2s | 121.57 | Talavera-Mendoza et al. (2013) |
| PO136-15 | meta-sedimentary | 250.8 | 10.82 | 2s | 121.57 | Talavera-Mendoza et al. (2013) |
| PO136-16 | meta-sedimentary | 248.6 | 7.46 | 2s | 121.57 | Talavera-Mendoza et al. (2013) |
| PO136-17 | meta-sedimentary | 295.5 | 6.76 | 2s | 121.57 | Talavera-Mendoza et al. (2013) |
| PO136-18 | meta-sedimentary | 132.8 | 1.31 | 2s | 121.57 | Talavera-Mendoza et al. (2013) |
| PO136-19 | meta-sedimentary | 205.5 | 2.02 | 2s | 121.57 | Talavera-Mendoza et al. (2013) |
| PO136-20 | meta-sedimentary | 260.6 | 6.33 | 2s | 121.57 | Talavera-Mendoza et al. (2013) |
| PO136-21 | meta-sedimentary | 306.0 | 8.34 | 2s | 121.57 | Talavera-Mendoza et al. (2013) |
| PO136-22 | meta-sedimentary | 310.7 | 10.89 | 2s | 121.57 | Talavera-Mendoza et al. (2013) |
| PO136-23 | meta-sedimentary | 287.8 | 8.70 | 2s | 121.57 | Talavera-Mendoza et al. (2013) |
| PO136-24 | meta-sedimentary | 277.2 | 2.71 | 2s | 121.57 | Talavera-Mendoza et al. (2013) |
| PO136-25 | meta-sedimentary | 269.2 | 4.88 | 2s | 121.57 | Talavera-Mendoza et al. (2013) |
| PO136-26 | meta-sedimentary | 294.7 | 4.72 | 2s | 121.57 | Talavera-Mendoza et al. (2013) |
| PO136-27 | meta-sedimentary | 271.1 | 6.90 | 2s | 121.57 | Talavera-Mendoza et al. (2013) |
| PO136-29 | meta-sedimentary | 271.1 | 4.25 | 2s | 121.57 | Talavera-Mendoza et al. (2013) |
| PO136-30 | meta-sedimentary | 1183.6 | 37.44 | 2s | 121.57 | Talavera-Mendoza et al. (2013) |
| PO136-31 | meta-sedimentary | 264.0 | 6.21 | 2s | 121.57 | Talavera-Mendoza et al. (2013) |
| PO136-32 | meta-sedimentary | 1146.3 | 26.91 | 2s | 121.57 | Talavera-Mendoza et al. (2013) |
| PO136-33 | meta-sedimentary | 262.3 | 3.24 | 2s | 121.57 | Talavera-Mendoza et al. (2013) |
| PO136-34 | meta-sedimentary | 287.1 | 7.55 | 2s | 121.57 | Talavera-Mendoza et al. (2013) |
| PO136-35 | meta-sedimentary | 302.2 | 7.97 | 2s | 121.57 | Talavera-Mendoza et al. (2013) |
| PO136-36 | meta-sedimentary | 269.7 | 4.44 | 2s | 121.57 | Talavera-Mendoza et al. (2013) |
| PO136-37 | meta-sedimentary | 259.0 | 2.87 | 2s | 121.57 | Talavera-Mendoza et al. (2013) |
| PO136-38 | meta-sedimentary | 280.3 | 8.91 | 2s | 121.57 | Talavera-Mendoza et al. (2013) |
| PO136-40 | meta-sedimentary | 279.9 | 2.74 | 2s | 121.57 | Talavera-Mendoza et al. (2013) |
| PO136-41 | meta-sedimentary | 281.0 | 6.21 | 2s | 121.57 | Talavera-Mendoza et al. (2013) |
| PO136-42 | meta-sedimentary | 274.9 | 4.79 | 2s | 121.57 | Talavera-Mendoza et al. (2013) |
| PO136-43 | meta-sedimentary | 289.0 | 2.83 | 2s | 121.57 | Talavera-Mendoza et al. (2013) |
| PO136-44 | meta-sedimentary | 984.4 | 13.79 | 2s | 121.57 | Talavera-Mendoza et al. (2013) |
| PO136-45 | meta-sedimentary | 281.1 | 5.47 | 2s | 121.57 | Talavera-Mendoza et al. (2013) |
| PO136-46 | meta-sedimentary | 2734.9 | 30.12 | 2s | 121.57 | Talavera-Mendoza et al. (2013) |
| PO136-47 | meta-sedimentary | 1102.3 | 34.02 | 2s | 121.57 | Talavera-Mendoza et al. (2013) |
| PO136-48 | meta-sedimentary | 279.5 | 7.55 | 2s | 121.57 | Talavera-Mendoza et al. (2013) |
| PO136-49 | meta-sedimentary | 284.3 | 2.78 | 2s | 121.57 | Talavera-Mendoza et al. (2013) |
| PO136-50 | meta-sedimentary | 770.0 | 34.40 | 2s | 121.57 | Talavera-Mendoza et al. (2013) |
| PO136-51 | meta-sedimentary | 283.8 | 2.94 | 2s | 121.57 | Talavera-Mendoza et al. (2013) |
| PO136-52 | meta-sedimentary | 268.5 | 4.13 | 2s | 121.57 | Talavera-Mendoza et al. (2013) |
| PO136-53 | meta-sedimentary | 272.6 | 4.88 | 2s | 121.57 | Talavera-Mendoza et al. (2013) |
| PO136-54 | meta-sedimentary | 271.4 | 3.96 | 2s | 121.57 | Talavera-Mendoza et al. (2013) |
| PO136-55 | meta-sedimentary | 488.3 | 17.31 | 2s | 121.57 | Talavera-Mendoza et al. (2013) |
| PO136-56 | meta-sedimentary | 1080.8 | 32.73 | 2s | 121.57 | Talavera-Mendoza et al. (2013) |
| PO136-57 | meta-sedimentary | 242.5 | 6.81 | 2s | 121.57 | Talavera-Mendoza et al. (2013) |
| PO136-58 | meta-sedimentary | 953.3 | 8.86 | 2s | 121.57 | Talavera-Mendoza et al. (2013) |
| PO136-59 | meta-sedimentary | 307.7 | 6.97 | 2s | 121.57 | Talavera-Mendoza et al. (2013) |
| PO136-60 | meta-sedimentary | 308.4 | 3.01 | 2s | 121.57 | Talavera-Mendoza et al. (2013) |
| PO136-61 | meta-sedimentary | 310.9 | 6.16 | 2s | 121.57 | Talavera-Mendoza et al. (2013) |
| PO136-62 | meta-sedimentary | 313.8 | 3.06 | 2s | 121.57 | Talavera-Mendoza et al. (2013) |
| PO136-63 | meta-sedimentary | 277.6 | 6.09 | 2s | 121.57 | Talavera-Mendoza et al. (2013) |
| PO136-64 | meta-sedimentary | 278.4 | 2.72 | 2s | 121.57 | Talavera-Mendoza et al. (2013) |
| PO136-65 | meta-sedimentary | 297.1 | 2.90 | 2s | 121.57 | Talavera-Mendoza et al. (2013) |
| PO136-66 | meta-sedimentary | 266.5 | 10.37 | 2s | 121.57 | Talavera-Mendoza et al. (2013) |
| PO136-67 | meta-sedimentary | 279.6 | 4.65 | 2s | 121.57 | Talavera-Mendoza et al. (2013) |
| PO136-69 | meta-sedimentary | 282.3 | 6.16 | 2s | 121.57 | Talavera-Mendoza et al. (2013) |
| PO136-70 | meta-sedimentary | 248.5 | 9.65 | 2s | 121.57 | Talavera-Mendoza et al. (2013) |
| PO136-71 | meta-sedimentary | 251.5 | 4.12 | 2s | 121.57 | Talavera-Mendoza et al. (2013) |
| PO136-72 | meta-sedimentary | 267.7 | 4.06 | 2s | 121.57 | Talavera-Mendoza et al. (2013) |
| PO136-73 | meta-sedimentary | 285.1 | 4.91 | 2s | 121.57 | Talavera-Mendoza et al. (2013) |
| PO136-74 | meta-sedimentary | 1115.7 | 107.00 | 2s | 121.57 | Talavera-Mendoza et al. (2013) |
| PO136-75 | meta-sedimentary | 192.0 | 5.20 | 2s | 121.57 | Talavera-Mendoza et al. (2013) |
| PO136-76 | meta-sedimentary | 265.4 | 2.96 | 2s | 121.57 | Talavera-Mendoza et al. (2013) |
| PO136-77 | meta-sedimentary | 272.1 | 10.47 | 2s | 121.57 | Talavera-Mendoza et al. (2013) |
| PO136-78 | meta-sedimentary | 270.8 | 8.96 | 2s | 121.57 | Talavera-Mendoza et al. (2013) |
| PO136-79 | meta-sedimentary | 974.6 | 52.22 | 2s | 121.57 | Talavera-Mendoza et al. (2013) |
| PO136-80 | meta-sedimentary | 262.2 | 8.09 | 2s | 121.57 | Talavera-Mendoza et al. (2013) |
| PO136-81 | meta-sedimentary | 272.6 | 2.67 | 2s | 121.57 | Talavera-Mendoza et al. (2013) |
| PO136-82 | meta-sedimentary | 245.0 | 3.87 | 2s | 121.57 | Talavera-Mendoza et al. (2013) |
| PO136-83 | meta-sedimentary | 282.5 | 3.45 | 2s | 121.57 | Talavera-Mendoza et al. (2013) |
| PO136-84 | meta-sedimentary | 273.9 | 4.69 | 2s | 121.57 | Talavera-Mendoza et al. (2013) |
| PO136-85 | meta-sedimentary | 263.1 | 7.68 | 2s | 121.57 | Talavera-Mendoza et al. (2013) |
| PO136-86 | meta-sedimentary | 891.6 | 11.74 | 2s | 121.57 | Talavera-Mendoza et al. (2013) |
| PO136-87 | meta-sedimentary | 280.8 | 6.35 | 2s | 121.57 | Talavera-Mendoza et al. (2013) |
| PO136-88 | meta-sedimentary | 302.8 | 10.68 | 2s | 121.57 | Talavera-Mendoza et al. (2013) |
| PO136-89 | meta-sedimentary | 1124.3 | 67.45 | 2s | 121.57 | Talavera-Mendoza et al. (2013) |
| PO136-90 | meta-sedimentary | 253.8 | 6.22 | 2s | 121.57 | Talavera-Mendoza et al. (2013) |
| PO198-1 | meta-sedimentary | 251.0 | 5.29 | 2s | 89.08 | Talavera-Mendoza et al. (2013) |
| PO198-3 | meta-sedimentary | 393.9 | 23.50 | 2s | 89.08 | Talavera-Mendoza et al. (2013) |
| PO198-4 | meta-sedimentary | 436.6 | 30.35 | 2s | 89.08 | Talavera-Mendoza et al. (2013) |
| PO198-5 | meta-sedimentary | 397.4 | 7.48 | 2s | 89.08 | Talavera-Mendoza et al. (2013) |
| PO198-7 | meta-sedimentary | 396.6 | 19.38 | 2s | 89.08 | Talavera-Mendoza et al. (2013) |
| PO198-8 | meta-sedimentary | 137.8 | 7.43 | 2s | 89.08 | Talavera-Mendoza et al. (2013) |
| PO198-9 | meta-sedimentary | 544.8 | 18.23 | 2s | 89.08 | Talavera-Mendoza et al. (2013) |
| PO198-10 | meta-sedimentary | 95.6 | 9.00 | 2s | 89.08 | Talavera-Mendoza et al. (2013) |
| PO198-11 | meta-sedimentary | 1234.3 | 69.96 | 2s | 89.08 | Talavera-Mendoza et al. (2013) |
| PO198-12 | meta-sedimentary | 504.4 | 5.97 | 2s | 89.08 | Talavera-Mendoza et al. (2013) |
| PO198-13 | meta-sedimentary | 384.0 | 11.93 | 2s | 89.08 | Talavera-Mendoza et al. (2013) |
| PO198-15 | meta-sedimentary | 589.3 | 24.56 | 2s | 89.08 | Talavera-Mendoza et al. (2013) |
| PO198-18 | meta-sedimentary | 436.2 | 13.96 | 2s | 89.08 | Talavera-Mendoza et al. (2013) |
| PO198-19 | meta-sedimentary | 299.9 | 13.42 | 2s | 89.08 | Talavera-Mendoza et al. (2013) |
| PO198-22 | meta-sedimentary | 308.8 | 8.29 | 2s | 89.08 | Talavera-Mendoza et al. (2013) |
| PO198-23 | meta-sedimentary | 460.8 | 9.69 | 2s | 89.08 | Talavera-Mendoza et al. (2013) |
| PO198-26 | meta-sedimentary | 1155.2 | 65.46 | 2s | 89.08 | Talavera-Mendoza et al. (2013) |
| PO198-27 | meta-sedimentary | 715.3 | 19.09 | 2s | 89.08 | Talavera-Mendoza et al. (2013) |
| PO198-28 | meta-sedimentary | 579.1 | 5.54 | 2s | 89.08 | Talavera-Mendoza et al. (2013) |
| PO198-30 | meta-sedimentary | 586.8 | 40.05 | 2s | 89.08 | Talavera-Mendoza et al. (2013) |
| PO198-32 | meta-sedimentary | 1451.0 | 59.15 | 2s | 89.08 | Talavera-Mendoza et al. (2013) |
| PO198-38 | meta-sedimentary | 398.7 | 8.27 | 2s | 89.08 | Talavera-Mendoza et al. (2013) |
| PO198-39 | meta-sedimentary | 436.8 | 10.73 | 2s | 89.08 | Talavera-Mendoza et al. (2013) |
| PO198-40 | meta-sedimentary | 395.7 | 16.20 | 2s | 89.08 | Talavera-Mendoza et al. (2013) |
| PO198-41 | meta-sedimentary | 352.0 | 3.43 | 2s | 89.08 | Talavera-Mendoza et al. (2013) |
| PO198-42 | meta-sedimentary | 848.5 | 11.05 | 2s | 89.08 | Talavera-Mendoza et al. (2013) |
| PO198-45 | meta-sedimentary | 235.9 | 9.19 | 2s | 89.08 | Talavera-Mendoza et al. (2013) |
| PO198-46 | meta-sedimentary | 416.7 | 4.56 | 2s | 89.08 | Talavera-Mendoza et al. (2013) |
| PO198-47 | meta-sedimentary | 1992.6 | 46.82 | 2s | 89.08 | Talavera-Mendoza et al. (2013) |
| PO198-48 | meta-sedimentary | 220.2 | 2.16 | 2s | 89.08 | Talavera-Mendoza et al. (2013) |
| PO198-49 | meta-sedimentary | 535.0 | 26.44 | 2s | 89.08 | Talavera-Mendoza et al. (2013) |
| PO198-50 | meta-sedimentary | 2039.9 | 35.18 | 2s | 89.08 | Talavera-Mendoza et al. (2013) |
| PO198-52 | meta-sedimentary | 483.6 | 6.85 | 2s | 89.08 | Talavera-Mendoza et al. (2013) |
| PO198-53 | meta-sedimentary | 533.8 | 7.48 | 2s | 89.08 | Talavera-Mendoza et al. (2013) |
| PO198-54 | meta-sedimentary | 403.6 | 18.35 | 2s | 89.08 | Talavera-Mendoza et al. (2013) |
| PO198-56 | meta-sedimentary | 400.7 | 10.37 | 2s | 89.08 | Talavera-Mendoza et al. (2013) |
| PO198-57 | meta-sedimentary | 272.1 | 6.39 | 2s | 89.08 | Talavera-Mendoza et al. (2013) |
| PO198-60 | meta-sedimentary | 549.2 | 8.21 | 2s | 89.08 | Talavera-Mendoza et al. (2013) |
| PO198-62 | meta-sedimentary | 547.8 | 9.30 | 2s | 89.08 | Talavera-Mendoza et al. (2013) |
| PO198-63 | meta-sedimentary | 287.2 | 22.30 | 2s | 89.08 | Talavera-Mendoza et al. (2013) |
| PO198-64 | meta-sedimentary | 925.5 | 16.98 | 2s | 89.08 | Talavera-Mendoza et al. (2013) |
| PO198-68 | meta-sedimentary | 309.4 | 3.02 | 2s | 89.08 | Talavera-Mendoza et al. (2013) |
| PO198-69 | meta-sedimentary | 477.2 | 10.58 | 2s | 89.08 | Talavera-Mendoza et al. (2013) |
| PO198-71 | meta-sedimentary | 892.6 | 9.84 | 2s | 89.08 | Talavera-Mendoza et al. (2013) |
| PO198-72 | meta-sedimentary | 560.2 | 7.56 | 2s | 89.08 | Talavera-Mendoza et al. (2013) |
| PO198-73 | meta-sedimentary | 237.5 | 3.20 | 2s | 89.08 | Talavera-Mendoza et al. (2013) |
| PO198-74 | meta-sedimentary | 437.1 | 4.23 | 2s | 89.08 | Talavera-Mendoza et al. (2013) |
| PO198-75 | meta-sedimentary | 399.8 | 33.92 | 2s | 89.08 | Talavera-Mendoza et al. (2013) |
| PO198-78 | meta-sedimentary | 592.7 | 10.59 | 2s | 89.08 | Talavera-Mendoza et al. (2013) |
| PO198-79 | meta-sedimentary | 518.8 | 6.53 | 2s | 89.08 | Talavera-Mendoza et al. (2013) |
| PO198-80 | meta-sedimentary | 462.0 | 20.19 | 2s | 89.08 | Talavera-Mendoza et al. (2013) |
| PO198-81 | meta-sedimentary | 313.3 | 6.67 | 2s | 89.08 | Talavera-Mendoza et al. (2013) |
| PO198-83 | meta-sedimentary | 727.2 | 22.63 | 2s | 89.08 | Talavera-Mendoza et al. (2013) |
| PO198-84 | meta-sedimentary | 299.3 | 3.28 | 2s | 89.08 | Talavera-Mendoza et al. (2013) |
| PO198-85 | meta-sedimentary | 321.7 | 16.76 | 2s | 89.08 | Talavera-Mendoza et al. (2013) |
| PO198-88 | meta-sedimentary | 296.8 | 11.20 | 2s | 89.08 | Talavera-Mendoza et al. (2013) |
| PO198-89 | meta-sedimentary | 549.4 | 49.61 | 2s | 89.08 | Talavera-Mendoza et al. (2013) |
| PO198-91 | meta-sedimentary | 370.1 | 7.63 | 2s | 89.08 | Talavera-Mendoza et al. (2013) |
| PO198-92 | meta-sedimentary | 232.7 | 8.32 | 2s | 89.08 | Talavera-Mendoza et al. (2013) |
| PO198-93 | meta-sedimentary | 473.5 | 4.61 | 2s | 89.08 | Talavera-Mendoza et al. (2013) |
| PO198-94 | meta-sedimentary | 569.3 | 23.27 | 2s | 89.08 | Talavera-Mendoza et al. (2013) |
| PO198-95 | meta-sedimentary | 424.7 | 13.23 | 2s | 89.08 | Talavera-Mendoza et al. (2013) |
| PO198-96 | meta-sedimentary | 522.8 | 35.05 | 2s | 89.08 | Talavera-Mendoza et al. (2013) |
| PO198-98 | meta-sedimentary | 252.4 | 7.30 | 2s | 89.08 | Talavera-Mendoza et al. (2013) |
| PO198-99 | meta-sedimentary | 1204.4 | 29.59 | 2s | 89.08 | Talavera-Mendoza et al. (2013) |
| PO200-1 | meta-sedimentary | 289.6 | 3.28 | 2s | 86.24 | Talavera-Mendoza et al. (2013) |
| PO200-2 | meta-sedimentary | 1274.7 | 26.57 | 2s | 86.24 | Talavera-Mendoza et al. (2013) |
| PO200-3 | meta-sedimentary | 997.1 | 50.91 | 2s | 86.24 | Talavera-Mendoza et al. (2013) |
| PO200-5 | meta-sedimentary | 334.0 | 3.25 | 2s | 86.24 | Talavera-Mendoza et al. (2013) |
| PO200-6 | meta-sedimentary | 853.6 | 12.87 | 2s | 86.24 | Talavera-Mendoza et al. (2013) |
| PO200-7 | meta-sedimentary | 541.2 | 6.80 | 2s | 86.24 | Talavera-Mendoza et al. (2013) |
| PO200-8 | meta-sedimentary | 234.7 | 4.59 | 2s | 86.24 | Talavera-Mendoza et al. (2013) |
| PO200-9 | meta-sedimentary | 926.7 | 13.03 | 2s | 86.24 | Talavera-Mendoza et al. (2013) |
| PO200-10 | meta-sedimentary | 914.6 | 13.13 | 2s | 86.24 | Talavera-Mendoza et al. (2013) |
| PO200-11 | meta-sedimentary | 330.7 | 7.61 | 2s | 86.24 | Talavera-Mendoza et al. (2013) |
| PO200-12 | meta-sedimentary | 490.5 | 4.72 | 2s | 86.24 | Talavera-Mendoza et al. (2013) |
| PO200-13 | meta-sedimentary | 839.0 | 17.78 | 2s | 86.24 | Talavera-Mendoza et al. (2013) |
| PO200-14 | meta-sedimentary | 272.7 | 5.82 | 2s | 86.24 | Talavera-Mendoza et al. (2013) |
| PO200-15 | meta-sedimentary | 1144.3 | 55.94 | 2s | 86.24 | Talavera-Mendoza et al. (2013) |
| PO200-16 | meta-sedimentary | 270.7 | 2.65 | 2s | 86.24 | Talavera-Mendoza et al. (2013) |
| PO200-17 | meta-sedimentary | 292.5 | 5.03 | 2s | 86.24 | Talavera-Mendoza et al. (2013) |
| PO200-18 | meta-sedimentary | 660.4 | 6.28 | 2s | 86.24 | Talavera-Mendoza et al. (2013) |
| PO200-19 | meta-sedimentary | 304.9 | 2.98 | 2s | 86.24 | Talavera-Mendoza et al. (2013) |
| PO200-21 | meta-sedimentary | 279.8 | 12.98 | 2s | 86.24 | Talavera-Mendoza et al. (2013) |
| PO200-22 | meta-sedimentary | 271.1 | 4.43 | 2s | 86.24 | Talavera-Mendoza et al. (2013) |
| PO200-23 | meta-sedimentary | 935.0 | 14.97 | 2s | 86.24 | Talavera-Mendoza et al. (2013) |
| PO200-24 | meta-sedimentary | 1031.6 | 59.68 | 2s | 86.24 | Talavera-Mendoza et al. (2013) |
| PO200-25 | meta-sedimentary | 1004.2 | 38.84 | 2s | 86.24 | Talavera-Mendoza et al. (2013) |
| PO200-26 | meta-sedimentary | 273.9 | 3.81 | 2s | 86.24 | Talavera-Mendoza et al. (2013) |
| PO200-27 | meta-sedimentary | 277.8 | 4.35 | 2s | 86.24 | Talavera-Mendoza et al. (2013) |
| PO200-28 | meta-sedimentary | 144.0 | 13.32 | 2s | 86.24 | Talavera-Mendoza et al. (2013) |
| PO200-29 | meta-sedimentary | 245.5 | 6.55 | 2s | 86.24 | Talavera-Mendoza et al. (2013) |
| PO200-31 | meta-sedimentary | 269.5 | 7.39 | 2s | 86.24 | Talavera-Mendoza et al. (2013) |
| PO200-32 | meta-sedimentary | 794.1 | 33.02 | 2s | 86.24 | Talavera-Mendoza et al. (2013) |
| PO200-33 | meta-sedimentary | 249.1 | 5.52 | 2s | 86.24 | Talavera-Mendoza et al. (2013) |
| PO200-34 | meta-sedimentary | 1110.2 | 28.65 | 2s | 86.24 | Talavera-Mendoza et al. (2013) |
| PO200-35 | meta-sedimentary | 961.2 | 19.82 | 2s | 86.24 | Talavera-Mendoza et al. (2013) |
| PO200-36 | meta-sedimentary | 232.6 | 3.88 | 2s | 86.24 | Talavera-Mendoza et al. (2013) |
| PO200-37 | meta-sedimentary | 253.3 | 4.42 | 2s | 86.24 | Talavera-Mendoza et al. (2013) |
| PO200-38 | meta-sedimentary | 1174.5 | 20.30 | 2s | 86.24 | Talavera-Mendoza et al. (2013) |
| PO200-39 | meta-sedimentary | 1181.9 | 34.65 | 2s | 86.24 | Talavera-Mendoza et al. (2013) |
| PO200-40 | meta-sedimentary | 248.7 | 4.29 | 2s | 86.24 | Talavera-Mendoza et al. (2013) |
| PO200-41 | meta-sedimentary | 279.8 | 9.17 | 2s | 86.24 | Talavera-Mendoza et al. (2013) |
| PO200-42 | meta-sedimentary | 302.7 | 2.96 | 2s | 86.24 | Talavera-Mendoza et al. (2013) |
| PO200-43 | meta-sedimentary | 277.4 | 3.42 | 2s | 86.24 | Talavera-Mendoza et al. (2013) |
| PO200-44 | meta-sedimentary | 948.6 | 50.22 | 2s | 86.24 | Talavera-Mendoza et al. (2013) |
| PO200-45 | meta-sedimentary | 295.0 | 3.86 | 2s | 86.24 | Talavera-Mendoza et al. (2013) |
| PO200-46 | meta-sedimentary | 319.8 | 3.12 | 2s | 86.24 | Talavera-Mendoza et al. (2013) |
| PO200-47 | meta-sedimentary | 480.0 | 6.57 | 2s | 86.24 | Talavera-Mendoza et al. (2013) |
| PO200-48 | meta-sedimentary | 1306.8 | 32.25 | 2s | 86.24 | Talavera-Mendoza et al. (2013) |
| PO200-49 | meta-sedimentary | 398.7 | 11.17 | 2s | 86.24 | Talavera-Mendoza et al. (2013) |
| PO200-50 | meta-sedimentary | 460.9 | 9.92 | 2s | 86.24 | Talavera-Mendoza et al. (2013) |
| PO200-51 | meta-sedimentary | 262.9 | 7.39 | 2s | 86.24 | Talavera-Mendoza et al. (2013) |
| PO200-52 | meta-sedimentary | 893.4 | 29.95 | 2s | 86.24 | Talavera-Mendoza et al. (2013) |
| PO200-53 | meta-sedimentary | 270.0 | 4.76 | 2s | 86.24 | Talavera-Mendoza et al. (2013) |
| PO200-54 | meta-sedimentary | 1329.0 | 22.71 | 2s | 86.24 | Talavera-Mendoza et al. (2013) |
| PO200-55 | meta-sedimentary | 266.7 | 4.89 | 2s | 86.24 | Talavera-Mendoza et al. (2013) |
| PO200-56 | meta-sedimentary | 749.6 | 28.09 | 2s | 86.24 | Talavera-Mendoza et al. (2013) |
| PO200-57 | meta-sedimentary | 267.7 | 2.62 | 2s | 86.24 | Talavera-Mendoza et al. (2013) |
| PO200-58 | meta-sedimentary | 170.6 | 6.08 | 2s | 86.24 | Talavera-Mendoza et al. (2013) |
| PO200-59 | meta-sedimentary | 236.1 | 3.13 | 2s | 86.24 | Talavera-Mendoza et al. (2013) |
| PO200-60 | meta-sedimentary | 264.6 | 2.59 | 2s | 86.24 | Talavera-Mendoza et al. (2013) |
| PO200-61 | meta-sedimentary | 388.4 | 3.77 | 2s | 86.24 | Talavera-Mendoza et al. (2013) |
| PO200-62 | meta-sedimentary | 264.7 | 4.02 | 2s | 86.24 | Talavera-Mendoza et al. (2013) |
| PO200-63 | meta-sedimentary | 580.8 | 5.55 | 2s | 86.24 | Talavera-Mendoza et al. (2013) |
| PO200-64 | meta-sedimentary | 1220.5 | 33.67 | 2s | 86.24 | Talavera-Mendoza et al. (2013) |
| PO200-65 | meta-sedimentary | 256.7 | 11.50 | 2s | 86.24 | Talavera-Mendoza et al. (2013) |
| PO200-66 | meta-sedimentary | 258.7 | 7.56 | 2s | 86.24 | Talavera-Mendoza et al. (2013) |
| PO200-67 | meta-sedimentary | 257.1 | 5.87 | 2s | 86.24 | Talavera-Mendoza et al. (2013) |
| PO200-68 | meta-sedimentary | 272.3 | 3.17 | 2s | 86.24 | Talavera-Mendoza et al. (2013) |
| PO200-69 | meta-sedimentary | 272.5 | 3.12 | 2s | 86.24 | Talavera-Mendoza et al. (2013) |
| PO200-70 | meta-sedimentary | 883.8 | 8.26 | 2s | 86.24 | Talavera-Mendoza et al. (2013) |
| PO200-72 | meta-sedimentary | 252.4 | 5.27 | 2s | 86.24 | Talavera-Mendoza et al. (2013) |
| PO200-73 | meta-sedimentary | 741.1 | 12.67 | 2s | 86.24 | Talavera-Mendoza et al. (2013) |
| PO200-74 | meta-sedimentary | 267.8 | 4.98 | 2s | 86.24 | Talavera-Mendoza et al. (2013) |
| PO200-75 | meta-sedimentary | 250.8 | 4.43 | 2s | 86.24 | Talavera-Mendoza et al. (2013) |
| PO200-76 | meta-sedimentary | 250.6 | 3.00 | 2s | 86.24 | Talavera-Mendoza et al. (2013) |
| PO200-77 | meta-sedimentary | 252.7 | 3.82 | 2s | 86.24 | Talavera-Mendoza et al. (2013) |
| PO200-79 | meta-sedimentary | 586.4 | 17.21 | 2s | 86.24 | Talavera-Mendoza et al. (2013) |
| PO200-81 | meta-sedimentary | 279.6 | 5.96 | 2s | 86.24 | Talavera-Mendoza et al. (2013) |
| PO200-83 | meta-sedimentary | 301.5 | 2.95 | 2s | 86.24 | Talavera-Mendoza et al. (2013) |
| PO200-84 | meta-sedimentary | 304.7 | 8.84 | 2s | 86.24 | Talavera-Mendoza et al. (2013) |
| PO200-85 | meta-sedimentary | 270.9 | 6.50 | 2s | 86.24 | Talavera-Mendoza et al. (2013) |
| PO200-86 | meta-sedimentary | 279.1 | 4.07 | 2s | 86.24 | Talavera-Mendoza et al. (2013) |
| PO200-87 | meta-sedimentary | 896.2 | 12.05 | 2s | 86.24 | Talavera-Mendoza et al. (2013) |
| PO200-88 | meta-sedimentary | 629.7 | 15.96 | 2s | 86.24 | Talavera-Mendoza et al. (2013) |
| PO200-89 | meta-sedimentary | 283.2 | 3.77 | 2s | 86.24 | Talavera-Mendoza et al. (2013) |
| PO200-90 | meta-sedimentary | 263.0 | 5.21 | 2s | 86.24 | Talavera-Mendoza et al. (2013) |
| PO200-91 | meta-sedimentary | 1339.3 | 27.26 | 2s | 86.24 | Talavera-Mendoza et al. (2013) |
| PO200-92 | meta-sedimentary | 1678.0 | 70.86 | 2s | 86.24 | Talavera-Mendoza et al. (2013) |
| PO200-93 | meta-sedimentary | 1240.5 | 21.73 | 2s | 86.24 | Talavera-Mendoza et al. (2013) |
| PO200-94 | meta-sedimentary | 301.5 | 3.50 | 2s | 86.24 | Talavera-Mendoza et al. (2013) |
| PO200-95 | meta-sedimentary | 302.6 | 2.96 | 2s | 86.24 | Talavera-Mendoza et al. (2013) |
| PO200-96 | meta-sedimentary | 279.2 | 6.18 | 2s | 86.24 | Talavera-Mendoza et al. (2013) |
| PO200-97 | meta-sedimentary | 1115.1 | 63.70 | 2s | 86.24 | Talavera-Mendoza et al. (2013) |
| PO200-98 | meta-sedimentary | 285.2 | 2.79 | 2s | 86.24 | Talavera-Mendoza et al. (2013) |
| PO200-99 | meta-sedimentary | 272.0 | 5.46 | 2s | 86.24 | Talavera-Mendoza et al. (2013) |
| PO200-100 | meta-sedimentary | 1410.7 | 19.37 | 2s | 86.24 | Talavera-Mendoza et al. (2013) |
| PO201-1 | meta-sedimentary | 298.9 | 3.53 | 2s | 235.97 | Talavera-Mendoza et al. (2013) |
| PO201-2 | meta-sedimentary | 1157.0 | 28.41 | 2s | 235.97 | Talavera-Mendoza et al. (2013) |
| PO201-3 | meta-sedimentary | 1040.8 | 33.73 | 2s | 235.97 | Talavera-Mendoza et al. (2013) |
| PO201-4 | meta-sedimentary | 286.7 | 2.80 | 2s | 235.97 | Talavera-Mendoza et al. (2013) |
| PO201-5 | meta-sedimentary | 907.4 | 38.85 | 2s | 235.97 | Talavera-Mendoza et al. (2013) |
| PO201-7 | meta-sedimentary | 784.1 | 11.07 | 2s | 235.97 | Talavera-Mendoza et al. (2013) |
| PO201-8 | meta-sedimentary | 796.8 | 17.16 | 2s | 235.97 | Talavera-Mendoza et al. (2013) |
| PO201-9 | meta-sedimentary | 572.6 | 24.60 | 2s | 235.97 | Talavera-Mendoza et al. (2013) |
| PO201-11 | meta-sedimentary | 1237.2 | 64.62 | 2s | 235.97 | Talavera-Mendoza et al. (2013) |
| PO201-12 | meta-sedimentary | 213.6 | 4.45 | 2s | 235.97 | Talavera-Mendoza et al. (2013) |
| PO201-13 | meta-sedimentary | 1177.5 | 44.91 | 2s | 235.97 | Talavera-Mendoza et al. (2013) |
| PO201-14 | meta-sedimentary | 262.9 | 4.74 | 2s | 235.97 | Talavera-Mendoza et al. (2013) |
| PO201-15 | meta-sedimentary | 270.5 | 3.63 | 2s | 235.97 | Talavera-Mendoza et al. (2013) |
| PO201-17 | meta-sedimentary | 1289.3 | 70.29 | 2s | 235.97 | Talavera-Mendoza et al. (2013) |
| PO201-18 | meta-sedimentary | 285.0 | 2.79 | 2s | 235.97 | Talavera-Mendoza et al. (2013) |
| PO201-20 | meta-sedimentary | 1262.5 | 37.19 | 2s | 235.97 | Talavera-Mendoza et al. (2013) |
| PO201-21 | meta-sedimentary | 1138.2 | 45.37 | 2s | 235.97 | Talavera-Mendoza et al. (2013) |
| PO201-23 | meta-sedimentary | 1200.8 | 47.92 | 2s | 235.97 | Talavera-Mendoza et al. (2013) |
| PO201-24 | meta-sedimentary | 243.3 | 5.56 | 2s | 235.97 | Talavera-Mendoza et al. (2013) |
| PO201-25 | meta-sedimentary | 1045.2 | 27.17 | 2s | 235.97 | Talavera-Mendoza et al. (2013) |
| PO201-27 | meta-sedimentary | 893.4 | 27.78 | 2s | 235.97 | Talavera-Mendoza et al. (2013) |
| PO201-26 | meta-sedimentary | 289.4 | 3.65 | 2s | 235.97 | Talavera-Mendoza et al. (2013) |
| PO201-28 | meta-sedimentary | 194.1 | 8.53 | 2s | 235.97 | Talavera-Mendoza et al. (2013) |
| PO201-29 | meta-sedimentary | 535.7 | 42.41 | 2s | 235.97 | Talavera-Mendoza et al. (2013) |
| PO201-30 | meta-sedimentary | 895.1 | 8.36 | 2s | 235.97 | Talavera-Mendoza et al. (2013) |
| PO201-31 | meta-sedimentary | 1080.3 | 51.02 | 2s | 235.97 | Talavera-Mendoza et al. (2013) |
| PO201-32 | meta-sedimentary | 1155.9 | 32.58 | 2s | 235.97 | Talavera-Mendoza et al. (2013) |
| PO201-33 | meta-sedimentary | 214.0 | 7.51 | 2s | 235.97 | Talavera-Mendoza et al. (2013) |
| PO201-36 | meta-sedimentary | 1248.7 | 40.54 | 2s | 235.97 | Talavera-Mendoza et al. (2013) |
| PO201-37 | meta-sedimentary | 1141.3 | 28.05 | 2s | 235.97 | Talavera-Mendoza et al. (2013) |
| PO201-38 | meta-sedimentary | 298.0 | 4.19 | 2s | 235.97 | Talavera-Mendoza et al. (2013) |
| PO201-39 | meta-sedimentary | 988.1 | 65.29 | 2s | 235.97 | Talavera-Mendoza et al. (2013) |
| PO201-40 | meta-sedimentary | 766.6 | 22.62 | 2s | 235.97 | Talavera-Mendoza et al. (2013) |
| PO201-41 | meta-sedimentary | 283.0 | 4.49 | 2s | 235.97 | Talavera-Mendoza et al. (2013) |
| PO201-42 | meta-sedimentary | 210.6 | 5.95 | 2s | 235.97 | Talavera-Mendoza et al. (2013) |
| PO201-43 | meta-sedimentary | 352.2 | 3.43 | 2s | 235.97 | Talavera-Mendoza et al. (2013) |
| PO201-44 | meta-sedimentary | 292.4 | 6.38 | 2s | 235.97 | Talavera-Mendoza et al. (2013) |
| PO201-46 | meta-sedimentary | 70.3 | 0.75 | 2s | 235.97 | Talavera-Mendoza et al. (2013) |
| PO201-47 | meta-sedimentary | 71.2 | 0.93 | 2s | 235.97 | Talavera-Mendoza et al. (2013) |
| PO201-48 | meta-sedimentary | 1031.3 | 45.71 | 2s | 235.97 | Talavera-Mendoza et al. (2013) |
| PO201-49 | meta-sedimentary | 573.8 | 9.22 | 2s | 235.97 | Talavera-Mendoza et al. (2013) |
| PO201-50 | meta-sedimentary | 1001.4 | 45.12 | 2s | 235.97 | Talavera-Mendoza et al. (2013) |
| PO201-51 | meta-sedimentary | 419.0 | 25.19 | 2s | 235.97 | Talavera-Mendoza et al. (2013) |
| PO201-52 | meta-sedimentary | 343.4 | 10.33 | 2s | 235.97 | Talavera-Mendoza et al. (2013) |
| PO201-53 | meta-sedimentary | 1055.5 | 43.59 | 2s | 235.97 | Talavera-Mendoza et al. (2013) |
| PO201-54 | meta-sedimentary | 1363.0 | 49.15 | 2s | 235.97 | Talavera-Mendoza et al. (2013) |
| PO201-55 | meta-sedimentary | 988.5 | 29.12 | 2s | 235.97 | Talavera-Mendoza et al. (2013) |
| PO201-56 | meta-sedimentary | 1164.1 | 35.32 | 2s | 235.97 | Talavera-Mendoza et al. (2013) |
| PO201-57 | meta-sedimentary | 165.4 | 11.99 | 2s | 235.97 | Talavera-Mendoza et al. (2013) |
| PO201-58 | meta-sedimentary | 368.0 | 19.78 | 2s | 235.97 | Talavera-Mendoza et al. (2013) |
| PO201-59 | meta-sedimentary | 272.7 | 5.95 | 2s | 235.97 | Talavera-Mendoza et al. (2013) |
| PO201-60 | meta-sedimentary | 1246.0 | 40.87 | 2s | 235.97 | Talavera-Mendoza et al. (2013) |
| PO201-62 | meta-sedimentary | 297.4 | 4.59 | 2s | 235.97 | Talavera-Mendoza et al. (2013) |
| PO201-64 | meta-sedimentary | 1072.1 | 52.84 | 2s | 235.97 | Talavera-Mendoza et al. (2013) |
| PO201-65 | meta-sedimentary | 1109.0 | 39.42 | 2s | 235.97 | Talavera-Mendoza et al. (2013) |
| PO201-66 | meta-sedimentary | 290.2 | 3.32 | 2s | 235.97 | Talavera-Mendoza et al. (2013) |
| PO201-68 | meta-sedimentary | 1185.3 | 52.46 | 2s | 235.97 | Talavera-Mendoza et al. (2013) |
| PO201-69 | meta-sedimentary | 271.6 | 6.30 | 2s | 235.97 | Talavera-Mendoza et al. (2013) |
| PO201-70 | meta-sedimentary | 289.9 | 3.37 | 2s | 235.97 | Talavera-Mendoza et al. (2013) |
| PO201-71 | meta-sedimentary | 1084.5 | 65.36 | 2s | 235.97 | Talavera-Mendoza et al. (2013) |
| PO201-73 | meta-sedimentary | 1231.4 | 35.52 | 2s | 235.97 | Talavera-Mendoza et al. (2013) |
| PO201-74 | meta-sedimentary | 832.1 | 64.64 | 2s | 235.97 | Talavera-Mendoza et al. (2013) |
| PO201-75 | meta-sedimentary | 1322.0 | 39.55 | 2s | 235.97 | Talavera-Mendoza et al. (2013) |
| PO201-76 | meta-sedimentary | 269.6 | 2.83 | 2s | 235.97 | Talavera-Mendoza et al. (2013) |
| PO201-77 | meta-sedimentary | 869.3 | 10.57 | 2s | 235.97 | Talavera-Mendoza et al. (2013) |
| PO201-79 | meta-sedimentary | 1208.8 | 49.85 | 2s | 235.97 | Talavera-Mendoza et al. (2013) |
| PO201-80 | meta-sedimentary | 1054.5 | 82.84 | 2s | 235.97 | Talavera-Mendoza et al. (2013) |
| PO201-81 | meta-sedimentary | 551.6 | 5.60 | 2s | 235.97 | Talavera-Mendoza et al. (2013) |
| PO201-82 | meta-sedimentary | 1160.2 | 30.14 | 2s | 235.97 | Talavera-Mendoza et al. (2013) |
| PO201-83 | meta-sedimentary | 1139.5 | 39.81 | 2s | 235.97 | Talavera-Mendoza et al. (2013) |
| PO201-84 | meta-sedimentary | 1750.8 | 23.44 | 2s | 235.97 | Talavera-Mendoza et al. (2013) |
| PO201-86 | meta-sedimentary | 1217.0 | 74.78 | 2s | 235.97 | Talavera-Mendoza et al. (2013) |
| PO201-87 | meta-sedimentary | 497.6 | 23.52 | 2s | 235.97 | Talavera-Mendoza et al. (2013) |
| PO201-88 | meta-sedimentary | 240.4 | 9.02 | 2s | 235.97 | Talavera-Mendoza et al. (2013) |
| PO201-90 | meta-sedimentary | 1065.8 | 75.85 | 2s | 235.97 | Talavera-Mendoza et al. (2013) |
| PO201-91 | meta-sedimentary | 1135.6 | 53.88 | 2s | 235.97 | Talavera-Mendoza et al. (2013) |
| PO201-92 | meta-sedimentary | 539.7 | 19.36 | 2s | 235.97 | Talavera-Mendoza et al. (2013) |
| PO201-94 | meta-sedimentary | 222.4 | 6.08 | 2s | 235.97 | Talavera-Mendoza et al. (2013) |
| PO201-95 | meta-sedimentary | 243.2 | 4.82 | 2s | 235.97 | Talavera-Mendoza et al. (2013) |
| PO201-96 | meta-sedimentary | 328.2 | 3.20 | 2s | 235.97 | Talavera-Mendoza et al. (2013) |
| PO201-97 | meta-sedimentary | 222.0 | 6.44 | 2s | 235.97 | Talavera-Mendoza et al. (2013) |
| PO201-98 | meta-sedimentary | 1144.5 | 31.90 | 2s | 235.97 | Talavera-Mendoza et al. (2013) |
| PO201-99 | meta-sedimentary | 1250.8 | 27.00 | 2s | 235.97 | Talavera-Mendoza et al. (2013) |
| PO201-100 | meta-sedimentary | 268.5 | 15.36 | 2s | 235.97 | Talavera-Mendoza et al. (2013) |
| PO201-101 | meta-sedimentary | 1273.5 | 46.15 | 2s | 235.97 | Talavera-Mendoza et al. (2013) |
| P=201-102 | meta-sedimentary | 1108.8 | 57.36 | 2s | 235.97 | Talavera-Mendoza et al. (2013) |
| PO201-104 | meta-sedimentary | 1103.2 | 39.21 | 2s | 235.97 | Talavera-Mendoza et al. (2013) |
| PO201-106 | meta-sedimentary | 302.4 | 9.96 | 2s | 235.97 | Talavera-Mendoza et al. (2013) |
| PO201-107 | meta-sedimentary | 1198.8 | 24.31 | 2s | 235.97 | Talavera-Mendoza et al. (2013) |
| PO201-108 | meta-sedimentary | 251.8 | 5.09 | 2s | 235.97 | Talavera-Mendoza et al. (2013) |
| PO201-109 | meta-sedimentary | 270.1 | 7.25 | 2s | 235.97 | Talavera-Mendoza et al. (2013) |
| PO201-110 | meta-sedimentary | 304.2 | 5.14 | 2s | 235.97 | Talavera-Mendoza et al. (2013) |
| PO298-1 | meta-sedimentary | 228.7 | 11.11 | 2s | 250.01 | Talavera-Mendoza et al. (2013) |
| PO298-2 | meta-sedimentary | 261.8 | 1.74 | 2s | 250.01 | Talavera-Mendoza et al. (2013) |
| PO298-3 | meta-sedimentary | 209.0 | 11.74 | 2s | 250.01 | Talavera-Mendoza et al. (2013) |
| PO298-4 | meta-sedimentary | 60.4 | 3.08 | 2s | 250.01 | Talavera-Mendoza et al. (2013) |
| PO298-6 | meta-sedimentary | 429.9 | 7.31 | 2s | 250.01 | Talavera-Mendoza et al. (2013) |
| PO298-8 | meta-sedimentary | 494.5 | 6.16 | 2s | 250.01 | Talavera-Mendoza et al. (2013) |
| PO298-9 | meta-sedimentary | 533.1 | 27.94 | 2s | 250.01 | Talavera-Mendoza et al. (2013) |
| PO298-10 | meta-sedimentary | 327.4 | 7.16 | 2s | 250.01 | Talavera-Mendoza et al. (2013) |
| PO298-11 | meta-sedimentary | 255.1 | 17.09 | 2s | 250.01 | Talavera-Mendoza et al. (2013) |
| PO298-13 | meta-sedimentary | 225.8 | 5.26 | 2s | 250.01 | Talavera-Mendoza et al. (2013) |
| PO298-14 | meta-sedimentary | 228.3 | 7.30 | 2s | 250.01 | Talavera-Mendoza et al. (2013) |
| PO298-15 | meta-sedimentary | 1122.9 | 8.53 | 2s | 250.01 | Talavera-Mendoza et al. (2013) |
| PO298-16 | meta-sedimentary | 267.0 | 3.77 | 2s | 250.01 | Talavera-Mendoza et al. (2013) |
| PO298-17 | meta-sedimentary | 247.7 | 3.98 | 2s | 250.01 | Talavera-Mendoza et al. (2013) |
| PO298-18 | meta-sedimentary | 202.2 | 4.62 | 2s | 250.01 | Talavera-Mendoza et al. (2013) |
| PO298-19 | meta-sedimentary | 260.8 | 1.92 | 2s | 250.01 | Talavera-Mendoza et al. (2013) |
| PO298-20 | meta-sedimentary | 137.6 | 4.45 | 2s | 250.01 | Talavera-Mendoza et al. (2013) |
| PO298-21 | meta-sedimentary | 261.1 | 9.94 | 2s | 250.01 | Talavera-Mendoza et al. (2013) |
| PO298-22 | meta-sedimentary | 60.8 | 0.72 | 2s | 250.01 | Talavera-Mendoza et al. (2013) |
| PO298-23 | meta-sedimentary | 292.6 | 4.39 | 2s | 250.01 | Talavera-Mendoza et al. (2013) |
| PO298-24 | meta-sedimentary | 1011.7 | 44.75 | 2s | 250.01 | Talavera-Mendoza et al. (2013) |
| PO298-25 | meta-sedimentary | 275.5 | 2.45 | 2s | 250.01 | Talavera-Mendoza et al. (2013) |
| PO298-26 | meta-sedimentary | 275.2 | 3.77 | 2s | 250.01 | Talavera-Mendoza et al. (2013) |
| PO298-27 | meta-sedimentary | 53.2 | 10.95 | 2s | 250.01 | Talavera-Mendoza et al. (2013) |
| PO298-28 | meta-sedimentary | 1094.6 | 31.54 | 2s | 250.01 | Talavera-Mendoza et al. (2013) |
| PO298-29 | meta-sedimentary | 261.4 | 2.90 | 2s | 250.01 | Talavera-Mendoza et al. (2013) |
| PO298-31 | meta-sedimentary | 289.2 | 6.30 | 2s | 250.01 | Talavera-Mendoza et al. (2013) |
| PO298-32 | meta-sedimentary | 246.2 | 4.45 | 2s | 250.01 | Talavera-Mendoza et al. (2013) |
| PO298-33 | meta-sedimentary | 79.8 | 6.71 | 2s | 250.01 | Talavera-Mendoza et al. (2013) |
| PO298-35 | meta-sedimentary | 274.6 | 8.40 | 2s | 250.01 | Talavera-Mendoza et al. (2013) |
| P0204-1 | meta-sedimentary | 557.8 | 7.75 | 2s | 291.51 | Talavera-Mendoza et al. (2013) |
| P0204-2 | meta-sedimentary | 1278.3 | 46.02 | 2s | 291.51 | Talavera-Mendoza et al. (2013) |
| P0204-3 | meta-sedimentary | 58.5 | 0.64 | 2s | 291.51 | Talavera-Mendoza et al. (2013) |
| P0204-4 | meta-sedimentary | 33.2 | 0.33 | 2s | 291.51 | Talavera-Mendoza et al. (2013) |
| P0204-5 | meta-sedimentary | 601.4 | 5.74 | 2s | 291.51 | Talavera-Mendoza et al. (2013) |
| P0204-6 | meta-sedimentary | 394.8 | 10.26 | 2s | 291.51 | Talavera-Mendoza et al. (2013) |
| P0204-7 | meta-sedimentary | 437.9 | 4.23 | 2s | 291.51 | Talavera-Mendoza et al. (2013) |
| P0204-8 | meta-sedimentary | 491.0 | 15.89 | 2s | 291.51 | Talavera-Mendoza et al. (2013) |
| P0204-9 | meta-sedimentary | 657.1 | 6.25 | 2s | 291.51 | Talavera-Mendoza et al. (2013) |
| P0204-13 | meta-sedimentary | 60.3 | 1.85 | 2s | 291.51 | Talavera-Mendoza et al. (2013) |
| P0204-12 | meta-sedimentary | 63.1 | 0.91 | 2s | 291.51 | Talavera-Mendoza et al. (2013) |
| P0204-15 | meta-sedimentary | 368.5 | 15.58 | 2s | 291.51 | Talavera-Mendoza et al. (2013) |
| P0204-17 | meta-sedimentary | 362.5 | 32.46 | 2s | 291.51 | Talavera-Mendoza et al. (2013) |
| P0204-18 | meta-sedimentary | 365.0 | 27.39 | 2s | 291.51 | Talavera-Mendoza et al. (2013) |
| P0204-19 | meta-sedimentary | 34.6 | 0.52 | 2s | 291.51 | Talavera-Mendoza et al. (2013) |
| P0204-20 | meta-sedimentary | 400.4 | 15.26 | 2s | 291.51 | Talavera-Mendoza et al. (2013) |
| P0204-21 | meta-sedimentary | 159.0 | 9.15 | 2s | 291.51 | Talavera-Mendoza et al. (2013) |
| P0204-24 | meta-sedimentary | 575.5 | 9.30 | 2s | 291.51 | Talavera-Mendoza et al. (2013) |
| P0204-23 | meta-sedimentary | 858.5 | 10.93 | 2s | 291.51 | Talavera-Mendoza et al. (2013) |
| P0204-25 | meta-sedimentary | 439.9 | 7.31 | 2s | 291.51 | Talavera-Mendoza et al. (2013) |
| P0204-26 | meta-sedimentary | 343.9 | 24.18 | 2s | 291.51 | Talavera-Mendoza et al. (2013) |
| P0204-28 | meta-sedimentary | 201.4 | 17.58 | 2s | 291.51 | Talavera-Mendoza et al. (2013) |
| P0204-29 | meta-sedimentary | 137.7 | 6.35 | 2s | 291.51 | Talavera-Mendoza et al. (2013) |
| P0204-30 | meta-sedimentary | 204.7 | 6.03 | 2s | 291.51 | Talavera-Mendoza et al. (2013) |
| P0204-31 | meta-sedimentary | 298.6 | 26.76 | 2s | 291.51 | Talavera-Mendoza et al. (2013) |
| P0204-37 | meta-sedimentary | 462.8 | 22.10 | 2s | 291.51 | Talavera-Mendoza et al. (2013) |
| P0204-38 | meta-sedimentary | 1478.0 | 90.36 | 2s | 291.51 | Talavera-Mendoza et al. (2013) |
| P0204-39 | meta-sedimentary | 2757.7 | 55.86 | 2s | 291.51 | Talavera-Mendoza et al. (2013) |
| PO204-101 | meta-sedimentary | 59.8 | 0.59 | 2s | 291.51 | Talavera-Mendoza et al. (2013) |
| PO204-102 | meta-sedimentary | 57.7 | 0.94 | 2s | 291.51 | Talavera-Mendoza et al. (2013) |
| PO204-103 | meta-sedimentary | 59.0 | 1.20 | 2s | 291.51 | Talavera-Mendoza et al. (2013) |
| PO204-104 | meta-sedimentary | 58.9 | 0.59 | 2s | 291.51 | Talavera-Mendoza et al. (2013) |
| PO204-105 | meta-sedimentary | 59.1 | 0.59 | 2s | 291.51 | Talavera-Mendoza et al. (2013) |
| PO204-107 | meta-sedimentary | 51.2 | 0.97 | 2s | 291.51 | Talavera-Mendoza et al. (2013) |
| PO204-109 | meta-sedimentary | 994.6 | 70.36 | 2s | 291.51 | Talavera-Mendoza et al. (2013) |
| PO204-110 | meta-sedimentary | 477.7 | 35.36 | 2s | 291.51 | Talavera-Mendoza et al. (2013) |
| PO204-111 | meta-sedimentary | 533.0 | 5.83 | 2s | 291.51 | Talavera-Mendoza et al. (2013) |
| PO204-113 | meta-sedimentary | 1437.2 | 20.18 | 2s | 291.51 | Talavera-Mendoza et al. (2013) |
| PO204-114 | meta-sedimentary | 34.7 | 0.57 | 2s | 291.51 | Talavera-Mendoza et al. (2013) |
| PO204-116 | meta-sedimentary | 402.8 | 0.00 | 2s | 291.51 | Talavera-Mendoza et al. (2013) |
| PO204-118 | meta-sedimentary | 336.5 | 14.33 | 2s | 291.51 | Talavera-Mendoza et al. (2013) |
| PO204-119 | meta-sedimentary | 321.0 | 10.96 | 2s | 291.51 | Talavera-Mendoza et al. (2013) |
| PO204-120 | meta-sedimentary | 553.1 | 5.57 | 2s | 291.51 | Talavera-Mendoza et al. (2013) |
| PO204-122 | meta-sedimentary | 505.7 | 19.89 | 2s | 291.51 | Talavera-Mendoza et al. (2013) |
| PO204-123 | meta-sedimentary | 467.0 | 18.97 | 2s | 291.51 | Talavera-Mendoza et al. (2013) |
| PO204-125 | meta-sedimentary | 442.4 | 7.91 | 2s | 291.51 | Talavera-Mendoza et al. (2013) |
| PO204-126 | meta-sedimentary | 476.0 | 7.20 | 2s | 291.51 | Talavera-Mendoza et al. (2013) |
| PO204-128 | meta-sedimentary | 591.3 | 7.18 | 2s | 291.51 | Talavera-Mendoza et al. (2013) |
| PO204-129 | meta-sedimentary | 58.9 | 1.28 | 2s | 291.51 | Talavera-Mendoza et al. (2013) |
| PO204-130 | meta-sedimentary | 598.4 | 32.86 | 2s | 291.51 | Talavera-Mendoza et al. (2013) |
| PO204-132 | meta-sedimentary | 245.9 | 11.48 | 2s | 291.51 | Talavera-Mendoza et al. (2013) |
| PO204-133 | meta-sedimentary | 434.2 | 8.44 | 2s | 291.51 | Talavera-Mendoza et al. (2013) |
| PO204-135 | meta-sedimentary | 274.9 | 5.25 | 2s | 291.51 | Talavera-Mendoza et al. (2013) |
| PO204-137 | meta-sedimentary | 560.2 | 5.37 | 2s | 291.51 | Talavera-Mendoza et al. (2013) |
| PO204-138 | meta-sedimentary | 477.8 | 11.33 | 2s | 291.51 | Talavera-Mendoza et al. (2013) |
| PO204-139 | meta-sedimentary | 50.8 | 0.90 | 2s | 291.51 | Talavera-Mendoza et al. (2013) |
| PO204-140 | meta-sedimentary | 47.6 | 1.10 | 2s | 291.51 | Talavera-Mendoza et al. (2013) |
| PO204-141 | meta-sedimentary | 46.7 | 0.81 | 2s | 291.51 | Talavera-Mendoza et al. (2013) |
| PO204-147 | meta-sedimentary | 43.8 | 1.11 | 2s | 291.51 | Talavera-Mendoza et al. (2013) |
| PO204-148 | meta-sedimentary | 315.4 | 16.62 | 2s | 291.51 | Talavera-Mendoza et al. (2013) |
| PO204-149 | meta-sedimentary | 429.0 | 10.38 | 2s | 291.51 | Talavera-Mendoza et al. (2013) |
| PO204-153 | meta-sedimentary | 82.7 | 0.82 | 2s | 291.51 | Talavera-Mendoza et al. (2013) |
| PO204-154 | meta-sedimentary | 123.9 | 3.12 | 2s | 291.51 | Talavera-Mendoza et al. (2013) |
| PO204-155 | meta-sedimentary | 320.0 | 20.29 | 2s | 291.51 | Talavera-Mendoza et al. (2013) |
| PO204-156 | meta-sedimentary | 1045.8 | 49.50 | 2s | 291.51 | Talavera-Mendoza et al. (2013) |
| PO204-158 | meta-sedimentary | 669.4 | 11.13 | 2s | 291.51 | Talavera-Mendoza et al. (2013) |
| PO204-160 | meta-sedimentary | 246.6 | 18.87 | 2s | 291.51 | Talavera-Mendoza et al. (2013) |
| PO204-161 | meta-sedimentary | 188.5 | 7.34 | 2s | 291.51 | Talavera-Mendoza et al. (2013) |
| PO204-162 | meta-sedimentary | 502.2 | 22.71 | 2s | 291.51 | Talavera-Mendoza et al. (2013) |
| PO204-163 | meta-sedimentary | 461.0 | 7.47 | 2s | 291.51 | Talavera-Mendoza et al. (2013) |
| PO204-164 | meta-sedimentary | 82.8 | 3.22 | 2s | 291.51 | Talavera-Mendoza et al. (2013) |
| PO204-165 | meta-sedimentary | 42.8 | 0.43 | 2s | 291.51 | Talavera-Mendoza et al. (2013) |
| PO204-166 | meta-sedimentary | 922.8 | 22.01 | 2s | 291.51 | Talavera-Mendoza et al. (2013) |
| PO204-167 | meta-sedimentary | 489.0 | 5.09 | 2s | 291.51 | Talavera-Mendoza et al. (2013) |
| PO204-168 | meta-sedimentary | 1031.9 | 33.73 | 2s | 291.51 | Talavera-Mendoza et al. (2013) |
| PO204-170 | meta-sedimentary | 333.9 | 3.74 | 2s | 291.51 | Talavera-Mendoza et al. (2013) |
| PO204-171 | meta-sedimentary | 273.5 | 16.95 | 2s | 291.51 | Talavera-Mendoza et al. (2013) |
| PO204-172 | meta-sedimentary | 524.6 | 14.86 | 2s | 291.51 | Talavera-Mendoza et al. (2013) |
| PO204-173 | meta-sedimentary | 426.7 | 12.76 | 2s | 291.51 | Talavera-Mendoza et al. (2013) |
| PO204-174 | meta-sedimentary | 2218.1 | 25.17 | 2s | 291.51 | Talavera-Mendoza et al. (2013) |
| PO204-175 | meta-sedimentary | 2311.9 | 29.37 | 2s | 291.51 | Talavera-Mendoza et al. (2013) |
| PO204-176 | meta-sedimentary | 61.4 | 0.61 | 2s | 291.51 | Talavera-Mendoza et al. (2013) |
| PO204-177 | meta-sedimentary | 59.5 | 1.69 | 2s | 291.51 | Talavera-Mendoza et al. (2013) |
| PO204-178 | meta-sedimentary | 554.5 | 12.59 | 2s | 291.51 | Talavera-Mendoza et al. (2013) |
| PO204-179 | meta-sedimentary | 62.2 | 0.62 | 2s | 291.51 | Talavera-Mendoza et al. (2013) |
| PO204-183 | meta-sedimentary | 243.5 | 9.51 | 2s | 291.51 | Talavera-Mendoza et al. (2013) |
| PO204-185 | meta-sedimentary | 38.6 | 1.25 | 2s | 291.51 | Talavera-Mendoza et al. (2013) |
| PO204-186 | meta-sedimentary | 35.9 | 0.56 | 2s | 291.51 | Talavera-Mendoza et al. (2013) |
| PO204-187 | meta-sedimentary | 36.5 | 0.50 | 2s | 291.51 | Talavera-Mendoza et al. (2013) |
| PO204-188 | meta-sedimentary | 1076.5 | 105.40 | 2s | 291.51 | Talavera-Mendoza et al. (2013) |
| PO204-189 | meta-sedimentary | 285.2 | 22.01 | 2s | 291.51 | Talavera-Mendoza et al. (2013) |
| PO204-190 | meta-sedimentary | 223.0 | 12.41 | 2s | 291.51 | Talavera-Mendoza et al. (2013) |
| PO204-191 | meta-sedimentary | 195.4 | 16.46 | 2s | 291.51 | Talavera-Mendoza et al. (2013) |
| PO204-192 | meta-sedimentary | 68.1 | 1.05 | 2s | 291.51 | Talavera-Mendoza et al. (2013) |
| PO204-193 | meta-sedimentary | 422.8 | 8.19 | 2s | 291.51 | Talavera-Mendoza et al. (2013) |
| PO204-194 | meta-sedimentary | 451.3 | 22.71 | 2s | 291.51 | Talavera-Mendoza et al. (2013) |
| PO204-195 | meta-sedimentary | 34.9 | 0.35 | 2s | 291.51 | Talavera-Mendoza et al. (2013) |
| PO204-197 | meta-sedimentary | 334.2 | 7.23 | 2s | 291.51 | Talavera-Mendoza et al. (2013) |
| PO204-199 | meta-sedimentary | 107.9 | 4.51 | 2s | 291.51 | Talavera-Mendoza et al. (2013) |
| PO204-200 | meta-sedimentary | 515.2 | 13.86 | 2s | 291.51 | Talavera-Mendoza et al. (2013) |
| PO211-1 | meta-sedimentary | 256.6 | 3.09 | 2s | 440.19 | Talavera-Mendoza et al. (2013) |
| PO211-2 | meta-sedimentary | 339.3 | 12.40 | 2s | 440.19 | Talavera-Mendoza et al. (2013) |
| PO211-3 | meta-sedimentary | 34.8 | 0.78 | 2s | 440.19 | Talavera-Mendoza et al. (2013) |
| PO211-4 | meta-sedimentary | 274.4 | 6.02 | 2s | 440.19 | Talavera-Mendoza et al. (2013) |
| PO211-5 | meta-sedimentary | 1092.3 | 35.38 | 2s | 440.19 | Talavera-Mendoza et al. (2013) |
| PO211-7 | meta-sedimentary | 593.2 | 14.79 | 2s | 440.19 | Talavera-Mendoza et al. (2013) |
| PO211-8 | meta-sedimentary | 844.1 | 53.09 | 2s | 440.19 | Talavera-Mendoza et al. (2013) |
| PO211-9 | meta-sedimentary | 594.9 | 9.38 | 2s | 440.19 | Talavera-Mendoza et al. (2013) |
| PO211-6 | meta-sedimentary | 293.6 | 13.95 | 2s | 440.19 | Talavera-Mendoza et al. (2013) |
| PO211-10 | meta-sedimentary | 471.1 | 36.89 | 2s | 440.19 | Talavera-Mendoza et al. (2013) |
| PO211-11 | meta-sedimentary | 45.0 | 3.25 | 2s | 440.19 | Talavera-Mendoza et al. (2013) |
| PO211-13 | meta-sedimentary | 213.4 | 3.44 | 2s | 440.19 | Talavera-Mendoza et al. (2013) |
| PO211-14 | meta-sedimentary | 184.0 | 1.81 | 2s | 440.19 | Talavera-Mendoza et al. (2013) |
| PO211-15 | meta-sedimentary | 222.0 | 4.43 | 2s | 440.19 | Talavera-Mendoza et al. (2013) |
| PO211-17 | meta-sedimentary | 227.2 | 11.14 | 2s | 440.19 | Talavera-Mendoza et al. (2013) |
| PO211-18 | meta-sedimentary | 531.2 | 5.10 | 2s | 440.19 | Talavera-Mendoza et al. (2013) |
| PO211-20 | meta-sedimentary | 283.0 | 9.50 | 2s | 440.19 | Talavera-Mendoza et al. (2013) |
| PO211-19 | meta-sedimentary | 843.5 | 9.49 | 2s | 440.19 | Talavera-Mendoza et al. (2013) |
| PO211-21 | meta-sedimentary | 223.3 | 3.82 | 2s | 440.19 | Talavera-Mendoza et al. (2013) |
| PO211-22 | meta-sedimentary | 501.8 | 12.22 | 2s | 440.19 | Talavera-Mendoza et al. (2013) |
| PO211-23 | meta-sedimentary | 212.6 | 11.63 | 2s | 440.19 | Talavera-Mendoza et al. (2013) |
| PO211-27 | meta-sedimentary | 301.2 | 7.65 | 2s | 440.19 | Talavera-Mendoza et al. (2013) |
| PO211-28 | meta-sedimentary | 32.3 | 0.96 | 2s | 440.19 | Talavera-Mendoza et al. (2013) |
| PO211-29 | meta-sedimentary | 34.1 | 0.34 | 2s | 440.19 | Talavera-Mendoza et al. (2013) |
| PO211-30 | meta-sedimentary | 31.3 | 1.58 | 2s | 440.19 | Talavera-Mendoza et al. (2013) |
| PO211-31 | meta-sedimentary | 286.6 | 5.49 | 2s | 440.19 | Talavera-Mendoza et al. (2013) |
| PO211-34 | meta-sedimentary | 253.5 | 4.82 | 2s | 440.19 | Talavera-Mendoza et al. (2013) |
| PO211-35 | meta-sedimentary | 282.6 | 7.02 | 2s | 440.19 | Talavera-Mendoza et al. (2013) |
| PO211-36 | meta-sedimentary | 35.3 | 2.05 | 2s | 440.19 | Talavera-Mendoza et al. (2013) |
| PO211-38 | meta-sedimentary | 840.4 | 31.36 | 2s | 440.19 | Talavera-Mendoza et al. (2013) |
| PO211-39 | meta-sedimentary | 199.2 | 3.37 | 2s | 440.19 | Talavera-Mendoza et al. (2013) |
| PO211-40 | meta-sedimentary | 240.9 | 13.34 | 2s | 440.19 | Talavera-Mendoza et al. (2013) |
| PO211-41 | meta-sedimentary | 33.4 | 0.56 | 2s | 440.19 | Talavera-Mendoza et al. (2013) |
| PO211-42 | meta-sedimentary | 29.9 | 1.31 | 2s | 440.19 | Talavera-Mendoza et al. (2013) |
| PO211-44 | meta-sedimentary | 866.2 | 33.80 | 2s | 440.19 | Talavera-Mendoza et al. (2013) |
| PO211-45 | meta-sedimentary | 262.7 | 4.86 | 2s | 440.19 | Talavera-Mendoza et al. (2013) |
| PO211-46 | meta-sedimentary | 463.4 | 6.93 | 2s | 440.19 | Talavera-Mendoza et al. (2013) |
| PO211-47 | meta-sedimentary | 531.4 | 20.35 | 2s | 440.19 | Talavera-Mendoza et al. (2013) |
| PO211-48 | meta-sedimentary | 1799.2 | 21.69 | 2s | 440.19 | Talavera-Mendoza et al. (2013) |
| PO211-49 | meta-sedimentary | 394.1 | 16.74 | 2s | 440.19 | Talavera-Mendoza et al. (2013) |
| PO211-52 | meta-sedimentary | 271.4 | 2.66 | 2s | 440.19 | Talavera-Mendoza et al. (2013) |
| PO211-53 | meta-sedimentary | 209.3 | 17.01 | 2s | 440.19 | Talavera-Mendoza et al. (2013) |
| PO211-54 | meta-sedimentary | 210.8 | 4.67 | 2s | 440.19 | Talavera-Mendoza et al. (2013) |
| XO-73-1 | meta-sedimentary | 73.1 | 4.43 | 2s | 450.00 | Talavera-Mendoza et al. (2013) |
| XO-73-2 | meta-sedimentary | 61.4 | 3.34 | 2s | 450.00 | Talavera-Mendoza et al. (2013) |
| XO-73-3 | meta-sedimentary | 61.9 | 1.27 | 2s | 450.00 | Talavera-Mendoza et al. (2013) |
| XO-73-4 | meta-sedimentary | 66.4 | 2.50 | 2s | 450.00 | Talavera-Mendoza et al. (2013) |
| XO-73-5 | meta-sedimentary | 66.1 | 2.70 | 2s | 450.00 | Talavera-Mendoza et al. (2013) |
| XO-73-6 | meta-sedimentary | 68.5 | 4.56 | 2s | 450.00 | Talavera-Mendoza et al. (2013) |
| XO-73-7 | meta-sedimentary | 44.5 | 1.64 | 2s | 450.00 | Talavera-Mendoza et al. (2013) |
| XO-73-8 | meta-sedimentary | 57.9 | 3.18 | 2s | 450.00 | Talavera-Mendoza et al. (2013) |
| XO-73-9 | meta-sedimentary | 72.1 | 4.54 | 2s | 450.00 | Talavera-Mendoza et al. (2013) |
| XO-73-10 | meta-sedimentary | 169.1 | 5.12 | 2s | 450.00 | Talavera-Mendoza et al. (2013) |
| XO-73-11 | meta-sedimentary | 522.6 | 10.49 | 2s | 450.00 | Talavera-Mendoza et al. (2013) |
| XO-73-13 | meta-sedimentary | 56.5 | 3.60 | 2s | 450.00 | Talavera-Mendoza et al. (2013) |
| XO-73-14 | meta-sedimentary | 331.9 | 20.12 | 2s | 450.00 | Talavera-Mendoza et al. (2013) |
| XO-73-15 | meta-sedimentary | 64.5 | 5.17 | 2s | 450.00 | Talavera-Mendoza et al. (2013) |
| XO-73-16 | meta-sedimentary | 61.6 | 1.15 | 2s | 450.00 | Talavera-Mendoza et al. (2013) |
| XO-73-17 | meta-sedimentary | 54.1 | 2.48 | 2s | 450.00 | Talavera-Mendoza et al. (2013) |
| XO-73-18 | meta-sedimentary | 250.6 | 7.69 | 2s | 450.00 | Talavera-Mendoza et al. (2013) |
| XO-73-19 | meta-sedimentary | 328.6 | 21.59 | 2s | 450.00 | Talavera-Mendoza et al. (2013) |
| XO-73-20 | meta-sedimentary | 457.7 | 25.98 | 2s | 450.00 | Talavera-Mendoza et al. (2013) |
| XO-73-21 | meta-sedimentary | 59.8 | 1.48 | 2s | 450.00 | Talavera-Mendoza et al. (2013) |
| XO-73-22 | meta-sedimentary | 540.5 | 44.38 | 2s | 450.00 | Talavera-Mendoza et al. (2013) |
| XO-73-23 | meta-sedimentary | 549.4 | 23.96 | 2s | 450.00 | Talavera-Mendoza et al. (2013) |
| XO-73-25 | meta-sedimentary | 1133.5 | 90.55 | 2s | 450.00 | Talavera-Mendoza et al. (2013) |
| XO-73-26 | meta-sedimentary | 529.5 | 11.03 | 2s | 450.00 | Talavera-Mendoza et al. (2013) |
| XO-73-28 | meta-sedimentary | 526.6 | 22.05 | 2s | 450.00 | Talavera-Mendoza et al. (2013) |
| XO-73-29 | meta-sedimentary | 228.1 | 4.17 | 2s | 450.00 | Talavera-Mendoza et al. (2013) |
| XO-73-30 | meta-sedimentary | 58.4 | 1.47 | 2s | 450.00 | Talavera-Mendoza et al. (2013) |
| XO-73-34 | meta-sedimentary | 72.9 | 3.04 | 2s | 450.00 | Talavera-Mendoza et al. (2013) |
| XO-73-35 | meta-sedimentary | 358.6 | 16.29 | 2s | 450.00 | Talavera-Mendoza et al. (2013) |
| XO-73-36 | meta-sedimentary | 255.8 | 3.81 | 2s | 450.00 | Talavera-Mendoza et al. (2013) |
| XO-73-37 | meta-sedimentary | 565.9 | 28.82 | 2s | 450.00 | Talavera-Mendoza et al. (2013) |
| XO-73-39 | meta-sedimentary | 1063.2 | 88.84 | 2s | 450.00 | Talavera-Mendoza et al. (2013) |
| XO-73-40 | meta-sedimentary | 224.5 | 12.44 | 2s | 450.00 | Talavera-Mendoza et al. (2013) |
| XO-73-43 | meta-sedimentary | 64.6 | 1.70 | 2s | 450.00 | Talavera-Mendoza et al. (2013) |
| XO-73-44 | meta-sedimentary | 59.3 | 5.84 | 2s | 450.00 | Talavera-Mendoza et al. (2013) |
| XO-73-45 | meta-sedimentary | 462.6 | 41.43 | 2s | 450.00 | Talavera-Mendoza et al. (2013) |
| XO-73-46 | meta-sedimentary | 55.8 | 2.55 | 2s | 450.00 | Talavera-Mendoza et al. (2013) |
| XO-73-47 | meta-sedimentary | 63.9 | 2.38 | 2s | 450.00 | Talavera-Mendoza et al. (2013) |
| XO-73-48 | meta-sedimentary | 397.7 | 21.21 | 2s | 450.00 | Talavera-Mendoza et al. (2013) |
| XO-73-50 | meta-sedimentary | 66.0 | 2.64 | 2s | 450.00 | Talavera-Mendoza et al. (2013) |
| XO-73-51 | meta-sedimentary | 67.9 | 3.23 | 2s | 450.00 | Talavera-Mendoza et al. (2013) |
| XO-73-52 | meta-sedimentary | 65.3 | 2.51 | 2s | 450.00 | Talavera-Mendoza et al. (2013) |
| XO-73-53 | meta-sedimentary | 290.0 | 21.13 | 2s | 450.00 | Talavera-Mendoza et al. (2013) |
| XO-73-54 | meta-sedimentary | 66.6 | 5.31 | 2s | 450.00 | Talavera-Mendoza et al. (2013) |
| XO-73-55 | meta-sedimentary | 403.6 | 35.87 | 2s | 450.00 | Talavera-Mendoza et al. (2013) |
| XO-73-56 | meta-sedimentary | 66.0 | 5.45 | 2s | 450.00 | Talavera-Mendoza et al. (2013) |
| XO-73-58 | meta-sedimentary | 482.3 | 13.52 | 2s | 450.00 | Talavera-Mendoza et al. (2013) |
| XO-73-59 | meta-sedimentary | 59.3 | 1.85 | 2s | 450.00 | Talavera-Mendoza et al. (2013) |
| XO-73-60 | meta-sedimentary | 458.5 | 11.30 | 2s | 450.00 | Talavera-Mendoza et al. (2013) |
| XO-73-61 | meta-sedimentary | 66.2 | 1.49 | 2s | 450.00 | Talavera-Mendoza et al. (2013) |
| XO-73-62 | meta-sedimentary | 557.6 | 13.03 | 2s | 450.00 | Talavera-Mendoza et al. (2013) |
| XO-73-63 | meta-sedimentary | 65.1 | 2.42 | 2s | 450.00 | Talavera-Mendoza et al. (2013) |
| XO-73-64 | meta-sedimentary | 64.1 | 4.43 | 2s | 450.00 | Talavera-Mendoza et al. (2013) |
| XO-73-66 | meta-sedimentary | 65.0 | 3.56 | 2s | 450.00 | Talavera-Mendoza et al. (2013) |
| XO-73-69 | meta-sedimentary | 480.0 | 27.57 | 2s | 450.00 | Talavera-Mendoza et al. (2013) |
| XO-73-70 | meta-sedimentary | 64.1 | 2.00 | 2s | 450.00 | Talavera-Mendoza et al. (2013) |
| XO-73-73 | meta-sedimentary | 577.9 | 32.17 | 2s | 450.00 | Talavera-Mendoza et al. (2013) |
| XO-73-74 | meta-sedimentary | 451.8 | 9.36 | 2s | 450.00 | Talavera-Mendoza et al. (2013) |
| XO-73-76 | meta-sedimentary | 70.8 | 1.29 | 2s | 450.00 | Talavera-Mendoza et al. (2013) |
| XO-73-77 | meta-sedimentary | 530.3 | 13.50 | 2s | 450.00 | Talavera-Mendoza et al. (2013) |
| XO-73-78 | meta-sedimentary | 445.8 | 9.35 | 2s | 450.00 | Talavera-Mendoza et al. (2013) |
| XO-73-79 | meta-sedimentary | 365.7 | 4.52 | 2s | 450.00 | Talavera-Mendoza et al. (2013) |
| XO-73-81 | meta-sedimentary | 37.2 | 3.63 | 2s | 450.00 | Talavera-Mendoza et al. (2013) |
| XO-73-83 | meta-sedimentary | 69.1 | 4.40 | 2s | 450.00 | Talavera-Mendoza et al. (2013) |
| XO-73-84 | meta-sedimentary | 42.8 | 3.87 | 2s | 450.00 | Talavera-Mendoza et al. (2013) |
| XO-73-85 | meta-sedimentary | 536.8 | 14.32 | 2s | 450.00 | Talavera-Mendoza et al. (2013) |
| XO-73-86 | meta-sedimentary | 313.1 | 25.79 | 2s | 450.00 | Talavera-Mendoza et al. (2013) |
| XO-73-87 | meta-sedimentary | 527.8 | 15.05 | 2s | 450.00 | Talavera-Mendoza et al. (2013) |
| XO-73-90 | meta-sedimentary | 53.5 | 0.84 | 2s | 450.00 | Talavera-Mendoza et al. (2013) |
| XO-73-92 | meta-sedimentary | 415.2 | 27.70 | 2s | 450.00 | Talavera-Mendoza et al. (2013) |
| XO-73-93 | meta-sedimentary | 1480.8 | 72.51 | 2s | 450.00 | Talavera-Mendoza et al. (2013) |
| XO-73-94 | meta-sedimentary | 67.0 | 2.26 | 2s | 450.00 | Talavera-Mendoza et al. (2013) |
| XO-73-95 | meta-sedimentary | 320.5 | 16.54 | 2s | 450.00 | Talavera-Mendoza et al. (2013) |
| XO-73-97 | meta-sedimentary | 138.6 | 2.48 | 2s | 450.00 | Talavera-Mendoza et al. (2013) |
| XO-73-98 | meta-sedimentary | 540.1 | 16.48 | 2s | 450.00 | Talavera-Mendoza et al. (2013) |
| XO-73-102 | meta-sedimentary | 514.4 | 14.93 | 2s | 450.00 | Talavera-Mendoza et al. (2013) |
| XO-73-103 | meta-sedimentary | 272.3 | 23.75 | 2s | 450.00 | Talavera-Mendoza et al. (2013) |
| XO-73-104 | meta-sedimentary | 1743.3 | 47.37 | 2s | 450.00 | Talavera-Mendoza et al. (2013) |
| XO-73-106 | meta-sedimentary | 409.7 | 27.11 | 2s | 450.00 | Talavera-Mendoza et al. (2013) |
| PO52-1 | meta-sedimentary | 180.1 | 1.92 | 2s | 88.40 | Talavera-Mendoza et al. (2013) |
| PO52-2 | meta-sedimentary | 180.2 | 1.95 | 2s | 88.40 | Talavera-Mendoza et al. (2013) |
| PO52-3 | meta-sedimentary | 174.5 | 2.53 | 2s | 88.40 | Talavera-Mendoza et al. (2013) |
| PO52-4 | meta-sedimentary | 175.0 | 6.18 | 2s | 88.40 | Talavera-Mendoza et al. (2013) |
| PO52-5 | meta-sedimentary | 174.6 | 5.31 | 2s | 88.40 | Talavera-Mendoza et al. (2013) |
| PO52-6 | meta-sedimentary | 173.8 | 1.71 | 2s | 88.40 | Talavera-Mendoza et al. (2013) |
| PO52-7 | meta-sedimentary | 177.5 | 1.75 | 2s | 88.40 | Talavera-Mendoza et al. (2013) |
| PO52-8 | meta-sedimentary | 175.5 | 1.73 | 2s | 88.40 | Talavera-Mendoza et al. (2013) |
| PO52-9 | meta-sedimentary | 172.5 | 1.70 | 2s | 88.40 | Talavera-Mendoza et al. (2013) |
| PO52-10 | meta-sedimentary | 182.7 | 1.80 | 2s | 88.40 | Talavera-Mendoza et al. (2013) |
| PO52-11 | meta-sedimentary | 174.4 | 1.72 | 2s | 88.40 | Talavera-Mendoza et al. (2013) |
| PO52-12 | meta-sedimentary | 173.7 | 1.71 | 2s | 88.40 | Talavera-Mendoza et al. (2013) |
| PO52-13 | meta-sedimentary | 165.3 | 1.80 | 2s | 88.40 | Talavera-Mendoza et al. (2013) |
| PO52-14 | meta-sedimentary | 170.6 | 4.50 | 2s | 88.40 | Talavera-Mendoza et al. (2013) |
| PO52-16 | meta-sedimentary | 180.9 | 3.09 | 2s | 88.40 | Talavera-Mendoza et al. (2013) |
| PO52-17 | meta-sedimentary | 170.4 | 2.22 | 2s | 88.40 | Talavera-Mendoza et al. (2013) |
| PO52-19 | meta-sedimentary | 174.3 | 2.96 | 2s | 88.40 | Talavera-Mendoza et al. (2013) |
| PO52-20 | meta-sedimentary | 160.7 | 1.59 | 2s | 88.40 | Talavera-Mendoza et al. (2013) |
| PO52-22 | meta-sedimentary | 161.8 | 3.19 | 2s | 88.40 | Talavera-Mendoza et al. (2013) |
| PO52-23 | meta-sedimentary | 191.8 | 1.89 | 2s | 88.40 | Talavera-Mendoza et al. (2013) |
| PO52-24 | meta-sedimentary | 179.1 | 2.01 | 2s | 88.40 | Talavera-Mendoza et al. (2013) |
| PO52-25 | meta-sedimentary | 172.1 | 2.92 | 2s | 88.40 | Talavera-Mendoza et al. (2013) |
| PO52-26 | meta-sedimentary | 150.8 | 3.13 | 2s | 88.40 | Talavera-Mendoza et al. (2013) |
| PO52-27 | meta-sedimentary | 166.0 | 1.64 | 2s | 88.40 | Talavera-Mendoza et al. (2013) |
| PO52-29 | meta-sedimentary | 160.0 | 2.16 | 2s | 88.40 | Talavera-Mendoza et al. (2013) |
| PO52-30 | meta-sedimentary | 162.6 | 1.86 | 2s | 88.40 | Talavera-Mendoza et al. (2013) |
| PO52-32 | meta-sedimentary | 171.6 | 1.69 | 2s | 88.40 | Talavera-Mendoza et al. (2013) |
| PO52-33 | meta-sedimentary | 175.7 | 1.73 | 2s | 88.40 | Talavera-Mendoza et al. (2013) |
| PO52-34 | meta-sedimentary | 179.1 | 1.77 | 2s | 88.40 | Talavera-Mendoza et al. (2013) |
| PO52-35 | meta-sedimentary | 172.0 | 1.70 | 2s | 88.40 | Talavera-Mendoza et al. (2013) |
| PO52-36 | meta-sedimentary | 177.7 | 1.75 | 2s | 88.40 | Talavera-Mendoza et al. (2013) |
| PO52-38 | meta-sedimentary | 151.3 | 3.54 | 2s | 88.40 | Talavera-Mendoza et al. (2013) |
| PO52-39 | meta-sedimentary | 171.6 | 2.20 | 2s | 88.40 | Talavera-Mendoza et al. (2013) |
| PO52-40 | meta-sedimentary | 219.2 | 2.15 | 2s | 88.40 | Talavera-Mendoza et al. (2013) |
| PO52-41 | meta-sedimentary | 174.3 | 2.36 | 2s | 88.40 | Talavera-Mendoza et al. (2013) |
| PO52-42 | meta-sedimentary | 162.7 | 1.61 | 2s | 88.40 | Talavera-Mendoza et al. (2013) |
| PO52-43 | meta-sedimentary | 1159.3 | 30.54 | 2s | 88.40 | Talavera-Mendoza et al. (2013) |
| PO52-44 | meta-sedimentary | 183.7 | 2.19 | 2s | 88.40 | Talavera-Mendoza et al. (2013) |
| PO52-45 | meta-sedimentary | 174.9 | 5.09 | 2s | 88.40 | Talavera-Mendoza et al. (2013) |
| PO52-46 | meta-sedimentary | 179.7 | 8.38 | 2s | 88.40 | Talavera-Mendoza et al. (2013) |
| PO52-47 | meta-sedimentary | 165.1 | 3.96 | 2s | 88.40 | Talavera-Mendoza et al. (2013) |
| PO52-48 | meta-sedimentary | 167.2 | 3.63 | 2s | 88.40 | Talavera-Mendoza et al. (2013) |
| PO52-49 | meta-sedimentary | 179.8 | 2.71 | 2s | 88.40 | Talavera-Mendoza et al. (2013) |
| PO52-50 | meta-sedimentary | 178.0 | 4.74 | 2s | 88.40 | Talavera-Mendoza et al. (2013) |
| PO52-51 | meta-sedimentary | 175.0 | 4.49 | 2s | 88.40 | Talavera-Mendoza et al. (2013) |
| PO52-52 | meta-sedimentary | 313.8 | 5.36 | 2s | 88.40 | Talavera-Mendoza et al. (2013) |
| PO52-53 | meta-sedimentary | 153.8 | 4.70 | 2s | 88.40 | Talavera-Mendoza et al. (2013) |
| PO52-54 | meta-sedimentary | 155.2 | 6.06 | 2s | 88.40 | Talavera-Mendoza et al. (2013) |
| PO52-55 | meta-sedimentary | 174.2 | 1.72 | 2s | 88.40 | Talavera-Mendoza et al. (2013) |
| PO52-56 | meta-sedimentary | 186.5 | 2.52 | 2s | 88.40 | Talavera-Mendoza et al. (2013) |
| PO52-57 | meta-sedimentary | 187.2 | 1.84 | 2s | 88.40 | Talavera-Mendoza et al. (2013) |
| PO52-58 | meta-sedimentary | 189.9 | 4.98 | 2s | 88.40 | Talavera-Mendoza et al. (2013) |
| PO52-59 | meta-sedimentary | 191.9 | 1.89 | 2s | 88.40 | Talavera-Mendoza et al. (2013) |
| PO52-60 | meta-sedimentary | 177.9 | 6.00 | 2s | 88.40 | Talavera-Mendoza et al. (2013) |
| PO53-1 | meta-sedimentary | 155.7 | 2.98 | 2s | 88.40 | Talavera-Mendoza et al. (2013) |
| PO53-2 | meta-sedimentary | 130.4 | 4.36 | 2s | 88.40 | Talavera-Mendoza et al. (2013) |
| PO53-4 | meta-sedimentary | 146.4 | 4.43 | 2s | 88.40 | Talavera-Mendoza et al. (2013) |
| PO53-5 | meta-sedimentary | 135.9 | 2.85 | 2s | 88.40 | Talavera-Mendoza et al. (2013) |
| PO53-6 | meta-sedimentary | 186.9 | 2.97 | 2s | 88.40 | Talavera-Mendoza et al. (2013) |
| PO53-7 | meta-sedimentary | 169.4 | 1.67 | 2s | 88.40 | Talavera-Mendoza et al. (2013) |
| PO53-8 | meta-sedimentary | 171.5 | 1.69 | 2s | 88.40 | Talavera-Mendoza et al. (2013) |
| PO53-9 | meta-sedimentary | 167.1 | 1.83 | 2s | 88.40 | Talavera-Mendoza et al. (2013) |
| PO53-10 | meta-sedimentary | 170.2 | 3.75 | 2s | 88.40 | Talavera-Mendoza et al. (2013) |
| PO53-11 | meta-sedimentary | 165.6 | 1.81 | 2s | 88.40 | Talavera-Mendoza et al. (2013) |
| PO53-12 | meta-sedimentary | 169.8 | 2.01 | 2s | 88.40 | Talavera-Mendoza et al. (2013) |
| PO53-13 | meta-sedimentary | 160.8 | 2.22 | 2s | 88.40 | Talavera-Mendoza et al. (2013) |
| PO53-14 | meta-sedimentary | 121.2 | 4.36 | 2s | 88.40 | Talavera-Mendoza et al. (2013) |
| PO53-15 | meta-sedimentary | 169.2 | 2.20 | 2s | 88.40 | Talavera-Mendoza et al. (2013) |
| PO53-16 | meta-sedimentary | 111.4 | 1.10 | 2s | 88.40 | Talavera-Mendoza et al. (2013) |
| PO53-17 | meta-sedimentary | 168.4 | 1.75 | 2s | 88.40 | Talavera-Mendoza et al. (2013) |
| PO53-18 | meta-sedimentary | 134.3 | 2.83 | 2s | 88.40 | Talavera-Mendoza et al. (2013) |
| PO53-19 | meta-sedimentary | 162.4 | 2.76 | 2s | 88.40 | Talavera-Mendoza et al. (2013) |
| PO53-20 | meta-sedimentary | 139.1 | 1.38 | 2s | 88.40 | Talavera-Mendoza et al. (2013) |
| PO53-21 | meta-sedimentary | 150.0 | 1.48 | 2s | 88.40 | Talavera-Mendoza et al. (2013) |
| PO53-22 | meta-sedimentary | 153.5 | 4.23 | 2s | 88.40 | Talavera-Mendoza et al. (2013) |
| PO53-23 | meta-sedimentary | 171.7 | 2.59 | 2s | 88.40 | Talavera-Mendoza et al. (2013) |
| PO53-24 | meta-sedimentary | 139.3 | 1.38 | 2s | 88.40 | Talavera-Mendoza et al. (2013) |
| PO53-25 | meta-sedimentary | 171.4 | 1.69 | 2s | 88.40 | Talavera-Mendoza et al. (2013) |
| PO53-26 | meta-sedimentary | 169.4 | 5.02 | 2s | 88.40 | Talavera-Mendoza et al. (2013) |
| PO53-27 | meta-sedimentary | 170.8 | 1.82 | 2s | 88.40 | Talavera-Mendoza et al. (2013) |
| PO53-28 | meta-sedimentary | 175.0 | 1.73 | 2s | 88.40 | Talavera-Mendoza et al. (2013) |
| PO53-29 | meta-sedimentary | 174.9 | 3.42 | 2s | 88.40 | Talavera-Mendoza et al. (2013) |
| PO53-30 | meta-sedimentary | 173.3 | 1.88 | 2s | 88.40 | Talavera-Mendoza et al. (2013) |
| PO53-31 | meta-sedimentary | 171.8 | 1.70 | 2s | 88.40 | Talavera-Mendoza et al. (2013) |
| PO53-32 | meta-sedimentary | 167.4 | 2.43 | 2s | 88.40 | Talavera-Mendoza et al. (2013) |
| PO53-33 | meta-sedimentary | 170.0 | 2.05 | 2s | 88.40 | Talavera-Mendoza et al. (2013) |
| PO53-34 | meta-sedimentary | 171.7 | 3.40 | 2s | 88.40 | Talavera-Mendoza et al. (2013) |
| PO53-35 | meta-sedimentary | 170.0 | 2.72 | 2s | 88.40 | Talavera-Mendoza et al. (2013) |
| PO53-36 | meta-sedimentary | 177.9 | 1.75 | 2s | 88.40 | Talavera-Mendoza et al. (2013) |
| PO53-37 | meta-sedimentary | 154.0 | 1.52 | 2s | 88.40 | Talavera-Mendoza et al. (2013) |
| PO53-38 | meta-sedimentary | 150.2 | 4.59 | 2s | 88.40 | Talavera-Mendoza et al. (2013) |
| PO53-39 | meta-sedimentary | 164.7 | 2.10 | 2s | 88.40 | Talavera-Mendoza et al. (2013) |
| PO53-40 | meta-sedimentary | 143.1 | 1.42 | 2s | 88.40 | Talavera-Mendoza et al. (2013) |
| PO53-41 | meta-sedimentary | 166.3 | 1.64 | 2s | 88.40 | Talavera-Mendoza et al. (2013) |
| PO53-42 | meta-sedimentary | 160.9 | 1.59 | 2s | 88.40 | Talavera-Mendoza et al. (2013) |
| PO53-43 | meta-sedimentary | 166.5 | 1.64 | 2s | 88.40 | Talavera-Mendoza et al. (2013) |
| PO53-44 | meta-sedimentary | 164.7 | 1.63 | 2s | 88.40 | Talavera-Mendoza et al. (2013) |
| PO53-45 | meta-sedimentary | 167.2 | 2.05 | 2s | 88.40 | Talavera-Mendoza et al. (2013) |
| PO53-46 | meta-sedimentary | 160.2 | 1.58 | 2s | 88.40 | Talavera-Mendoza et al. (2013) |
| PO53-47 | meta-sedimentary | 166.1 | 2.28 | 2s | 88.40 | Talavera-Mendoza et al. (2013) |
| PO53-48 | meta-sedimentary | 159.6 | 1.58 | 2s | 88.40 | Talavera-Mendoza et al. (2013) |
| PO53-49 | meta-sedimentary | 158.5 | 1.57 | 2s | 88.40 | Talavera-Mendoza et al. (2013) |
| PO53-50 | meta-sedimentary | 165.6 | 1.63 | 2s | 88.40 | Talavera-Mendoza et al. (2013) |
| PO53-51 | meta-sedimentary | 161.4 | 3.65 | 2s | 88.40 | Talavera-Mendoza et al. (2013) |
| PO53-52 | meta-sedimentary | 142.8 | 2.57 | 2s | 88.40 | Talavera-Mendoza et al. (2013) |
| PO53-53 | meta-sedimentary | 135.7 | 2.52 | 2s | 88.40 | Talavera-Mendoza et al. (2013) |
| PO53-54 | meta-sedimentary | 168.1 | 2.41 | 2s | 88.40 | Talavera-Mendoza et al. (2013) |
| PO53-55 | meta-sedimentary | 144.7 | 1.43 | 2s | 88.40 | Talavera-Mendoza et al. (2013) |
| PO53-56 | meta-sedimentary | 159.2 | 5.03 | 2s | 88.40 | Talavera-Mendoza et al. (2013) |
| PO53-57 | meta-sedimentary | 169.9 | 1.88 | 2s | 88.40 | Talavera-Mendoza et al. (2013) |
| PO53-59 | meta-sedimentary | 176.2 | 1.74 | 2s | 88.40 | Talavera-Mendoza et al. (2013) |
| PO53-60 | meta-sedimentary | 163.4 | 2.89 | 2s | 88.40 | Talavera-Mendoza et al. (2013) |
| PO53-61 | meta-sedimentary | 163.8 | 2.83 | 2s | 88.40 | Talavera-Mendoza et al. (2013) |
| PO53-62 | meta-sedimentary | 162.4 | 1.60 | 2s | 88.40 | Talavera-Mendoza et al. (2013) |
| PO53-63 | meta-sedimentary | 172.0 | 1.70 | 2s | 88.40 | Talavera-Mendoza et al. (2013) |
| PO53-64 | meta-sedimentary | 148.1 | 2.36 | 2s | 88.40 | Talavera-Mendoza et al. (2013) |
| PO53-65 | meta-sedimentary | 172.6 | 1.70 | 2s | 88.40 | Talavera-Mendoza et al. (2013) |
| PO53-66 | meta-sedimentary | 169.7 | 1.67 | 2s | 88.40 | Talavera-Mendoza et al. (2013) |
| PO53-67 | meta-sedimentary | 151.2 | 1.76 | 2s | 88.40 | Talavera-Mendoza et al. (2013) |
| PO53-68 | meta-sedimentary | 166.1 | 2.95 | 2s | 88.40 | Talavera-Mendoza et al. (2013) |
| PO53-69 | meta-sedimentary | 165.9 | 1.64 | 2s | 88.40 | Talavera-Mendoza et al. (2013) |
| PO53-70 | meta-sedimentary | 168.0 | 1.66 | 2s | 88.40 | Talavera-Mendoza et al. (2013) |
| PO53-71 | meta-sedimentary | 147.3 | 3.23 | 2s | 88.40 | Talavera-Mendoza et al. (2013) |
| PO53-72 | meta-sedimentary | 89.7 | 4.46 | 2s | 88.40 | Talavera-Mendoza et al. (2013) |
| PO53-73 | meta-sedimentary | 161.3 | 5.97 | 2s | 88.40 | Talavera-Mendoza et al. (2013) |
| PO53-74 | meta-sedimentary | 167.3 | 1.65 | 2s | 88.40 | Talavera-Mendoza et al. (2013) |
| PO53-75 | meta-sedimentary | 164.2 | 1.62 | 2s | 88.40 | Talavera-Mendoza et al. (2013) |
| PO53-76 | meta-sedimentary | 169.1 | 2.14 | 2s | 88.40 | Talavera-Mendoza et al. (2013) |
| PO53-77 | meta-sedimentary | 142.2 | 13.03 | 2s | 88.40 | Talavera-Mendoza et al. (2013) |
| PO53-78 | meta-sedimentary | 144.1 | 12.71 | 2s | 88.40 | Talavera-Mendoza et al. (2013) |
| PO53-79 | meta-sedimentary | 154.4 | 3.63 | 2s | 88.40 | Talavera-Mendoza et al. (2013) |
| PO53-80 | meta-sedimentary | 173.0 | 5.19 | 2s | 88.40 | Talavera-Mendoza et al. (2013) |
| PO53-81 | meta-sedimentary | 170.4 | 9.52 | 2s | 88.40 | Talavera-Mendoza et al. (2013) |
| PO53-82 | meta-sedimentary | 176.2 | 7.48 | 2s | 88.40 | Talavera-Mendoza et al. (2013) |
| PO53-83 | meta-sedimentary | 178.8 | 5.96 | 2s | 88.40 | Talavera-Mendoza et al. (2013) |
| PO53-84 | meta-sedimentary | 171.0 | 2.99 | 2s | 88.40 | Talavera-Mendoza et al. (2013) |
| PO53-85 | meta-sedimentary | 178.5 | 2.17 | 2s | 88.40 | Talavera-Mendoza et al. (2013) |
| PO53-86 | meta-sedimentary | 188.5 | 5.69 | 2s | 88.40 | Talavera-Mendoza et al. (2013) |
| PO53-87 | meta-sedimentary | 169.2 | 4.41 | 2s | 88.40 | Talavera-Mendoza et al. (2013) |
| PO53-88 | meta-sedimentary | 181.3 | 2.63 | 2s | 88.40 | Talavera-Mendoza et al. (2013) |
| PO53-89 | meta-sedimentary | 178.6 | 3.68 | 2s | 88.40 | Talavera-Mendoza et al. (2013) |
| PO53-90 | meta-sedimentary | 158.8 | 3.90 | 2s | 88.40 | Talavera-Mendoza et al. (2013) |
| PO54 1 | meta-sedimentary | 156.4 | 6.23 | 2s | 90.74 | Talavera-Mendoza et al. (2013) |
| PO54 2 | meta-sedimentary | 173.4 | 1.71 | 2s | 90.74 | Talavera-Mendoza et al. (2013) |
| PO54 3 | meta-sedimentary | 201.8 | 12.26 | 2s | 90.74 | Talavera-Mendoza et al. (2013) |
| PO54 4 | meta-sedimentary | 180.8 | 4.15 | 2s | 90.74 | Talavera-Mendoza et al. (2013) |
| PO54 5 | meta-sedimentary | 145.7 | 8.09 | 2s | 90.74 | Talavera-Mendoza et al. (2013) |
| PO54 6 | meta-sedimentary | 176.2 | 4.21 | 2s | 90.74 | Talavera-Mendoza et al. (2013) |
| PO54-7 | meta-sedimentary | 178.0 | 3.13 | 2s | 90.74 | Talavera-Mendoza et al. (2013) |
| PO54-8 | meta-sedimentary | 175.4 | 1.73 | 2s | 90.74 | Talavera-Mendoza et al. (2013) |
| PO54-9 | meta-sedimentary | 164.9 | 6.12 | 2s | 90.74 | Talavera-Mendoza et al. (2013) |
| PO54-10 | meta-sedimentary | 182.0 | 1.79 | 2s | 90.74 | Talavera-Mendoza et al. (2013) |
| PO54-11 | meta-sedimentary | 166.9 | 2.19 | 2s | 90.74 | Talavera-Mendoza et al. (2013) |
| PO54-12 | meta-sedimentary | 181.8 | 2.78 | 2s | 90.74 | Talavera-Mendoza et al. (2013) |
| PO54-13 | meta-sedimentary | 173.1 | 1.74 | 2s | 90.74 | Talavera-Mendoza et al. (2013) |
| PO54-14 | meta-sedimentary | 180.3 | 1.78 | 2s | 90.74 | Talavera-Mendoza et al. (2013) |
| PO54-15 | meta-sedimentary | 206.4 | 4.65 | 2s | 90.74 | Talavera-Mendoza et al. (2013) |
| PO54-16 | meta-sedimentary | 175.7 | 1.73 | 2s | 90.74 | Talavera-Mendoza et al. (2013) |
| PO54-17 | meta-sedimentary | 95.7 | 5.21 | 2s | 90.74 | Talavera-Mendoza et al. (2013) |
| PO54-18 | meta-sedimentary | 176.0 | 1.74 | 2s | 90.74 | Talavera-Mendoza et al. (2013) |
| PO54-19 | meta-sedimentary | 178.9 | 1.76 | 2s | 90.74 | Talavera-Mendoza et al. (2013) |
| PO54-20 | meta-sedimentary | 156.4 | 2.30 | 2s | 90.74 | Talavera-Mendoza et al. (2013) |
| PO54-21 | meta-sedimentary | 164.1 | 5.12 | 2s | 90.74 | Talavera-Mendoza et al. (2013) |
| PO54-22 | meta-sedimentary | 169.5 | 1.81 | 2s | 90.74 | Talavera-Mendoza et al. (2013) |
| PO54-23 | meta-sedimentary | 169.2 | 7.60 | 2s | 90.74 | Talavera-Mendoza et al. (2013) |
| PO54-24 | meta-sedimentary | 141.6 | 2.58 | 2s | 90.74 | Talavera-Mendoza et al. (2013) |
| PO54-25 | meta-sedimentary | 134.6 | 7.14 | 2s | 90.74 | Talavera-Mendoza et al. (2013) |
| PO54-26 | meta-sedimentary | 174.4 | 2.41 | 2s | 90.74 | Talavera-Mendoza et al. (2013) |
| PO54-27 | meta-sedimentary | 179.6 | 2.21 | 2s | 90.74 | Talavera-Mendoza et al. (2013) |
| PO54-28 | meta-sedimentary | 182.4 | 5.56 | 2s | 90.74 | Talavera-Mendoza et al. (2013) |
| PO54-29 | meta-sedimentary | 187.1 | 3.01 | 2s | 90.74 | Talavera-Mendoza et al. (2013) |
| PO54-30 | meta-sedimentary | 199.3 | 5.91 | 2s | 90.74 | Talavera-Mendoza et al. (2013) |
| PO54-31 | meta-sedimentary | 151.9 | 2.60 | 2s | 90.74 | Talavera-Mendoza et al. (2013) |
| PO54-32 | meta-sedimentary | 182.9 | 2.94 | 2s | 90.74 | Talavera-Mendoza et al. (2013) |
| PO54-33 | meta-sedimentary | 168.0 | 6.92 | 2s | 90.74 | Talavera-Mendoza et al. (2013) |
| PO54-34 | meta-sedimentary | 51.4 | 1.07 | 2s | 90.74 | Talavera-Mendoza et al. (2013) |
| PO54-35 | meta-sedimentary | 179.5 | 2.74 | 2s | 90.74 | Talavera-Mendoza et al. (2013) |
| PO54-36 | meta-sedimentary | 239.6 | 2.61 | 2s | 90.74 | Talavera-Mendoza et al. (2013) |
| PO54-37 | meta-sedimentary | 191.9 | 7.05 | 2s | 90.74 | Talavera-Mendoza et al. (2013) |
| PO54-38 | meta-sedimentary | 186.9 | 3.85 | 2s | 90.74 | Talavera-Mendoza et al. (2013) |
| PO54-39 | meta-sedimentary | 191.9 | 1.89 | 2s | 90.74 | Talavera-Mendoza et al. (2013) |
| PO54-40 | meta-sedimentary | 180.8 | 10.34 | 2s | 90.74 | Talavera-Mendoza et al. (2013) |
| PO54-41 | meta-sedimentary | 160.8 | 11.87 | 2s | 90.74 | Talavera-Mendoza et al. (2013) |
| PO54-42 | meta-sedimentary | 171.5 | 1.69 | 2s | 90.74 | Talavera-Mendoza et al. (2013) |
| PO54-43 | meta-sedimentary | 182.9 | 3.61 | 2s | 90.74 | Talavera-Mendoza et al. (2013) |
| PO54-44 | meta-sedimentary | 183.0 | 3.45 | 2s | 90.74 | Talavera-Mendoza et al. (2013) |
| PO54-45 | meta-sedimentary | 184.7 | 1.82 | 2s | 90.74 | Talavera-Mendoza et al. (2013) |
| PO54-46 | meta-sedimentary | 191.2 | 2.73 | 2s | 90.74 | Talavera-Mendoza et al. (2013) |
| PO54-47 | meta-sedimentary | 180.6 | 2.12 | 2s | 90.74 | Talavera-Mendoza et al. (2013) |
| PO54-48 | meta-sedimentary | 773.6 | 7.29 | 2s | 90.74 | Talavera-Mendoza et al. (2013) |
| PO54-49 | meta-sedimentary | 176.6 | 3.94 | 2s | 90.74 | Talavera-Mendoza et al. (2013) |
| PO54-50 | meta-sedimentary | 180.3 | 2.38 | 2s | 90.74 | Talavera-Mendoza et al. (2013) |
| PO54-51 | meta-sedimentary | 206.1 | 3.04 | 2s | 90.74 | Talavera-Mendoza et al. (2013) |
| PO54-52 | meta-sedimentary | 148.5 | 3.10 | 2s | 90.74 | Talavera-Mendoza et al. (2013) |
| PO54-56 | meta-sedimentary | 60.6 | 3.45 | 2s | 90.74 | Talavera-Mendoza et al. (2013) |
| PO54-57 | meta-sedimentary | 57.4 | 5.08 | 2s | 90.74 | Talavera-Mendoza et al. (2013) |
| PO54-58 | meta-sedimentary | 58.5 | 3.47 | 2s | 90.74 | Talavera-Mendoza et al. (2013) |
| PO54-59 | meta-sedimentary | 169.4 | 4.01 | 2s | 90.74 | Talavera-Mendoza et al. (2013) |
| PO54-60 | meta-sedimentary | 188.3 | 3.10 | 2s | 90.74 | Talavera-Mendoza et al. (2013) |
| PO54-61 | meta-sedimentary | 59.5 | 2.87 | 2s | 90.74 | Talavera-Mendoza et al. (2013) |
| PO54-62 | meta-sedimentary | 56.6 | 1.44 | 2s | 90.74 | Talavera-Mendoza et al. (2013) |
| PO54-63 | meta-sedimentary | 55.6 | 2.15 | 2s | 90.74 | Talavera-Mendoza et al. (2013) |
| PO54-64 | meta-sedimentary | 58.8 | 2.60 | 2s | 90.74 | Talavera-Mendoza et al. (2013) |
| PO54-65 | meta-sedimentary | 188.6 | 1.86 | 2s | 90.74 | Talavera-Mendoza et al. (2013) |
| PO54-66 | meta-sedimentary | 169.0 | 2.67 | 2s | 90.74 | Talavera-Mendoza et al. (2013) |
| PO54-67 | meta-sedimentary | 192.0 | 4.12 | 2s | 90.74 | Talavera-Mendoza et al. (2013) |
| PO54-68 | meta-sedimentary | 177.5 | 2.52 | 2s | 90.74 | Talavera-Mendoza et al. (2013) |
| PO54-69 | meta-sedimentary | 174.0 | 3.02 | 2s | 90.74 | Talavera-Mendoza et al. (2013) |
| PO54-70 | meta-sedimentary | 172.1 | 3.23 | 2s | 90.74 | Talavera-Mendoza et al. (2013) |
| PO54-71 | meta-sedimentary | 179.6 | 6.53 | 2s | 90.74 | Talavera-Mendoza et al. (2013) |
| PO54-72 | meta-sedimentary | 181.9 | 6.85 | 2s | 90.74 | Talavera-Mendoza et al. (2013) |
| PO54-73 | meta-sedimentary | 159.3 | 2.55 | 2s | 90.74 | Talavera-Mendoza et al. (2013) |
| PO54-74 | meta-sedimentary | 176.7 | 3.59 | 2s | 90.74 | Talavera-Mendoza et al. (2013) |
| PO54-75 | meta-sedimentary | 178.2 | 1.99 | 2s | 90.74 | Talavera-Mendoza et al. (2013) |
| PO54-77 | meta-sedimentary | 180.8 | 2.71 | 2s | 90.74 | Talavera-Mendoza et al. (2013) |
| PO54-78 | meta-sedimentary | 174.8 | 3.10 | 2s | 90.74 | Talavera-Mendoza et al. (2013) |
| PO54-79 | meta-sedimentary | 174.7 | 2.36 | 2s | 90.74 | Talavera-Mendoza et al. (2013) |
| PO54-80 | meta-sedimentary | 167.8 | 4.74 | 2s | 90.74 | Talavera-Mendoza et al. (2013) |
| PO54-81 | meta-sedimentary | 176.0 | 4.31 | 2s | 90.74 | Talavera-Mendoza et al. (2013) |
| PO54-82 | meta-sedimentary | 144.0 | 5.84 | 2s | 90.74 | Talavera-Mendoza et al. (2013) |
| PO54-83 | meta-sedimentary | 178.4 | 4.01 | 2s | 90.74 | Talavera-Mendoza et al. (2013) |
| PO54-84 | meta-sedimentary | 185.4 | 2.16 | 2s | 90.74 | Talavera-Mendoza et al. (2013) |
| PO54-85 | meta-sedimentary | 145.5 | 7.42 | 2s | 90.74 | Talavera-Mendoza et al. (2013) |
| PO54-86 | meta-sedimentary | 145.2 | 4.94 | 2s | 90.74 | Talavera-Mendoza et al. (2013) |
| PO54-87 | meta-sedimentary | 35.0 | 0.75 | 2s | 90.74 | Talavera-Mendoza et al. (2013) |
| PO54-88 | meta-sedimentary | 155.7 | 2.97 | 2s | 90.74 | Talavera-Mendoza et al. (2013) |
| PO54-89 | meta-sedimentary | 70.5 | 1.57 | 2s | 90.74 | Talavera-Mendoza et al. (2013) |
| PO54-90 | meta-sedimentary | 65.5 | 1.86 | 2s | 90.74 | Talavera-Mendoza et al. (2013) |
| PO54-91 | meta-sedimentary | 61.3 | 0.61 | 2s | 90.74 | Talavera-Mendoza et al. (2013) |
| PO54-92 | meta-sedimentary | 165.6 | 7.62 | 2s | 90.74 | Talavera-Mendoza et al. (2013) |
| PO54-93 | meta-sedimentary | 120.5 | 3.21 | 2s | 90.74 | Talavera-Mendoza et al. (2013) |
| PO54-94 | meta-sedimentary | 168.4 | 2.88 | 2s | 90.74 | Talavera-Mendoza et al. (2013) |
| PO54-95 | meta-sedimentary | 167.3 | 4.56 | 2s | 90.74 | Talavera-Mendoza et al. (2013) |
| PO54-96 | meta-sedimentary | 51.4 | 3.37 | 2s | 90.74 | Talavera-Mendoza et al. (2013) |
| PO54-97 | meta-sedimentary | 57.4 | 0.98 | 2s | 90.74 | Talavera-Mendoza et al. (2013) |
| PO54-98 | meta-sedimentary | 64.0 | 0.64 | 2s | 90.74 | Talavera-Mendoza et al. (2013) |
| PO54-99 | meta-sedimentary | 172.9 | 1.71 | 2s | 90.74 | Talavera-Mendoza et al. (2013) |
| PO54-100 | meta-sedimentary | 62.0 | 1.95 | 2s | 90.74 | Talavera-Mendoza et al. (2013) |
| PO54-101 | meta-sedimentary | 51.0 | 0.51 | 2s | 90.74 | Talavera-Mendoza et al. (2013) |
| PO54-102 | meta-sedimentary | 62.2 | 0.62 | 2s | 90.74 | Talavera-Mendoza et al. (2013) |
| PO54-103 | meta-sedimentary | 167.4 | 1.65 | 2s | 90.74 | Talavera-Mendoza et al. (2013) |
| PO54-104 | meta-sedimentary | 107.2 | 5.25 | 2s | 90.74 | Talavera-Mendoza et al. (2013) |
| PO54-105 | meta-sedimentary | 164.1 | 2.25 | 2s | 90.74 | Talavera-Mendoza et al. (2013) |
| PO54-106 | meta-sedimentary | 168.5 | 4.62 | 2s | 90.74 | Talavera-Mendoza et al. (2013) |
| PO54-107 | meta-sedimentary | 137.5 | 1.77 | 2s | 90.74 | Talavera-Mendoza et al. (2013) |
| PO54-108 | meta-sedimentary | 45.6 | 3.61 | 2s | 90.74 | Talavera-Mendoza et al. (2013) |
| PO54-109 | meta-sedimentary | 169.1 | 3.35 | 2s | 90.74 | Talavera-Mendoza et al. (2013) |
| PO54-110 | meta-sedimentary | 67.4 | 1.51 | 2s | 90.74 | Talavera-Mendoza et al. (2013) |
| PO54-111 | meta-sedimentary | 171.8 | 7.51 | 2s | 90.74 | Talavera-Mendoza et al. (2013) |
| PO54-112 | meta-sedimentary | 178.4 | 2.18 | 2s | 90.74 | Talavera-Mendoza et al. (2013) |
| PO54-113 | meta-sedimentary | 141.9 | 3.33 | 2s | 90.74 | Talavera-Mendoza et al. (2013) |
| PO54-114 | meta-sedimentary | 91.2 | 3.99 | 2s | 90.74 | Talavera-Mendoza et al. (2013) |
| PO54-115 | meta-sedimentary | 52.3 | 0.90 | 2s | 90.74 | Talavera-Mendoza et al. (2013) |
| PO54-116 | meta-sedimentary | 52.2 | 0.82 | 2s | 90.74 | Talavera-Mendoza et al. (2013) |
| PO54-117 | meta-sedimentary | 60.6 | 2.36 | 2s | 90.74 | Talavera-Mendoza et al. (2013) |
| PO54-118 | meta-sedimentary | 60.2 | 0.60 | 2s | 90.74 | Talavera-Mendoza et al. (2013) |
| PO213A-1 | meta-sedimentary | 305.1 | 2.98 | 2s | 451.83 | Talavera-Mendoza et al. (2013) |
| PO213A-2 | meta-sedimentary | 287.9 | 6.87 | 2s | 451.83 | Talavera-Mendoza et al. (2013) |
| PO213A-3 | meta-sedimentary | 293.1 | 5.90 | 2s | 451.83 | Talavera-Mendoza et al. (2013) |
| PO213A-4 | meta-sedimentary | 301.8 | 3.42 | 2s | 451.83 | Talavera-Mendoza et al. (2013) |
| PO213A-5 | meta-sedimentary | 125.1 | 11.93 | 2s | 451.83 | Talavera-Mendoza et al. (2013) |
| PO213A-7 | meta-sedimentary | 318.1 | 4.87 | 2s | 451.83 | Talavera-Mendoza et al. (2013) |
| PO213A-9 | meta-sedimentary | 312.0 | 3.62 | 2s | 451.83 | Talavera-Mendoza et al. (2013) |
| PO213A-8 | meta-sedimentary | 305.2 | 6.71 | 2s | 451.83 | Talavera-Mendoza et al. (2013) |
| PO213A-10 | meta-sedimentary | 234.5 | 7.81 | 2s | 451.83 | Talavera-Mendoza et al. (2013) |
| PO213A-11 | meta-sedimentary | 265.4 | 25.90 | 2s | 451.83 | Talavera-Mendoza et al. (2013) |
| PO213A-12 | meta-sedimentary | 178.5 | 5.83 | 2s | 451.83 | Talavera-Mendoza et al. (2013) |
| PO213A-13 | meta-sedimentary | 102.3 | 6.03 | 2s | 451.83 | Talavera-Mendoza et al. (2013) |
| PO213A-14 | meta-sedimentary | 154.3 | 8.66 | 2s | 451.83 | Talavera-Mendoza et al. (2013) |
| PO213A-15 | meta-sedimentary | 301.6 | 15.94 | 2s | 451.83 | Talavera-Mendoza et al. (2013) |
| PO213A-16 | meta-sedimentary | 311.4 | 4.99 | 2s | 451.83 | Talavera-Mendoza et al. (2013) |
| PO213A-17 | meta-sedimentary | 293.7 | 9.39 | 2s | 451.83 | Talavera-Mendoza et al. (2013) |
| PO213A-18 | meta-sedimentary | 252.7 | 2.97 | 2s | 451.83 | Talavera-Mendoza et al. (2013) |
| PO213A-19 | meta-sedimentary | 306.3 | 4.31 | 2s | 451.83 | Talavera-Mendoza et al. (2013) |
| PO213A-20 | meta-sedimentary | 287.0 | 4.52 | 2s | 451.83 | Talavera-Mendoza et al. (2013) |
| PO213A-21 | meta-sedimentary | 283.9 | 13.00 | 2s | 451.83 | Talavera-Mendoza et al. (2013) |
| PO213A-22 | meta-sedimentary | 311.9 | 3.04 | 2s | 451.83 | Talavera-Mendoza et al. (2013) |
| PO213A-23 | meta-sedimentary | 132.9 | 12.49 | 2s | 451.83 | Talavera-Mendoza et al. (2013) |
| PO213A-24 | meta-sedimentary | 56.0 | 3.67 | 2s | 451.83 | Talavera-Mendoza et al. (2013) |
| PO213A-25 | meta-sedimentary | 293.6 | 5.71 | 2s | 451.83 | Talavera-Mendoza et al. (2013) |
| PO213A-27 | meta-sedimentary | 296.0 | 10.50 | 2s | 451.83 | Talavera-Mendoza et al. (2013) |
| PO213A-28 | meta-sedimentary | 225.4 | 3.72 | 2s | 451.83 | Talavera-Mendoza et al. (2013) |
| PO213A-29 | meta-sedimentary | 141.4 | 6.85 | 2s | 451.83 | Talavera-Mendoza et al. (2013) |
| PO213A-30 | meta-sedimentary | 58.6 | 3.73 | 2s | 451.83 | Talavera-Mendoza et al. (2013) |
| PO213A-31 | meta-sedimentary | 295.7 | 2.89 | 2s | 451.83 | Talavera-Mendoza et al. (2013) |
| PO213A-32 | meta-sedimentary | 143.7 | 4.46 | 2s | 451.83 | Talavera-Mendoza et al. (2013) |
| PO213A-33 | meta-sedimentary | 297.7 | 7.24 | 2s | 451.83 | Talavera-Mendoza et al. (2013) |
| PO213A-36 | meta-sedimentary | 166.6 | 2.47 | 2s | 451.83 | Talavera-Mendoza et al. (2013) |
| PO213A-37 | meta-sedimentary | 223.1 | 17.80 | 2s | 451.83 | Talavera-Mendoza et al. (2013) |
| PO213A-38 | meta-sedimentary | 155.6 | 9.98 | 2s | 451.83 | Talavera-Mendoza et al. (2013) |
| PO213A-39 | meta-sedimentary | 64.4 | 4.19 | 2s | 451.83 | Talavera-Mendoza et al. (2013) |
| PO213A-40 | meta-sedimentary | 306.4 | 3.41 | 2s | 451.83 | Talavera-Mendoza et al. (2013) |
| PO213A-41 | meta-sedimentary | 267.1 | 3.74 | 2s | 451.83 | Talavera-Mendoza et al. (2013) |
| PO213A-43 | meta-sedimentary | 200.0 | 11.07 | 2s | 451.83 | Talavera-Mendoza et al. (2013) |
| PO213A-44 | meta-sedimentary | 301.1 | 2.94 | 2s | 451.83 | Talavera-Mendoza et al. (2013) |
| PO213A-45 | meta-sedimentary | 259.0 | 7.54 | 2s | 451.83 | Talavera-Mendoza et al. (2013) |
| PO213A-46 | meta-sedimentary | 137.2 | 7.70 | 2s | 451.83 | Talavera-Mendoza et al. (2013) |
| PO213A-47 | meta-sedimentary | 106.1 | 4.06 | 2s | 451.83 | Talavera-Mendoza et al. (2013) |
| PO213A-48 | meta-sedimentary | 236.5 | 4.25 | 2s | 451.83 | Talavera-Mendoza et al. (2013) |
| PO213A-50 | meta-sedimentary | 240.3 | 11.63 | 2s | 451.83 | Talavera-Mendoza et al. (2013) |
| PO213A-51 | meta-sedimentary | 288.3 | 10.77 | 2s | 451.83 | Talavera-Mendoza et al. (2013) |
| PO213A-52 | meta-sedimentary | 339.6 | 16.31 | 2s | 451.83 | Talavera-Mendoza et al. (2013) |
| PO213A-53 | meta-sedimentary | 96.4 | 6.07 | 2s | 451.83 | Talavera-Mendoza et al. (2013) |
| PO213A-54 | meta-sedimentary | 295.5 | 5.14 | 2s | 451.83 | Talavera-Mendoza et al. (2013) |
| PO213A-55 | meta-sedimentary | 235.3 | 5.08 | 2s | 451.83 | Talavera-Mendoza et al. (2013) |
| PO213A-56 | meta-sedimentary | 251.9 | 13.19 | 2s | 451.83 | Talavera-Mendoza et al. (2013) |
| PO213A-57 | meta-sedimentary | 44.9 | 3.33 | 2s | 451.83 | Talavera-Mendoza et al. (2013) |
| PO213A-58 | meta-sedimentary | 94.3 | 6.13 | 2s | 451.83 | Talavera-Mendoza et al. (2013) |
| PO213A-59 | meta-sedimentary | 66.1 | 3.14 | 2s | 451.83 | Talavera-Mendoza et al. (2013) |
| PO213A-61 | meta-sedimentary | 137.8 | 3.49 | 2s | 451.83 | Talavera-Mendoza et al. (2013) |
| PO213A-62 | meta-sedimentary | 257.2 | 5.24 | 2s | 451.83 | Talavera-Mendoza et al. (2013) |
| PO213A-63 | meta-sedimentary | 139.7 | 4.31 | 2s | 451.83 | Talavera-Mendoza et al. (2013) |
| PO213A-64 | meta-sedimentary | 64.7 | 3.00 | 2s | 451.83 | Talavera-Mendoza et al. (2013) |
| PO213A-65 | meta-sedimentary | 321.4 | 6.58 | 2s | 451.83 | Talavera-Mendoza et al. (2013) |
| PO213A-66 | meta-sedimentary | 290.8 | 4.49 | 2s | 451.83 | Talavera-Mendoza et al. (2013) |
| PO213A-67 | meta-sedimentary | 290.0 | 7.77 | 2s | 451.83 | Talavera-Mendoza et al. (2013) |
| PO213A-69 | meta-sedimentary | 229.5 | 11.02 | 2s | 451.83 | Talavera-Mendoza et al. (2013) |
| PO213A-70 | meta-sedimentary | 170.9 | 5.08 | 2s | 451.83 | Talavera-Mendoza et al. (2013) |
| PO213A-71 | meta-sedimentary | 306.9 | 6.38 | 2s | 451.83 | Talavera-Mendoza et al. (2013) |
| PO213A-72 | meta-sedimentary | 129.5 | 3.20 | 2s | 451.83 | Talavera-Mendoza et al. (2013) |
| PO213A-73 | meta-sedimentary | 283.8 | 3.55 | 2s | 451.83 | Talavera-Mendoza et al. (2013) |
| PO213A-74 | meta-sedimentary | 201.9 | 9.36 | 2s | 451.83 | Talavera-Mendoza et al. (2013) |
| PO213A-75 | meta-sedimentary | 263.5 | 2.74 | 2s | 451.83 | Talavera-Mendoza et al. (2013) |
| PO213A-76 | meta-sedimentary | 286.3 | 23.10 | 2s | 451.83 | Talavera-Mendoza et al. (2013) |
| PO213A-77 | meta-sedimentary | 257.3 | 2.52 | 2s | 451.83 | Talavera-Mendoza et al. (2013) |
| PO213A-79 | meta-sedimentary | 270.3 | 9.40 | 2s | 451.83 | Talavera-Mendoza et al. (2013) |
| PO213A-80 | meta-sedimentary | 292.3 | 3.34 | 2s | 451.83 | Talavera-Mendoza et al. (2013) |
| PO213A-81 | meta-sedimentary | 130.9 | 2.89 | 2s | 451.83 | Talavera-Mendoza et al. (2013) |
| PO213A-82 | meta-sedimentary | 276.8 | 5.15 | 2s | 451.83 | Talavera-Mendoza et al. (2013) |
| PO213A-83 | meta-sedimentary | 83.8 | 2.76 | 2s | 451.83 | Talavera-Mendoza et al. (2013) |
| PO213A-84 | meta-sedimentary | 322.3 | 3.14 | 2s | 451.83 | Talavera-Mendoza et al. (2013) |
| PO213A-86 | meta-sedimentary | 279.9 | 6.13 | 2s | 451.83 | Talavera-Mendoza et al. (2013) |
| PO213A-87 | meta-sedimentary | 262.1 | 5.47 | 2s | 451.83 | Talavera-Mendoza et al. (2013) |
| PO213A-88 | meta-sedimentary | 316.3 | 7.38 | 2s | 451.83 | Talavera-Mendoza et al. (2013) |
| PO213A-89 | meta-sedimentary | 345.2 | 7.80 | 2s | 451.83 | Talavera-Mendoza et al. (2013) |
| PO213A-90 | meta-sedimentary | 262.8 | 2.68 | 2s | 451.83 | Talavera-Mendoza et al. (2013) |
| PO213A-91 | meta-sedimentary | 315.5 | 7.14 | 2s | 451.83 | Talavera-Mendoza et al. (2013) |
| PO213A-92 | meta-sedimentary | 306.9 | 3.00 | 2s | 451.83 | Talavera-Mendoza et al. (2013) |
| PO213A-93 | meta-sedimentary | 131.7 | 4.07 | 2s | 451.83 | Talavera-Mendoza et al. (2013) |
| PO213A-94 | meta-sedimentary | 285.5 | 5.56 | 2s | 451.83 | Talavera-Mendoza et al. (2013) |
| PO213A-95 | meta-sedimentary | 307.2 | 3.00 | 2s | 451.83 | Talavera-Mendoza et al. (2013) |
| PO213A-97 | meta-sedimentary | 325.9 | 5.88 | 2s | 451.83 | Talavera-Mendoza et al. (2013) |
| PO213A-98 | meta-sedimentary | 325.9 | 6.10 | 2s | 451.83 | Talavera-Mendoza et al. (2013) |
| PO213A-99 | meta-sedimentary | 305.4 | 2.98 | 2s | 451.83 | Talavera-Mendoza et al. (2013) |
| PO213A-100 | meta-sedimentary | 214.2 | 10.13 | 2s | 451.83 | Talavera-Mendoza et al. (2013) |
| PO230-1 | meta-sedimentary | 133.8 | 1.54 | 2s | 97.88 | Talavera-Mendoza et al. (2013) |
| PO230-2 | meta-sedimentary | 141.2 | 3.10 | 2s | 97.88 | Talavera-Mendoza et al. (2013) |
| PO230-3 | meta-sedimentary | 151.0 | 1.49 | 2s | 97.88 | Talavera-Mendoza et al. (2013) |
| PO230-4 | meta-sedimentary | 136.2 | 2.33 | 2s | 97.88 | Talavera-Mendoza et al. (2013) |
| PO230-5 | meta-sedimentary | 140.0 | 2.33 | 2s | 97.88 | Talavera-Mendoza et al. (2013) |
| PO230-6 | meta-sedimentary | 140.2 | 7.19 | 2s | 97.88 | Talavera-Mendoza et al. (2013) |
| PO230-7 | meta-sedimentary | 138.4 | 3.25 | 2s | 97.88 | Talavera-Mendoza et al. (2013) |
| PO230-8 | meta-sedimentary | 137.6 | 6.18 | 2s | 97.88 | Talavera-Mendoza et al. (2013) |
| PO230-9 | meta-sedimentary | 143.6 | 2.81 | 2s | 97.88 | Talavera-Mendoza et al. (2013) |
| PO230-10 | meta-sedimentary | 156.5 | 6.26 | 2s | 97.88 | Talavera-Mendoza et al. (2013) |
| PO230-11 | meta-sedimentary | 161.0 | 4.15 | 2s | 97.88 | Talavera-Mendoza et al. (2013) |
| PO230-12 | meta-sedimentary | 118.5 | 3.89 | 2s | 97.88 | Talavera-Mendoza et al. (2013) |
| PO230-13 | meta-sedimentary | 79.3 | 1.46 | 2s | 97.88 | Talavera-Mendoza et al. (2013) |
| PO230-15 | meta-sedimentary | 126.5 | 2.15 | 2s | 97.88 | Talavera-Mendoza et al. (2013) |
| PO230-16 | meta-sedimentary | 143.8 | 5.40 | 2s | 97.88 | Talavera-Mendoza et al. (2013) |
| PO230-17 | meta-sedimentary | 144.5 | 1.59 | 2s | 97.88 | Talavera-Mendoza et al. (2013) |
| PO230-18 | meta-sedimentary | 138.1 | 1.56 | 2s | 97.88 | Talavera-Mendoza et al. (2013) |
| PO230-19 | meta-sedimentary | 137.8 | 3.37 | 2s | 97.88 | Talavera-Mendoza et al. (2013) |
| PO230-20 | meta-sedimentary | 131.7 | 8.69 | 2s | 97.88 | Talavera-Mendoza et al. (2013) |
| PO230-21 | meta-sedimentary | 134.1 | 2.08 | 2s | 97.88 | Talavera-Mendoza et al. (2013) |
| PO230-22 | meta-sedimentary | 135.9 | 2.25 | 2s | 97.88 | Talavera-Mendoza et al. (2013) |
| PO230-23 | meta-sedimentary | 135.8 | 2.90 | 2s | 97.88 | Talavera-Mendoza et al. (2013) |
| PO230-24 | meta-sedimentary | 132.0 | 1.41 | 2s | 97.88 | Talavera-Mendoza et al. (2013) |
| PO230-25 | meta-sedimentary | 138.7 | 1.91 | 2s | 97.88 | Talavera-Mendoza et al. (2013) |
| PO230-26 | meta-sedimentary | 156.8 | 4.48 | 2s | 97.88 | Talavera-Mendoza et al. (2013) |
| PO230-28 | meta-sedimentary | 127.4 | 1.48 | 2s | 97.88 | Talavera-Mendoza et al. (2013) |
| PO230-29 | meta-sedimentary | 133.5 | 3.44 | 2s | 97.88 | Talavera-Mendoza et al. (2013) |
| PO230-30 | meta-sedimentary | 140.0 | 3.56 | 2s | 97.88 | Talavera-Mendoza et al. (2013) |
| PO230-31 | meta-sedimentary | 123.7 | 2.45 | 2s | 97.88 | Talavera-Mendoza et al. (2013) |
| PO230-32 | meta-sedimentary | 137.6 | 2.19 | 2s | 97.88 | Talavera-Mendoza et al. (2013) |
| PO230-34 | meta-sedimentary | 103.3 | 3.43 | 2s | 97.88 | Talavera-Mendoza et al. (2013) |
| PO230-35 | meta-sedimentary | 138.5 | 2.71 | 2s | 97.88 | Talavera-Mendoza et al. (2013) |
| PO230-36 | meta-sedimentary | 86.5 | 1.16 | 2s | 97.88 | Talavera-Mendoza et al. (2013) |
| PO230-37 | meta-sedimentary | 115.0 | 3.15 | 2s | 97.88 | Talavera-Mendoza et al. (2013) |
| PO230-38 | meta-sedimentary | 149.0 | 10.12 | 2s | 97.88 | Talavera-Mendoza et al. (2013) |
| PO230-39 | meta-sedimentary | 136.5 | 3.81 | 2s | 97.88 | Talavera-Mendoza et al. (2013) |
| PO230-40 | meta-sedimentary | 64.0 | 2.13 | 2s | 97.88 | Talavera-Mendoza et al. (2013) |
| PO230-40A | meta-sedimentary | 64.4 | 1.22 | 2s | 97.88 | Talavera-Mendoza et al. (2013) |
| PO230-41 | meta-sedimentary | 128.1 | 1.75 | 2s | 97.88 | Talavera-Mendoza et al. (2013) |
| PO230-42 | meta-sedimentary | 133.6 | 3.74 | 2s | 97.88 | Talavera-Mendoza et al. (2013) |
| PO230-43 | meta-sedimentary | 117.9 | 5.41 | 2s | 97.88 | Talavera-Mendoza et al. (2013) |
| PO230-44 | meta-sedimentary | 139.3 | 1.41 | 2s | 97.88 | Talavera-Mendoza et al. (2013) |
| PO230-45 | meta-sedimentary | 137.8 | 1.36 | 2s | 97.88 | Talavera-Mendoza et al. (2013) |
| PO230-46 | meta-sedimentary | 133.7 | 1.89 | 2s | 97.88 | Talavera-Mendoza et al. (2013) |
| PO230-47 | meta-sedimentary | 140.2 | 1.89 | 2s | 97.88 | Talavera-Mendoza et al. (2013) |
| PO230-48 | meta-sedimentary | 135.6 | 1.72 | 2s | 97.88 | Talavera-Mendoza et al. (2013) |
| PO230-49 | meta-sedimentary | 132.6 | 3.96 | 2s | 97.88 | Talavera-Mendoza et al. (2013) |
| PO230-50 | meta-sedimentary | 125.8 | 5.28 | 2s | 97.88 | Talavera-Mendoza et al. (2013) |
| PO230-51 | meta-sedimentary | 116.7 | 4.59 | 2s | 97.88 | Talavera-Mendoza et al. (2013) |
| PO230-52 | meta-sedimentary | 136.4 | 2.36 | 2s | 97.88 | Talavera-Mendoza et al. (2013) |
| PO230-53 | meta-sedimentary | 121.9 | 1.98 | 2s | 97.88 | Talavera-Mendoza et al. (2013) |
| PO230-54 | meta-sedimentary | 131.3 | 1.30 | 2s | 97.88 | Talavera-Mendoza et al. (2013) |
| PO230-55 | meta-sedimentary | 135.5 | 2.33 | 2s | 97.88 | Talavera-Mendoza et al. (2013) |
| PO230-56 | meta-sedimentary | 132.3 | 3.23 | 2s | 97.88 | Talavera-Mendoza et al. (2013) |
| PO230-57 | meta-sedimentary | 132.4 | 2.39 | 2s | 97.88 | Talavera-Mendoza et al. (2013) |
| PO230-58 | meta-sedimentary | 147.6 | 1.65 | 2s | 97.88 | Talavera-Mendoza et al. (2013) |
| PO230-59 | meta-sedimentary | 139.3 | 1.65 | 2s | 97.88 | Talavera-Mendoza et al. (2013) |
| PO230-60 | meta-sedimentary | 127.6 | 4.18 | 2s | 97.88 | Talavera-Mendoza et al. (2013) |
| PO230-61 | meta-sedimentary | 139.7 | 1.48 | 2s | 97.88 | Talavera-Mendoza et al. (2013) |
| PO230-62 | meta-sedimentary | 132.5 | 1.31 | 2s | 97.88 | Talavera-Mendoza et al. (2013) |
| PO230-63 | meta-sedimentary | 101.8 | 1.06 | 2s | 97.88 | Talavera-Mendoza et al. (2013) |
| PO230-64 | meta-sedimentary | 113.5 | 1.67 | 2s | 97.88 | Talavera-Mendoza et al. (2013) |
| PO230-65 | meta-sedimentary | 128.5 | 10.04 | 2s | 97.88 | Talavera-Mendoza et al. (2013) |
| PO230-66 | meta-sedimentary | 134.7 | 3.40 | 2s | 97.88 | Talavera-Mendoza et al. (2013) |
| PO230-67 | meta-sedimentary | 131.9 | 2.49 | 2s | 97.88 | Talavera-Mendoza et al. (2013) |
| PO230-68 | meta-sedimentary | 108.3 | 1.15 | 2s | 97.88 | Talavera-Mendoza et al. (2013) |
| PO230-69 | meta-sedimentary | 131.8 | 1.41 | 2s | 97.88 | Talavera-Mendoza et al. (2013) |
| PO230-70 | meta-sedimentary | 96.2 | 3.80 | 2s | 97.88 | Talavera-Mendoza et al. (2013) |
| PO230-71 | meta-sedimentary | 130.7 | 3.72 | 2s | 97.88 | Talavera-Mendoza et al. (2013) |
| PO230-72 | meta-sedimentary | 130.0 | 2.24 | 2s | 97.88 | Talavera-Mendoza et al. (2013) |
| PO230-73 | meta-sedimentary | 112.2 | 3.58 | 2s | 97.88 | Talavera-Mendoza et al. (2013) |
| PO230-74 | meta-sedimentary | 134.6 | 1.33 | 2s | 97.88 | Talavera-Mendoza et al. (2013) |
| PO230-75 | meta-sedimentary | 135.4 | 1.57 | 2s | 97.88 | Talavera-Mendoza et al. (2013) |
| PO230-76 | meta-sedimentary | 120.7 | 3.79 | 2s | 97.88 | Talavera-Mendoza et al. (2013) |
| PO230-77 | meta-sedimentary | 102.9 | 2.27 | 2s | 97.88 | Talavera-Mendoza et al. (2013) |
| PO230-78 | meta-sedimentary | 138.5 | 1.45 | 2s | 97.88 | Talavera-Mendoza et al. (2013) |
| PO230-79 | meta-sedimentary | 86.5 | 0.94 | 2s | 97.88 | Talavera-Mendoza et al. (2013) |
| PO230-80 | meta-sedimentary | 108.0 | 1.07 | 2s | 97.88 | Talavera-Mendoza et al. (2013) |
| PO230-81 | meta-sedimentary | 134.0 | 3.16 | 2s | 97.88 | Talavera-Mendoza et al. (2013) |
| PO230-82 | meta-sedimentary | 133.2 | 3.31 | 2s | 97.88 | Talavera-Mendoza et al. (2013) |
| PO230-83 | meta-sedimentary | 132.0 | 1.93 | 2s | 97.88 | Talavera-Mendoza et al. (2013) |
| PO230-84 | meta-sedimentary | 119.3 | 1.25 | 2s | 97.88 | Talavera-Mendoza et al. (2013) |
| PO230-85 | meta-sedimentary | 114.0 | 4.28 | 2s | 97.88 | Talavera-Mendoza et al. (2013) |
| PO230-86 | meta-sedimentary | 133.1 | 3.03 | 2s | 97.88 | Talavera-Mendoza et al. (2013) |
| PO230-87 | meta-sedimentary | 129.9 | 7.77 | 2s | 97.88 | Talavera-Mendoza et al. (2013) |
| PO230-88 | meta-sedimentary | 139.7 | 4.27 | 2s | 97.88 | Talavera-Mendoza et al. (2013) |
| PO230-89 | meta-sedimentary | 135.2 | 1.54 | 2s | 97.88 | Talavera-Mendoza et al. (2013) |
| PO230-90 | meta-sedimentary | 138.5 | 1.67 | 2s | 97.88 | Talavera-Mendoza et al. (2013) |
| PO230-91 | meta-sedimentary | 135.9 | 1.34 | 2s | 97.88 | Talavera-Mendoza et al. (2013) |
| PO230-92 | meta-sedimentary | 141.4 | 1.40 | 2s | 97.88 | Talavera-Mendoza et al. (2013) |
| PO230-93 | meta-sedimentary | 133.4 | 1.32 | 2s | 97.88 | Talavera-Mendoza et al. (2013) |
| PO230-94 | meta-sedimentary | 142.2 | 4.18 | 2s | 97.88 | Talavera-Mendoza et al. (2013) |
| PO230-95 | meta-sedimentary | 123.8 | 3.45 | 2s | 97.88 | Talavera-Mendoza et al. (2013) |
| PO230-96 | meta-sedimentary | 113.3 | 2.65 | 2s | 97.88 | Talavera-Mendoza et al. (2013) |
| PO230-97 | meta-sedimentary | 131.1 | 3.12 | 2s | 97.88 | Talavera-Mendoza et al. (2013) |
| PO230-98 | meta-sedimentary | 138.5 | 2.48 | 2s | 97.88 | Talavera-Mendoza et al. (2013) |
| PO230-99 | meta-sedimentary | 131.8 | 2.39 | 2s | 97.88 | Talavera-Mendoza et al. (2013) |
| PO230-100 | meta-sedimentary | 115.1 | 7.16 | 2s | 97.88 | Talavera-Mendoza et al. (2013) |
| PEZ08 | Leucosome | 290.1 | 8.60 | 2s | 392.95 | Peña-Alonso et al. (2017) |
| PEZ08 | Leucosome | 281.5 | 8.70 | 2s | 392.95 | Peña-Alonso et al. (2017) |
| PEZ08 | Leucosome | 34.5 | 1.30 | 2s | 392.95 | Peña-Alonso et al. (2017) |
| PEZ08 | Leucosome | 296.0 | 11.00 | 2s | 392.95 | Peña-Alonso et al. (2017) |
| PEZ08 | Leucosome | 26.8 | 0.78 | 2s | 392.95 | Peña-Alonso et al. (2017) |
| PEZ08 | Leucosome | 301.0 | 17.00 | 2s | 392.95 | Peña-Alonso et al. (2017) |
| PEZ08 | Leucosome | 34.5 | 0.98 | 2s | 392.95 | Peña-Alonso et al. (2017) |
| PEZ08 | Leucosome | 42.4 | 1.70 | 2s | 392.95 | Peña-Alonso et al. (2017) |
| PEZ08 | Leucosome | 25.9 | 0.78 | 2s | 392.95 | Peña-Alonso et al. (2017) |
| PEZ08 | Leucosome | 281.0 | 19.00 | 2s | 392.95 | Peña-Alonso et al. (2017) |
| PEZ08 | Leucosome | 273.8 | 8.10 | 2s | 392.95 | Peña-Alonso et al. (2017) |
| PEZ08 | Leucosome | 135.6 | 5.30 | 2s | 392.95 | Peña-Alonso et al. (2017) |
| PEZ08 | Leucosome | 294.0 | 11.00 | 2s | 392.95 | Peña-Alonso et al. (2017) |
| PEZ08 | Leucosome | 305.5 | 9.10 | 2s | 392.95 | Peña-Alonso et al. (2017) |
| PEZ08 | Leucosome | 118.0 | 14.00 | 2s | 392.95 | Peña-Alonso et al. (2017) |
| PEZ08 | Leucosome | 272.1 | 7.50 | 2s | 392.95 | Peña-Alonso et al. (2017) |
| PEZ08 | Leucosome | 268.0 | 8.60 | 2s | 392.95 | Peña-Alonso et al. (2017) |
| PEZ08 | Leucosome | 26.0 | 0.80 | 2s | 392.95 | Peña-Alonso et al. (2017) |
| PEZ08 | Leucosome | 28.0 | 0.78 | 2s | 392.95 | Peña-Alonso et al. (2017) |
| PEZ08 | Leucosome | 24.1 | 0.60 | 2s | 392.95 | Peña-Alonso et al. (2017) |
| PEZ08 | Leucosome | 24.5 | 0.60 | 2s | 392.95 | Peña-Alonso et al. (2017) |
| PEZ08 | Leucosome | 28.8 | 0.92 | 2s | 392.95 | Peña-Alonso et al. (2017) |
| PEZ42 | Leucosome | 26.4 | 1.50 | 2s | 397.13 | Peña-Alonso et al. (2017) |
| PEZ42 | Leucosome | 23.1 | 0.62 | 2s | 397.13 | Peña-Alonso et al. (2017) |
| PEZ42 | Leucosome | 22.7 | 0.63 | 2s | 397.13 | Peña-Alonso et al. (2017) |
| PEZ42 | Leucosome | 23.8 | 0.67 | 2s | 397.13 | Peña-Alonso et al. (2017) |
| PEZ42 | Leucosome | 723.0 | 17.00 | 2s | 397.13 | Peña-Alonso et al. (2017) |
| PEZ42 | Leucosome | 785.0 | 19.00 | 2s | 397.13 | Peña-Alonso et al. (2017) |
| PEZ42 | Leucosome | 1105.0 | 30.00 | 2s | 397.13 | Peña-Alonso et al. (2017) |
| PEZ42 | Leucosome | 1063.0 | 24.00 | 2s | 397.13 | Peña-Alonso et al. (2017) |
| PEZ42 | Leucosome | 1225.0 | 37.00 | 2s | 397.13 | Peña-Alonso et al. (2017) |
| PEZ42 | Leucosome | 830.0 | 19.00 | 2s | 397.13 | Peña-Alonso et al. (2017) |
| PEZ42 | Leucosome | 25.5 | 2.40 | 2s | 397.13 | Peña-Alonso et al. (2017) |
| PEZ42 | Leucosome | 1153.0 | 21.00 | 2s | 397.13 | Peña-Alonso et al. (2017) |
| PEZ42 | Leucosome | 27.0 | 2.10 | 2s | 397.13 | Peña-Alonso et al. (2017) |
| PEZ42 | Leucosome | 23.5 | 0.62 | 2s | 397.13 | Peña-Alonso et al. (2017) |
| PEZ42 | Leucosome | 261.0 | 14.00 | 2s | 397.13 | Peña-Alonso et al. (2017) |
| PEZ42 | Leucosome | 22.2 | 1.10 | 2s | 397.13 | Peña-Alonso et al. (2017) |
| PEZ42 | Leucosome | 23.6 | 0.71 | 2s | 397.13 | Peña-Alonso et al. (2017) |
| PEZ42 | Leucosome | 891.0 | 19.00 | 2s | 397.13 | Peña-Alonso et al. (2017) |
| PEZ42 | Leucosome | 649.0 | 41.00 | 2s | 397.13 | Peña-Alonso et al. (2017) |
| PEZ42 | Leucosome | 913.0 | 20.00 | 2s | 397.13 | Peña-Alonso et al. (2017) |
| PEZ42 | Leucosome | 274.8 | 6.90 | 2s | 397.13 | Peña-Alonso et al. (2017) |
| PEZ42 | Leucosome | 165.9 | 6.10 | 2s | 397.13 | Peña-Alonso et al. (2017) |
| PEZ42 | Leucosome | 252.4 | 6.20 | 2s | 397.13 | Peña-Alonso et al. (2017) |
| PEZ27 | Leucosome | 56.1 | 2.00 | 2s | 378.27 | Peña-Alonso et al. (2017) |
| PEZ27 | Leucosome | 59.5 | 1.70 | 2s | 378.27 | Peña-Alonso et al. (2017) |
| PEZ27 | Leucosome | 58.1 | 2.10 | 2s | 378.27 | Peña-Alonso et al. (2017) |
| PEZ27 | Leucosome | 57.5 | 2.00 | 2s | 378.27 | Peña-Alonso et al. (2017) |
| PEZ27 | Leucosome | 59.1 | 1.70 | 2s | 378.27 | Peña-Alonso et al. (2017) |
| PEZ27 | Leucosome | 55.8 | 1.90 | 2s | 378.27 | Peña-Alonso et al. (2017) |
| PEZ27 | Leucosome | 55.9 | 1.80 | 2s | 378.27 | Peña-Alonso et al. (2017) |
| PEZ27 | Leucosome | 55.6 | 1.60 | 2s | 378.27 | Peña-Alonso et al. (2017) |
| PEZ27 | Leucosome | 59.6 | 2.00 | 2s | 378.27 | Peña-Alonso et al. (2017) |
| PEZ27 | Leucosome | 58.0 | 1.60 | 2s | 378.27 | Peña-Alonso et al. (2017) |
| PEZ27 | Leucosome | 59.6 | 1.90 | 2s | 378.27 | Peña-Alonso et al. (2017) |
| PEZ27 | Leucosome | 57.2 | 1.90 | 2s | 378.27 | Peña-Alonso et al. (2017) |
| PEZ27 | Leucosome | 57.7 | 1.60 | 2s | 378.27 | Peña-Alonso et al. (2017) |
| PEZ27 | Leucosome | 1315.0 | 37.00 | 2s | 378.27 | Peña-Alonso et al. (2017) |
| PEZ27 | Leucosome | 26.8 | 0.68 | 2s | 378.27 | Peña-Alonso et al. (2017) |
| PEZ27 | Leucosome | 56.5 | 1.60 | 2s | 378.27 | Peña-Alonso et al. (2017) |
| PEZ27 | Leucosome | 59.5 | 2.00 | 2s | 378.27 | Peña-Alonso et al. (2017) |
| PEZ27 | Leucosome | 58.0 | 1.60 | 2s | 378.27 | Peña-Alonso et al. (2017) |
| PEZ27 | Leucosome | 57.4 | 1.50 | 2s | 378.27 | Peña-Alonso et al. (2017) |
| PEZ27 | Leucosome | 56.7 | 1.60 | 2s | 378.27 | Peña-Alonso et al. (2017) |
| PEZ27 | Leucosome | 49.6 | 2.20 | 2s | 378.27 | Peña-Alonso et al. (2017) |
| PEZ27 | Leucosome | 56.1 | 1.90 | 2s | 378.27 | Peña-Alonso et al. (2017) |
| PEZ27 | Leucosome | 49.1 | 1.40 | 2s | 378.27 | Peña-Alonso et al. (2017) |
| PEZ27 | Leucosome | 56.0 | 1.80 | 2s | 378.27 | Peña-Alonso et al. (2017) |
| PEZ27 | Leucosome | 55.3 | 2.20 | 2s | 378.27 | Peña-Alonso et al. (2017) |
| PEZ27 | Leucosome | 56.6 | 1.60 | 2s | 378.27 | Peña-Alonso et al. (2017) |
| PEZ27 | Leucosome | 57.7 | 2.20 | 2s | 378.27 | Peña-Alonso et al. (2017) |
| PEZ27 | Leucosome | 57.5 | 1.90 | 2s | 378.27 | Peña-Alonso et al. (2017) |
| PEZ27 | Leucosome | 55.4 | 1.50 | 2s | 378.27 | Peña-Alonso et al. (2017) |
| PEZ27 | Leucosome | 57.0 | 1.80 | 2s | 378.27 | Peña-Alonso et al. (2017) |
| PEZ27 | Leucosome | 55.3 | 1.50 | 2s | 378.27 | Peña-Alonso et al. (2017) |
| PEZ27 | Leucosome | 58.2 | 2.00 | 2s | 378.27 | Peña-Alonso et al. (2017) |
| PEZ27 | Leucosome | 55.9 | 1.60 | 2s | 378.27 | Peña-Alonso et al. (2017) |
| PEZ27 | Leucosome | 163.5 | 3.80 | 2s | 378.27 | Peña-Alonso et al. (2017) |
| PEZ27 | Leucosome | 56.7 | 1.60 | 2s | 378.27 | Peña-Alonso et al. (2017) |
| PEZ27 | Leucosome | 56.1 | 1.50 | 2s | 378.27 | Peña-Alonso et al. (2017) |
| PEZ27 | Leucosome | 56.2 | 1.40 | 2s | 378.27 | Peña-Alonso et al. (2017) |
| PEZ27 | Leucosome | 57.0 | 2.40 | 2s | 378.27 | Peña-Alonso et al. (2017) |
| PEZ27 | Leucosome | 57.3 | 2.00 | 2s | 378.27 | Peña-Alonso et al. (2017) |
| PEZ27 | Leucosome | 55.2 | 2.30 | 2s | 378.27 | Peña-Alonso et al. (2017) |
| PEZ27 | Leucosome | 54.6 | 1.50 | 2s | 378.27 | Peña-Alonso et al. (2017) |
| PEZ27 | Leucosome | 57.6 | 1.70 | 2s | 378.27 | Peña-Alonso et al. (2017) |
| PEZ27 | Leucosome | 57.4 | 1.80 | 2s | 378.27 | Peña-Alonso et al. (2017) |
| PEZ27 | Leucosome | 57.3 | 1.60 | 2s | 378.27 | Peña-Alonso et al. (2017) |
| PEZ27 | Leucosome | 57.7 | 2.00 | 2s | 378.27 | Peña-Alonso et al. (2017) |
| PEZ27 | Leucosome | 50.4 | 1.50 | 2s | 378.27 | Peña-Alonso et al. (2017) |
| PEZ27 | Leucosome | 57.2 | 1.70 | 2s | 378.27 | Peña-Alonso et al. (2017) |
| PEZ27 | Leucosome | 57.7 | 2.10 | 2s | 378.27 | Peña-Alonso et al. (2017) |
| PEZ43 | Leucosome | 155.9 | 3.50 | 2s | 399.21 | Peña-Alonso et al. (2017) |
| PEZ43 | Leucosome | 161.7 | 3.80 | 2s | 399.21 | Peña-Alonso et al. (2017) |
| PEZ43 | Leucosome | 107.7 | 7.40 | 2s | 399.21 | Peña-Alonso et al. (2017) |
| PEZ43 | Leucosome | 162.9 | 3.80 | 2s | 399.21 | Peña-Alonso et al. (2017) |
| PEZ43 | Leucosome | 793.0 | 21.00 | 2s | 399.21 | Peña-Alonso et al. (2017) |
| PEZ43 | Leucosome | 1123.0 | 27.00 | 2s | 399.21 | Peña-Alonso et al. (2017) |
| PEZ43 | Leucosome | 155.6 | 3.70 | 2s | 399.21 | Peña-Alonso et al. (2017) |
| PEZ43 | Leucosome | 1142.0 | 21.00 | 2s | 399.21 | Peña-Alonso et al. (2017) |
| PEZ43 | Leucosome | 161.6 | 4.80 | 2s | 399.21 | Peña-Alonso et al. (2017) |
| PEZ43 | Leucosome | 170.8 | 4.40 | 2s | 399.21 | Peña-Alonso et al. (2017) |
| PEZ43 | Leucosome | 413.0 | 18.00 | 2s | 399.21 | Peña-Alonso et al. (2017) |
| PEZ43 | Leucosome | 154.2 | 3.60 | 2s | 399.21 | Peña-Alonso et al. (2017) |
| PEZ43 | Leucosome | 161.4 | 3.90 | 2s | 399.21 | Peña-Alonso et al. (2017) |
| PEZ43 | Leucosome | 156.0 | 11.00 | 2s | 399.21 | Peña-Alonso et al. (2017) |
| PEZ43 | Leucosome | 155.1 | 9.40 | 2s | 399.21 | Peña-Alonso et al. (2017) |
| PEZ43 | Leucosome | 212.5 | 5.10 | 2s | 399.21 | Peña-Alonso et al. (2017) |
| PEZ43 | Leucosome | 1488.0 | 49.00 | 2s | 399.21 | Peña-Alonso et al. (2017) |
| PEZ43 | Leucosome | 56.0 | 1.50 | 2s | 399.21 | Peña-Alonso et al. (2017) |
| PEZ43 | Leucosome | 167.6 | 3.90 | 2s | 399.21 | Peña-Alonso et al. (2017) |
| PEZ43 | Leucosome | 157.8 | 4.40 | 2s | 399.21 | Peña-Alonso et al. (2017) |
| PEZ43 | Leucosome | 155.6 | 3.60 | 2s | 399.21 | Peña-Alonso et al. (2017) |
| PEZ43 | Leucosome | 1362.0 | 27.00 | 2s | 399.21 | Peña-Alonso et al. (2017) |
| PEZ43 | Leucosome | 149.8 | 4.10 | 2s | 399.21 | Peña-Alonso et al. (2017) |
| PEZ43 | Leucosome | 162.5 | 3.70 | 2s | 399.21 | Peña-Alonso et al. (2017) |
| PEZ43 | Leucosome | 1181.0 | 22.00 | 2s | 399.21 | Peña-Alonso et al. (2017) |
| PEZ43 | Leucosome | 482.0 | 27.00 | 2s | 399.21 | Peña-Alonso et al. (2017) |
| PEZ43 | Leucosome | 169.9 | 4.60 | 2s | 399.21 | Peña-Alonso et al. (2017) |
| PEZ43 | Leucosome | 163.8 | 4.10 | 2s | 399.21 | Peña-Alonso et al. (2017) |
| PEZ43 | Leucosome | 159.9 | 3.90 | 2s | 399.21 | Peña-Alonso et al. (2017) |
| PEZ43 | Leucosome | 161.4 | 3.70 | 2s | 399.21 | Peña-Alonso et al. (2017) |
| PEZ43 | Leucosome | 161.9 | 3.80 | 2s | 399.21 | Peña-Alonso et al. (2017) |
| PEZ43 | Leucosome | 156.8 | 3.70 | 2s | 399.21 | Peña-Alonso et al. (2017) |
| PEZ43 | Leucosome | 158.6 | 4.30 | 2s | 399.21 | Peña-Alonso et al. (2017) |
| PEZ43 | Leucosome | 162.8 | 3.80 | 2s | 399.21 | Peña-Alonso et al. (2017) |
| PEZ43 | Leucosome | 184.8 | 4.20 | 2s | 399.21 | Peña-Alonso et al. (2017) |
| PEZ43 | Leucosome | 163.0 | 3.90 | 2s | 399.21 | Peña-Alonso et al. (2017) |
| PEZ43 | Leucosome | 178.4 | 4.90 | 2s | 399.21 | Peña-Alonso et al. (2017) |
| PEZ43 | Leucosome | 159.5 | 3.90 | 2s | 399.21 | Peña-Alonso et al. (2017) |
| PEZ43 | Leucosome | 163.8 | 3.90 | 2s | 399.21 | Peña-Alonso et al. (2017) |
| PEZ43 | Leucosome | 177.2 | 5.20 | 2s | 399.21 | Peña-Alonso et al. (2017) |
| PEZ43 | Leucosome | 162.7 | 3.90 | 2s | 399.21 | Peña-Alonso et al. (2017) |
| PEZ43 | Leucosome | 159.6 | 4.20 | 2s | 399.21 | Peña-Alonso et al. (2017) |
| PEZ43 | Leucosome | 1075.0 | 44.00 | 2s | 399.21 | Peña-Alonso et al. (2017) |
| PEZ43 | Leucosome | 159.7 | 3.70 | 2s | 399.21 | Peña-Alonso et al. (2017) |
| PEZ43 | Leucosome | 1153.0 | 20.00 | 2s | 399.21 | Peña-Alonso et al. (2017) |
| PEZ43 | Leucosome | 144.1 | 7.40 | 2s | 399.21 | Peña-Alonso et al. (2017) |
| PEZ43 | Leucosome | 159.1 | 3.60 | 2s | 399.21 | Peña-Alonso et al. (2017) |
| PEZ43 | Leucosome | 707.0 | 18.00 | 2s | 399.21 | Peña-Alonso et al. (2017) |
| PEZ43 | Leucosome | 169.9 | 4.20 | 2s | 399.21 | Peña-Alonso et al. (2017) |
| PEZ43 | Leucosome | 916.0 | 32.00 | 2s | 399.21 | Peña-Alonso et al. (2017) |
| Ac0707 | Intrusive | 50.2 | 0.60 | 1s | 65.57 | Hernández-Pineda et al. (2011) |
| Ac0707 | Intrusive | 49.8 | 0.40 | 1s | 65.57 | Hernández-Pineda et al. (2011) |
| Ac0707 | Intrusive | 51.8 | 0.60 | 1s | 65.57 | Hernández-Pineda et al. (2011) |
| Ac0707 | Intrusive | 50.0 | 0.60 | 1s | 65.57 | Hernández-Pineda et al. (2011) |
| Ac0707 | Intrusive | 50.9 | 0.50 | 1s | 65.57 | Hernández-Pineda et al. (2011) |
| Ac0707 | Intrusive | 51.4 | 0.50 | 1s | 65.57 | Hernández-Pineda et al. (2011) |
| Ac0707 | Intrusive | 50.0 | 0.60 | 1s | 65.57 | Hernández-Pineda et al. (2011) |
| Ac0707 | Intrusive | 51.9 | 0.50 | 1s | 65.57 | Hernández-Pineda et al. (2011) |
| Ac0707 | Intrusive | 52.1 | 0.50 | 1s | 65.57 | Hernández-Pineda et al. (2011) |
| Ac0707 | Intrusive | 49.6 | 0.60 | 1s | 65.57 | Hernández-Pineda et al. (2011) |
| Ac0707 | Intrusive | 49.8 | 0.50 | 1s | 65.57 | Hernández-Pineda et al. (2011) |
| Ac0707 | Intrusive | 48.4 | 0.60 | 1s | 65.57 | Hernández-Pineda et al. (2011) |
| Ac0707 | Intrusive | 49.3 | 0.70 | 1s | 65.57 | Hernández-Pineda et al. (2011) |
| Ac0707 | Intrusive | 51.4 | 0.60 | 1s | 65.57 | Hernández-Pineda et al. (2011) |
| Ac0707 | Intrusive | 49.9 | 0.60 | 1s | 65.57 | Hernández-Pineda et al. (2011) |
| Ac0707 | Intrusive | 50.5 | 0.70 | 1s | 65.57 | Hernández-Pineda et al. (2011) |
| Ac0707 | Intrusive | 50.6 | 0.80 | 1s | 65.57 | Hernández-Pineda et al. (2011) |
| Ac0722 | Intrusive | 50.1 | 0.60 | 1s | 71.30 | Hernández-Pineda et al. (2011) |
| Ac0722 | Intrusive | 49.2 | 0.50 | 1s | 71.30 | Hernández-Pineda et al. (2011) |
| Ac0722 | Intrusive | 50.5 | 0.50 | 1s | 71.30 | Hernández-Pineda et al. (2011) |
| Ac0722 | Intrusive | 51.1 | 0.50 | 1s | 71.30 | Hernández-Pineda et al. (2011) |
| Ac0722 | Intrusive | 50.3 | 0.60 | 1s | 71.30 | Hernández-Pineda et al. (2011) |
| Ac0722 | Intrusive | 51.7 | 0.70 | 1s | 71.30 | Hernández-Pineda et al. (2011) |
| Ac0722 | Intrusive | 50.6 | 0.60 | 1s | 71.30 | Hernández-Pineda et al. (2011) |
| Ac0722 | Intrusive | 49.1 | 0.60 | 1s | 71.30 | Hernández-Pineda et al. (2011) |
| Ac0722 | Intrusive | 51.3 | 0.60 | 1s | 71.30 | Hernández-Pineda et al. (2011) |
| Ac0722 | Intrusive | 52.1 | 0.70 | 1s | 71.30 | Hernández-Pineda et al. (2011) |
| Ac0722 | Intrusive | 49.4 | 0.60 | 1s | 71.30 | Hernández-Pineda et al. (2011) |
| Ac0722 | Intrusive | 49.9 | 0.90 | 1s | 71.30 | Hernández-Pineda et al. (2011) |
| Ac0722 | Intrusive | 51.0 | 1.00 | 1s | 71.30 | Hernández-Pineda et al. (2011) |
| Ac0722 | Intrusive | 51.0 | 0.60 | 1s | 71.30 | Hernández-Pineda et al. (2011) |
| Ac0722 | Intrusive | 52.1 | 0.80 | 1s | 71.30 | Hernández-Pineda et al. (2011) |
| Ac0722 | Intrusive | 51.5 | 0.90 | 1s | 71.30 | Hernández-Pineda et al. (2011) |
| Ac0722 | Intrusive | 50.3 | 0.70 | 1s | 71.30 | Hernández-Pineda et al. (2011) |
| Ac0722 | Intrusive | 50.0 | 0.70 | 1s | 71.30 | Hernández-Pineda et al. (2011) |
| Ac0722 | Intrusive | 49.3 | 0.70 | 1s | 71.30 | Hernández-Pineda et al. (2011) |
| Ac0722 | Intrusive | 50.4 | 0.60 | 1s | 71.30 | Hernández-Pineda et al. (2011) |
| Ac0702 | Intrusive | 52.4 | 0.70 | 1s | 73.97 | Hernández-Pineda et al. (2011) |
| Ac0702 | Intrusive | 48.0 | 0.70 | 1s | 73.97 | Hernández-Pineda et al. (2011) |
| Ac0702 | Intrusive | 51.5 | 0.50 | 1s | 73.97 | Hernández-Pineda et al. (2011) |
| Ac0702 | Intrusive | 50.5 | 0.70 | 1s | 73.97 | Hernández-Pineda et al. (2011) |
| Ac0702 | Intrusive | 50.4 | 0.60 | 1s | 73.97 | Hernández-Pineda et al. (2011) |
| Ac0702 | Intrusive | 50.0 | 0.60 | 1s | 73.97 | Hernández-Pineda et al. (2011) |
| Ac0702 | Intrusive | 52.9 | 0.50 | 1s | 73.97 | Hernández-Pineda et al. (2011) |
| Ac0702 | Intrusive | 49.1 | 0.50 | 1s | 73.97 | Hernández-Pineda et al. (2011) |
| Ac0702 | Intrusive | 48.5 | 0.50 | 1s | 73.97 | Hernández-Pineda et al. (2011) |
| Ac0702 | Intrusive | 48.7 | 0.50 | 1s | 73.97 | Hernández-Pineda et al. (2011) |
| Ac0702 | Intrusive | 52.3 | 0.50 | 1s | 73.97 | Hernández-Pineda et al. (2011) |
| Ac0702 | Intrusive | 50.0 | 0.50 | 1s | 73.97 | Hernández-Pineda et al. (2011) |
| Ac0702 | Intrusive | 52.1 | 0.50 | 1s | 73.97 | Hernández-Pineda et al. (2011) |
| Ac0702 | Intrusive | 49.1 | 0.50 | 1s | 73.97 | Hernández-Pineda et al. (2011) |
| Ac0702 | Intrusive | 48.4 | 0.70 | 1s | 73.97 | Hernández-Pineda et al. (2011) |
| Ac0702 | Intrusive | 53.5 | 0.70 | 1s | 73.97 | Hernández-Pineda et al. (2011) |
| Ac0702 | Intrusive | 48.9 | 0.50 | 1s | 73.97 | Hernández-Pineda et al. (2011) |
| Ac0702 | Intrusive | 49.5 | 0.40 | 1s | 73.97 | Hernández-Pineda et al. (2011) |
| Ac0702 | Intrusive | 49.4 | 0.40 | 1s | 73.97 | Hernández-Pineda et al. (2011) |
| Ac0702 | Intrusive | 51.5 | 0.70 | 1s | 73.97 | Hernández-Pineda et al. (2011) |
| Ac0705 | Intrusive | 49.4 | 0.50 | 1s | 74.79 | Hernández-Pineda et al. (2011) |
| Ac0705 | Intrusive | 48.9 | 0.40 | 1s | 74.79 | Hernández-Pineda et al. (2011) |
| Ac0705 | Intrusive | 49.4 | 0.70 | 1s | 74.79 | Hernández-Pineda et al. (2011) |
| Ac0705 | Intrusive | 49.2 | 0.40 | 1s | 74.79 | Hernández-Pineda et al. (2011) |
| Ac0705 | Intrusive | 50.0 | 0.40 | 1s | 74.79 | Hernández-Pineda et al. (2011) |
| Ac0705 | Intrusive | 49.0 | 0.50 | 1s | 74.79 | Hernández-Pineda et al. (2011) |
| Ac0705 | Intrusive | 49.8 | 0.40 | 1s | 74.79 | Hernández-Pineda et al. (2011) |
| Ac0705 | Intrusive | 51.1 | 0.50 | 1s | 74.79 | Hernández-Pineda et al. (2011) |
| Ac0705 | Intrusive | 50.3 | 0.60 | 1s | 74.79 | Hernández-Pineda et al. (2011) |
| Ac0705 | Intrusive | 50.9 | 0.50 | 1s | 74.79 | Hernández-Pineda et al. (2011) |
| Ac0705 | Intrusive | 48.9 | 0.60 | 1s | 74.79 | Hernández-Pineda et al. (2011) |
| Ac0705 | Intrusive | 48.4 | 0.60 | 1s | 74.79 | Hernández-Pineda et al. (2011) |
| Ac0705 | Intrusive | 50.7 | 0.40 | 1s | 74.79 | Hernández-Pineda et al. (2011) |
| Ac0705 | Intrusive | 49.8 | 0.50 | 1s | 74.79 | Hernández-Pineda et al. (2011) |
| Ac0705 | Intrusive | 49.0 | 0.50 | 1s | 74.79 | Hernández-Pineda et al. (2011) |
| Ac0705 | Intrusive | 49.8 | 0.70 | 1s | 74.79 | Hernández-Pineda et al. (2011) |
| Ac0705 | Intrusive | 49.8 | 0.50 | 1s | 74.79 | Hernández-Pineda et al. (2011) |
| Ac0705 | Intrusive | 48.3 | 0.50 | 1s | 74.79 | Hernández-Pineda et al. (2011) |
| Ac0705 | Intrusive | 48.4 | 0.50 | 1s | 74.79 | Hernández-Pineda et al. (2011) |
| Ac0705 | Intrusive | 49.0 | 0.60 | 1s | 74.79 | Hernández-Pineda et al. (2011) |
| Xo301 | Orthogneiss | 19.2 | 12.50 | 1s | 95.08 | Pérez-Gutierrez et al. (2009) |
| Xo301 | Orthogneiss | 60.0 | 3.50 | 1s | 95.08 | Pérez-Gutierrez et al. (2009) |
| Xo301 | Orthogneiss | 60.1 | 1.30 | 1s | 95.08 | Pérez-Gutierrez et al. (2009) |
| Xo301 | Orthogneiss | 58.0 | 4.90 | 1s | 95.08 | Pérez-Gutierrez et al. (2009) |
| Xo301 | Orthogneiss | 61.2 | 5.00 | 1s | 95.08 | Pérez-Gutierrez et al. (2009) |
| Xo301 | Orthogneiss | 68.5 | 4.10 | 1s | 95.08 | Pérez-Gutierrez et al. (2009) |
| Xo301 | Orthogneiss | 93.2 | 2.30 | 1s | 95.08 | Pérez-Gutierrez et al. (2009) |
| Xo301 | Orthogneiss | 107.2 | 0.60 | 1s | 95.08 | Pérez-Gutierrez et al. (2009) |
| Xo301 | Orthogneiss | 126.2 | 1.30 | 1s | 95.08 | Pérez-Gutierrez et al. (2009) |
| Xo301 | Orthogneiss | 128.4 | 0.80 | 1s | 95.08 | Pérez-Gutierrez et al. (2009) |
| Xo301 | Orthogneiss | 129.6 | 1.30 | 1s | 95.08 | Pérez-Gutierrez et al. (2009) |
| Xo301 | Orthogneiss | 129.5 | 0.90 | 1s | 95.08 | Pérez-Gutierrez et al. (2009) |
| Xo301 | Orthogneiss | 129.8 | 1.00 | 1s | 95.08 | Pérez-Gutierrez et al. (2009) |
| Xo301 | Orthogneiss | 131.3 | 0.70 | 1s | 95.08 | Pérez-Gutierrez et al. (2009) |
| Xo301 | Orthogneiss | 132.4 | 0.70 | 1s | 95.08 | Pérez-Gutierrez et al. (2009) |
| Xo301 | Orthogneiss | 133.3 | 0.60 | 1s | 95.08 | Pérez-Gutierrez et al. (2009) |
| Xo301 | Orthogneiss | 133.7 | 0.80 | 1s | 95.08 | Pérez-Gutierrez et al. (2009) |
| Xo301 | Orthogneiss | 133.8 | 1.10 | 1s | 95.08 | Pérez-Gutierrez et al. (2009) |
| Xo301 | Orthogneiss | 134.6 | 0.60 | 1s | 95.08 | Pérez-Gutierrez et al. (2009) |
| Xo301 | Orthogneiss | 135.5 | 1.00 | 1s | 95.08 | Pérez-Gutierrez et al. (2009) |
| Xo303 | Orthogneiss | 116.2 | 1.00 | 1s | 107.51 | Pérez-Gutierrez et al. (2009) |
| Xo303 | Orthogneiss | 133.0 | 2.60 | 1s | 107.51 | Pérez-Gutierrez et al. (2009) |
| Xo303 | Orthogneiss | 139.9 | 2.80 | 1s | 107.51 | Pérez-Gutierrez et al. (2009) |
| Xo303 | Orthogneiss | 161.3 | 1.30 | 1s | 107.51 | Pérez-Gutierrez et al. (2009) |
| Xo303 | Orthogneiss | 168.7 | 1.20 | 1s | 107.51 | Pérez-Gutierrez et al. (2009) |
| Xo303 | Orthogneiss | 172.1 | 1.20 | 1s | 107.51 | Pérez-Gutierrez et al. (2009) |
| Xo303 | Orthogneiss | 174.3 | 1.40 | 1s | 107.51 | Pérez-Gutierrez et al. (2009) |
| Xo303 | Orthogneiss | 174.9 | 1.20 | 1s | 107.51 | Pérez-Gutierrez et al. (2009) |
| Xo303 | Orthogneiss | 175.9 | 1.10 | 1s | 107.51 | Pérez-Gutierrez et al. (2009) |
| Xo303 | Orthogneiss | 176.4 | 1.20 | 1s | 107.51 | Pérez-Gutierrez et al. (2009) |
| Xo303 | Orthogneiss | 178.3 | 1.40 | 1s | 107.51 | Pérez-Gutierrez et al. (2009) |
| Xo303 | Orthogneiss | 178.5 | 0.70 | 1s | 107.51 | Pérez-Gutierrez et al. (2009) |
| Xo303 | Orthogneiss | 178.3 | 1.10 | 1s | 107.51 | Pérez-Gutierrez et al. (2009) |
| Xo303 | Orthogneiss | 179.3 | 1.00 | 1s | 107.51 | Pérez-Gutierrez et al. (2009) |
| Xo303 | Orthogneiss | 179.4 | 1.20 | 1s | 107.51 | Pérez-Gutierrez et al. (2009) |
| Xo303 | Orthogneiss | 179.4 | 0.90 | 1s | 107.51 | Pérez-Gutierrez et al. (2009) |
| MO-140 | Intrusive | 51.7 | 1.28 | 2s | 24.20 | Valencia et al. (2009) |
| MO-140 | Intrusive | 51.7 | 2.19 | 2s | 24.20 | Valencia et al. (2009) |
| MO-140 | Intrusive | 54.5 | 2.66 | 2s | 24.20 | Valencia et al. (2009) |
| MO-140 | Intrusive | 53.3 | 2.39 | 2s | 24.20 | Valencia et al. (2009) |
| MO-140 | Intrusive | 50.5 | 3.39 | 2s | 24.20 | Valencia et al. (2009) |
| MO-140 | Intrusive | 49.3 | 3.38 | 2s | 24.20 | Valencia et al. (2009) |
| MO-140 | Intrusive | 51.7 | 9.76 | 2s | 24.20 | Valencia et al. (2009) |
| MO-140 | Intrusive | 56.2 | 5.02 | 2s | 24.20 | Valencia et al. (2009) |
| MO-140 | Intrusive | 50.2 | 7.36 | 2s | 24.20 | Valencia et al. (2009) |
| MO-140 | Intrusive | 56.2 | 2.69 | 2s | 24.20 | Valencia et al. (2009) |
| MO-140 | Intrusive | 51.2 | 4.59 | 2s | 24.20 | Valencia et al. (2009) |
| MO-140 | Intrusive | 50.1 | 2.68 | 2s | 24.20 | Valencia et al. (2009) |
| MO-140 | Intrusive | 55.6 | 3.40 | 2s | 24.20 | Valencia et al. (2009) |
| MO-140 | Intrusive | 54.8 | 3.36 | 2s | 24.20 | Valencia et al. (2009) |
| MO-140 | Intrusive | 54.3 | 3.20 | 2s | 24.20 | Valencia et al. (2009) |
| MO-140 | Intrusive | 56.8 | 1.76 | 2s | 24.20 | Valencia et al. (2009) |
| MO-140 | Intrusive | 56.6 | 3.66 | 2s | 24.20 | Valencia et al. (2009) |
| MO-140 | Intrusive | 53.4 | 5.23 | 2s | 24.20 | Valencia et al. (2009) |
| MO-140 | Intrusive | 53.9 | 3.65 | 2s | 24.20 | Valencia et al. (2009) |
| MO-140 | Intrusive | 54.7 | 6.33 | 2s | 24.20 | Valencia et al. (2009) |
| MO-140 | Intrusive | 55.1 | 2.21 | 2s | 24.20 | Valencia et al. (2009) |
| MO-140 | Intrusive | 55.1 | 6.22 | 2s | 24.20 | Valencia et al. (2009) |
| MO-141 | Intrusive | 53.2 | 1.71 | 2s | 30.93 | Valencia et al. (2009) |
| MO-141 | Intrusive | 50.9 | 3.18 | 2s | 30.93 | Valencia et al. (2009) |
| MO-141 | Intrusive | 54.5 | 2.78 | 2s | 30.93 | Valencia et al. (2009) |
| MO-141 | Intrusive | 49.9 | 10.36 | 2s | 30.93 | Valencia et al. (2009) |
| MO-141 | Intrusive | 52.7 | 2.27 | 2s | 30.93 | Valencia et al. (2009) |
| MO-141 | Intrusive | 51.4 | 1.43 | 2s | 30.93 | Valencia et al. (2009) |
| MO-141 | Intrusive | 56.3 | 1.84 | 2s | 30.93 | Valencia et al. (2009) |
| MO-141 | Intrusive | 51.8 | 1.74 | 2s | 30.93 | Valencia et al. (2009) |
| MO-141 | Intrusive | 61.1 | 12.57 | 2s | 30.93 | Valencia et al. (2009) |
| MO-141 | Intrusive | 51.1 | 1.43 | 2s | 30.93 | Valencia et al. (2009) |
| MO-141 | Intrusive | 49.8 | 1.80 | 2s | 30.93 | Valencia et al. (2009) |
| MO-141 | Intrusive | 53.3 | 4.58 | 2s | 30.93 | Valencia et al. (2009) |
| MO-141 | Intrusive | 325.9 | 22.85 | 2s | 30.93 | Valencia et al. (2009) |
| MO-141 | Intrusive | 53.9 | 3.13 | 2s | 30.93 | Valencia et al. (2009) |
| MO-141 | Intrusive | 58.6 | 3.23 | 2s | 30.93 | Valencia et al. (2009) |
| MO-141 | Intrusive | 54.3 | 3.77 | 2s | 30.93 | Valencia et al. (2009) |
| MO-141 | Intrusive | 56.4 | 5.80 | 2s | 30.93 | Valencia et al. (2009) |
| MO-141 | Intrusive | 57.2 | 2.97 | 2s | 30.93 | Valencia et al. (2009) |
| MO-141 | Intrusive | 52.4 | 1.55 | 2s | 30.93 | Valencia et al. (2009) |
| MO-141 | Intrusive | 58.1 | 4.26 | 2s | 30.93 | Valencia et al. (2009) |
| MO-141 | Intrusive | 48.7 | 3.95 | 2s | 30.93 | Valencia et al. (2009) |
| MO-142 | Intrusive | 57.8 | 4.51 | 2s | 51.63 | Valencia et al. (2009) |
| MO-142 | Intrusive | 53.9 | 3.24 | 2s | 51.63 | Valencia et al. (2009) |
| MO-142 | Intrusive | 56.5 | 3.67 | 2s | 51.63 | Valencia et al. (2009) |
| MO-142 | Intrusive | 72.3 | 4.03 | 2s | 51.63 | Valencia et al. (2009) |
| MO-142 | Intrusive | 58.2 | 4.38 | 2s | 51.63 | Valencia et al. (2009) |
| MO-142 | Intrusive | 61.1 | 2.65 | 2s | 51.63 | Valencia et al. (2009) |
| MO-142 | Intrusive | 385.6 | 17.30 | 2s | 51.63 | Valencia et al. (2009) |
| MO-142 | Intrusive | 62.3 | 4.42 | 2s | 51.63 | Valencia et al. (2009) |
| MO-142 | Intrusive | 63.2 | 3.29 | 2s | 51.63 | Valencia et al. (2009) |
| MO-142 | Intrusive | 57.5 | 3.31 | 2s | 51.63 | Valencia et al. (2009) |
| MO-142 | Intrusive | 90.1 | 7.14 | 2s | 51.63 | Valencia et al. (2009) |
| MO-142 | Intrusive | 65.1 | 1.12 | 2s | 51.63 | Valencia et al. (2009) |
| MO-142 | Intrusive | 64.3 | 3.05 | 2s | 51.63 | Valencia et al. (2009) |
| MO-142 | Intrusive | 71.2 | 7.75 | 2s | 51.63 | Valencia et al. (2009) |
| MO-142 | Intrusive | 65.9 | 7.42 | 2s | 51.63 | Valencia et al. (2009) |
| MO-142 | Intrusive | 68.6 | 7.34 | 2s | 51.63 | Valencia et al. (2009) |
| MO-142 | Intrusive | 57.8 | 1.09 | 2s | 51.63 | Valencia et al. (2009) |
| MO-142 | Intrusive | 56.7 | 3.94 | 2s | 51.63 | Valencia et al. (2009) |
| MO-142 | Intrusive | 54.3 | 4.70 | 2s | 51.63 | Valencia et al. (2009) |
| MO-142 | Intrusive | 92.1 | 10.46 | 2s | 51.63 | Valencia et al. (2009) |
| MO-142 | Intrusive | 63.4 | 5.62 | 2s | 51.63 | Valencia et al. (2009) |
| MO-142 | Intrusive | 73.8 | 2.68 | 2s | 51.63 | Valencia et al. (2009) |
| MO-142 | Intrusive | 326.2 | 14.17 | 2s | 51.63 | Valencia et al. (2009) |
| MO-143 | Intrusive | 55.2 | 1.53 | 2s | 59.75 | Valencia et al. (2009) |
| MO-143 | Intrusive | 55.1 | 3.72 | 2s | 59.75 | Valencia et al. (2009) |
| MO-143 | Intrusive | 54.1 | 2.24 | 2s | 59.75 | Valencia et al. (2009) |
| MO-143 | Intrusive | 55.0 | 2.69 | 2s | 59.75 | Valencia et al. (2009) |
| MO-143 | Intrusive | 52.6 | 5.78 | 2s | 59.75 | Valencia et al. (2009) |
| MO-143 | Intrusive | 53.1 | 1.42 | 2s | 59.75 | Valencia et al. (2009) |
| MO-143 | Intrusive | 53.4 | 5.66 | 2s | 59.75 | Valencia et al. (2009) |
| MO-143 | Intrusive | 53.3 | 3.59 | 2s | 59.75 | Valencia et al. (2009) |
| MO-143 | Intrusive | 147.0 | 13.47 | 2s | 59.75 | Valencia et al. (2009) |
| MO-143 | Intrusive | 54.1 | 1.06 | 2s | 59.75 | Valencia et al. (2009) |
| MO-143 | Intrusive | 53.4 | 2.07 | 2s | 59.75 | Valencia et al. (2009) |
| MO-143 | Intrusive | 53.6 | 1.66 | 2s | 59.75 | Valencia et al. (2009) |
| MO-143 | Intrusive | 102.4 | 6.62 | 2s | 59.75 | Valencia et al. (2009) |
| MO-143 | Intrusive | 142.9 | 7.85 | 2s | 59.75 | Valencia et al. (2009) |
| MO-143 | Intrusive | 106.2 | 6.39 | 2s | 59.75 | Valencia et al. (2009) |
| MO-143 | Intrusive | 56.3 | 2.23 | 2s | 59.75 | Valencia et al. (2009) |
| MO-143 | Intrusive | 155.7 | 12.11 | 2s | 59.75 | Valencia et al. (2009) |
| MO-143 | Intrusive | 56.7 | 1.68 | 2s | 59.75 | Valencia et al. (2009) |
| MO-143 | Intrusive | 53.4 | 2.39 | 2s | 59.75 | Valencia et al. (2009) |
| MO-143 | Intrusive | 56.2 | 4.87 | 2s | 59.75 | Valencia et al. (2009) |
| MO-143 | Intrusive | 104.3 | 3.58 | 2s | 59.75 | Valencia et al. (2009) |
| MO-143 | Intrusive | 960.4 | 48.04 | 2s | 59.75 | Valencia et al. (2009) |
| MO-143 | Intrusive | 53.7 | 3.05 | 2s | 59.75 | Valencia et al. (2009) |
| MO-143 | Intrusive | 54.8 | 2.86 | 2s | 59.75 | Valencia et al. (2009) |
| MO-144 | Intrusive | 87.6 | 3.36 | 2s | 63.20 | Valencia et al. (2009) |
| MO-144 | Intrusive | 61.1 | 2.49 | 2s | 63.20 | Valencia et al. (2009) |
| MO-144 | Intrusive | 55.7 | 1.84 | 2s | 63.20 | Valencia et al. (2009) |
| MO-144 | Intrusive | 61.7 | 1.81 | 2s | 63.20 | Valencia et al. (2009) |
| MO-144 | Intrusive | 57.1 | 1.12 | 2s | 63.20 | Valencia et al. (2009) |
| MO-144 | Intrusive | 65.6 | 9.23 | 2s | 63.20 | Valencia et al. (2009) |
| MO-144 | Intrusive | 60.8 | 3.40 | 2s | 63.20 | Valencia et al. (2009) |
| MO-144 | Intrusive | 60.3 | 3.27 | 2s | 63.20 | Valencia et al. (2009) |
| MO-144 | Intrusive | 87.9 | 3.76 | 2s | 63.20 | Valencia et al. (2009) |
| MO-144 | Intrusive | 56.6 | 3.90 | 2s | 63.20 | Valencia et al. (2009) |
| MO-144 | Intrusive | 57.9 | 2.45 | 2s | 63.20 | Valencia et al. (2009) |
| MO-144 | Intrusive | 87.1 | 2.84 | 2s | 63.20 | Valencia et al. (2009) |
| MO-144 | Intrusive | 57.2 | 2.08 | 2s | 63.20 | Valencia et al. (2009) |
| MO-144 | Intrusive | 82.7 | 5.87 | 2s | 63.20 | Valencia et al. (2009) |
| MO-144 | Intrusive | 56.7 | 1.76 | 2s | 63.20 | Valencia et al. (2009) |
| MO-144 | Intrusive | 55.3 | 4.07 | 2s | 63.20 | Valencia et al. (2009) |
| MO-144 | Intrusive | 58.1 | 2.08 | 2s | 63.20 | Valencia et al. (2009) |
| MO-144 | Intrusive | 56.6 | 2.95 | 2s | 63.20 | Valencia et al. (2009) |
| MO-145 | Intrusive | 64.8 | 7.71 | 2s | 20.63 | Valencia et al. (2009) |
| MO-145 | Intrusive | 36.8 | 3.16 | 2s | 20.63 | Valencia et al. (2009) |
| MO-145 | Intrusive | 38.7 | 5.98 | 2s | 20.63 | Valencia et al. (2009) |
| MO-145 | Intrusive | 36.9 | 2.16 | 2s | 20.63 | Valencia et al. (2009) |
| MO-145 | Intrusive | 38.7 | 6.30 | 2s | 20.63 | Valencia et al. (2009) |
| MO-145 | Intrusive | 131.4 | 7.48 | 2s | 20.63 | Valencia et al. (2009) |
| MO-145 | Intrusive | 64.4 | 10.01 | 2s | 20.63 | Valencia et al. (2009) |
| MO-145 | Intrusive | 62.5 | 9.47 | 2s | 20.63 | Valencia et al. (2009) |
| MO-145 | Intrusive | 59.6 | 6.43 | 2s | 20.63 | Valencia et al. (2009) |
| MO-145 | Intrusive | 43.0 | 2.53 | 2s | 20.63 | Valencia et al. (2009) |
| MO-145 | Intrusive | 38.8 | 7.25 | 2s | 20.63 | Valencia et al. (2009) |
| MO-145 | Intrusive | 967.2 | 12.73 | 2s | 20.63 | Valencia et al. (2009) |
| MO-145 | Intrusive | 38.3 | 3.94 | 2s | 20.63 | Valencia et al. (2009) |
| MO-145 | Intrusive | 39.3 | 2.65 | 2s | 20.63 | Valencia et al. (2009) |
| MO-145 | Intrusive | 35.9 | 7.33 | 2s | 20.63 | Valencia et al. (2009) |
| MO-145 | Intrusive | 39.6 | 8.32 | 2s | 20.63 | Valencia et al. (2009) |
| MO-145 | Intrusive | 103.3 | 8.61 | 2s | 20.63 | Valencia et al. (2009) |
| MO-145 | Intrusive | 58.0 | 3.11 | 2s | 20.63 | Valencia et al. (2009) |
| MO-145 | Intrusive | 42.4 | 3.89 | 2s | 20.63 | Valencia et al. (2009) |
| MO-145 | Intrusive | 57.7 | 2.93 | 2s | 20.63 | Valencia et al. (2009) |
| MO-145 | Intrusive | 41.4 | 2.68 | 2s | 20.63 | Valencia et al. (2009) |
| MO-145 | Intrusive | 41.5 | 1.37 | 2s | 20.63 | Valencia et al. (2009) |
| PEZ03C | Leucosome | 25.6 | 0.77 | 2s | 386.55 | Latorre (2018) |
| PEZ03C | Leucosome | 26.2 | 0.67 | 2s | 386.55 | Latorre (2018) |
| PEZ03C | Leucosome | 335.6 | 4.80 | 2s | 386.55 | Latorre (2018) |
| PEZ03C | Leucosome | 28.8 | 0.80 | 2s | 386.55 | Latorre (2018) |
| PEZ03C | Leucosome | 42.0 | 1.30 | 2s | 386.55 | Latorre (2018) |
| PEZ03C | Leucosome | 30.3 | 0.78 | 2s | 386.55 | Latorre (2018) |
| PEZ03C | Leucosome | 30.6 | 0.81 | 2s | 386.55 | Latorre (2018) |
| PEZ03C | Leucosome | 31.0 | 0.60 | 2s | 386.55 | Latorre (2018) |
| PEZ03C | Leucosome | 25.9 | 0.58 | 2s | 386.55 | Latorre (2018) |
| PEZ03C | Leucosome | 29.8 | 0.65 | 2s | 386.55 | Latorre (2018) |
| PEZ03C | Leucosome | 30.7 | 0.56 | 2s | 386.55 | Latorre (2018) |
| PEZ03C | Leucosome | 27.5 | 0.73 | 2s | 386.55 | Latorre (2018) |
| PEZ03C | Leucosome | 28.6 | 0.72 | 2s | 386.55 | Latorre (2018) |
| PEZ03C | Leucosome | 62.8 | 1.20 | 2s | 386.55 | Latorre (2018) |
| PEZ03C | Leucosome | 25.1 | 0.74 | 2s | 386.55 | Latorre (2018) |
| PEZ03C | Leucosome | 32.5 | 1.00 | 2s | 386.55 | Latorre (2018) |
| PEZ03C | Leucosome | 53.6 | 0.77 | 2s | 386.55 | Latorre (2018) |
| PEZ03C | Leucosome | 30.0 | 0.64 | 2s | 386.55 | Latorre (2018) |
| PEZ03C | Leucosome | 30.5 | 0.59 | 2s | 386.55 | Latorre (2018) |
| PEZ03C | Leucosome | 30.8 | 0.57 | 2s | 386.55 | Latorre (2018) |
| PEZ03C | Leucosome | 30.4 | 0.83 | 2s | 386.55 | Latorre (2018) |
| PEZ03E | Paleosome | 257.6 | 8.50 | 2s | 386.55 | Latorre (2018) |
| PEZ03E | Paleosome | 443.0 | 7.30 | 2s | 386.55 | Latorre (2018) |
| PEZ03E | Paleosome | 426.1 | 6.10 | 2s | 386.55 | Latorre (2018) |
| PEZ03E | Paleosome | 230.1 | 5.10 | 2s | 386.55 | Latorre (2018) |
| PEZ03E | Paleosome | 395.4 | 5.60 | 2s | 386.55 | Latorre (2018) |
| PEZ03E | Paleosome | 47.1 | 1.50 | 2s | 386.55 | Latorre (2018) |
| PEZ03E | Paleosome | 169.3 | 4.20 | 2s | 386.55 | Latorre (2018) |
| PEZ03E | Paleosome | 693.0 | 12.00 | 2s | 386.55 | Latorre (2018) |
| PEZ03E | Paleosome | 970.0 | 12.00 | 2s | 386.55 | Latorre (2018) |
| PEZ03E | Paleosome | 446.1 | 8.80 | 2s | 386.55 | Latorre (2018) |
| PEZ03E | Paleosome | 434.9 | 7.80 | 2s | 386.55 | Latorre (2018) |
| PEZ03E | Paleosome | 481.2 | 7.20 | 2s | 386.55 | Latorre (2018) |
| PEZ03E | Paleosome | 967.0 | 14.00 | 2s | 386.55 | Latorre (2018) |
| PEZ03E | Paleosome | 274.2 | 4.50 | 2s | 386.55 | Latorre (2018) |
| PEZ03E | Paleosome | 359.1 | 5.50 | 2s | 386.55 | Latorre (2018) |
| PEZ03E | Paleosome | 166.7 | 4.30 | 2s | 386.55 | Latorre (2018) |
| PEZ03E | Paleosome | 169.1 | 5.00 | 2s | 386.55 | Latorre (2018) |
| PEZ03E | Paleosome | 766.0 | 10.00 | 2s | 386.55 | Latorre (2018) |
| PEZ03E | Paleosome | 65.2 | 2.70 | 2s | 386.55 | Latorre (2018) |
| PEZ03E | Paleosome | 589.0 | 10.00 | 2s | 386.55 | Latorre (2018) |
| PEZ03E | Paleosome | 104.6 | 2.00 | 2s | 386.55 | Latorre (2018) |
| M 01 46b | Hbl-syenite | 54.1 | 1.30 | 2s | 73.03 | Ducea et al. (2004) |
| M 01 46b | Hbl-syenite | 54.3 | 1.20 | 2s | 73.03 | Ducea et al. (2004) |
| M 01 46b | Hbl-syenite | 52.5 | 1.50 | 2s | 73.03 | Ducea et al. (2004) |
| M 01 46b | Hbl-syenite | 50.6 | 1.90 | 2s | 73.03 | Ducea et al. (2004) |
| M 01 46b | Hbl-syenite | 56.2 | 1.80 | 2s | 73.03 | Ducea et al. (2004) |
| M 01 46b | Hbl-syenite | 71.0 | 1.90 | 2s | 73.03 | Ducea et al. (2004) |
| M 01 46b | Hbl-syenite | 57.8 | 2.00 | 2s | 73.03 | Ducea et al. (2004) |
| M 01 46b | Hbl-syenite | 54.9 | 1.20 | 2s | 73.03 | Ducea et al. (2004) |
| M 01 46b | Hbl-syenite | 54.6 | 1.20 | 2s | 73.03 | Ducea et al. (2004) |
| M 01 46b | Hbl-syenite | 60.1 | 1.60 | 2s | 73.03 | Ducea et al. (2004) |
| M 01 46b | Hbl-syenite | 52.8 | 1.20 | 2s | 73.03 | Ducea et al. (2004) |
| M 01 46b | Hbl-syenite | 54.2 | 2.50 | 2s | 73.03 | Ducea et al. (2004) |
| M 01 46b | Hbl-syenite | 54.8 | 1.20 | 2s | 73.03 | Ducea et al. (2004) |
| M 01 46b | Hbl-syenite | 56.7 | 1.20 | 2s | 73.03 | Ducea et al. (2004) |
| M 01 46b | Hbl-syenite | 50.0 | 1.20 | 2s | 73.03 | Ducea et al. (2004) |
| M 01 46b | Hbl-syenite | 55.5 | 1.20 | 2s | 73.03 | Ducea et al. (2004) |
| M 01 46b | Hbl-syenite | 61.6 | 1.30 | 2s | 73.03 | Ducea et al. (2004) |
| M 01 46b | Hbl-syenite | 56.4 | 1.20 | 2s | 73.03 | Ducea et al. (2004) |
| M 01 46b | Hbl-syenite | 59.2 | 1.30 | 2s | 73.03 | Ducea et al. (2004) |
| M 01 46b | Hbl-syenite | 55.8 | 1.30 | 2s | 73.03 | Ducea et al. (2004) |
| M 01 46b | Hbl-syenite | 51.5 | 1.20 | 2s | 73.03 | Ducea et al. (2004) |
| M 01 46b | Hbl-syenite | 54.9 | 1.30 | 2s | 73.03 | Ducea et al. (2004) |
| M 01 46b | Hbl-syenite | 56.1 | 1.40 | 2s | 73.03 | Ducea et al. (2004) |
| M 01 46b | Hbl-syenite | 57.4 | 1.40 | 2s | 73.03 | Ducea et al. (2004) |
| M01-02 | Orthogneiss | 138.7 | 2.90 | 2s | 119.09 | Ducea et al. (2004) |
| M01-02 | Orthogneiss | 153.6 | 3.30 | 2s | 119.09 | Ducea et al. (2004) |
| M01-02 | Orthogneiss | 139.8 | 3.20 | 2s | 119.09 | Ducea et al. (2004) |
| M01-02 | Orthogneiss | 132.9 | 5.40 | 2s | 119.09 | Ducea et al. (2004) |
| M01-02 | Orthogneiss | 147.0 | 6.00 | 2s | 119.09 | Ducea et al. (2004) |
| M01-02 | Orthogneiss | 150.7 | 6.20 | 2s | 119.09 | Ducea et al. (2004) |
| M01-02 | Orthogneiss | 144.3 | 6.00 | 2s | 119.09 | Ducea et al. (2004) |
| M01-02 | Orthogneiss | 135.1 | 5.50 | 2s | 119.09 | Ducea et al. (2004) |
| M01-02 | Orthogneiss | 148.4 | 6.10 | 2s | 119.09 | Ducea et al. (2004) |
| M01-02 | Orthogneiss | 146.6 | 6.40 | 2s | 119.09 | Ducea et al. (2004) |
| M01-02 | Orthogneiss | 148.3 | 3.10 | 2s | 119.09 | Ducea et al. (2004) |
| M01-02 | Orthogneiss | 146.3 | 3.10 | 2s | 119.09 | Ducea et al. (2004) |
| M01-02 | Orthogneiss | 141.6 | 3.00 | 2s | 119.09 | Ducea et al. (2004) |
| M01-02 | Orthogneiss | 126.8 | 2.70 | 2s | 119.09 | Ducea et al. (2004) |
| M01-02 | Orthogneiss | 133.2 | 3.00 | 2s | 119.09 | Ducea et al. (2004) |
| M01-02 | Orthogneiss | 141.1 | 3.00 | 2s | 119.09 | Ducea et al. (2004) |
| M01-02 | Orthogneiss | 144.3 | 3.20 | 2s | 119.09 | Ducea et al. (2004) |
| M01-02 | Orthogneiss | 135.4 | 3.10 | 2s | 119.09 | Ducea et al. (2004) |
| M01-02 | Orthogneiss | 128.8 | 2.80 | 2s | 119.09 | Ducea et al. (2004) |
| M01-02 | Orthogneiss | 141.2 | 5.70 | 2s | 119.09 | Ducea et al. (2004) |
| M01-02 | Orthogneiss | 143.8 | 3.00 | 2s | 119.09 | Ducea et al. (2004) |
| M01-02 | Orthogneiss | 141.4 | 2.90 | 2s | 119.09 | Ducea et al. (2004) |
| M01-02 | Orthogneiss | 139.4 | 2.90 | 2s | 119.09 | Ducea et al. (2004) |
| M01-02 | Orthogneiss | 144.8 | 3.30 | 2s | 119.09 | Ducea et al. (2004) |
| M01-02 | Orthogneiss | 144.3 | 3.10 | 2s | 119.09 | Ducea et al. (2004) |
| M01-02 | Orthogneiss | 130.4 | 2.80 | 2s | 119.09 | Ducea et al. (2004) |
| M01-02 | Orthogneiss | 137.9 | 3.00 | 2s | 119.09 | Ducea et al. (2004) |
| M01-02 | Orthogneiss | 138.9 | 2.90 | 2s | 119.09 | Ducea et al. (2004) |
| M01-02 | Orthogneiss | 140.9 | 3.00 | 2s | 119.09 | Ducea et al. (2004) |
| M01-02 | Orthogneiss | 137.0 | 3.20 | 2s | 119.09 | Ducea et al. (2004) |
| M01-02 | Orthogneiss | 142.4 | 3.10 | 2s | 119.09 | Ducea et al. (2004) |
| M01-02 | Orthogneiss | 150.1 | 3.20 | 2s | 119.09 | Ducea et al. (2004) |
| M01-02 | Orthogneiss | 140.2 | 3.10 | 2s | 119.09 | Ducea et al. (2004) |
| M01-02 | Orthogneiss | 142.3 | 3.20 | 2s | 119.09 | Ducea et al. (2004) |
| M01-02 | Orthogneiss | 144.6 | 3.00 | 2s | 119.09 | Ducea et al. (2004) |
| M01-02 | Orthogneiss | 137.8 | 2.90 | 2s | 119.09 | Ducea et al. (2004) |
| M01-02 | Orthogneiss | 144.0 | 3.00 | 2s | 119.09 | Ducea et al. (2004) |
| M01-02 | Orthogneiss | 148.2 | 3.20 | 2s | 119.09 | Ducea et al. (2004) |
| M01-02 | Orthogneiss | 137.6 | 2.80 | 2s | 119.09 | Ducea et al. (2004) |
| M01-02 | Orthogneiss | 135.2 | 3.00 | 2s | 119.09 | Ducea et al. (2004) |
| M01-02 | Orthogneiss | 141.0 | 2.90 | 2s | 119.09 | Ducea et al. (2004) |
| M01-04 | Bt-granodio | 34.7 | 1.10 | 2s | 98.49 | Ducea et al. (2004) |
| M01-04 | Bt-granodio | 34.5 | 1.10 | 2s | 98.49 | Ducea et al. (2004) |
| M01-04 | Bt-granodio | 34.1 | 1.30 | 2s | 98.49 | Ducea et al. (2004) |
| M01-04 | Bt-granodio | 34.3 | 0.90 | 2s | 98.49 | Ducea et al. (2004) |
| M01-04 | Bt-granodio | 33.8 | 1.20 | 2s | 98.49 | Ducea et al. (2004) |
| M01-04 | Bt-granodio | 32.4 | 1.60 | 2s | 98.49 | Ducea et al. (2004) |
| M01-04 | Bt-granodio | 34.1 | 1.30 | 2s | 98.49 | Ducea et al. (2004) |
| M01-04 | Bt-granodio | 32.8 | 1.30 | 2s | 98.49 | Ducea et al. (2004) |
| M01-04 | Bt-granodio | 30.9 | 0.90 | 2s | 98.49 | Ducea et al. (2004) |
| M01-04 | Bt-granodio | 33.9 | 1.10 | 2s | 98.49 | Ducea et al. (2004) |
| M01-04 | Bt-granodio | 34.9 | 1.50 | 2s | 98.49 | Ducea et al. (2004) |
| M01-04 | Bt-granodio | 35.6 | 1.10 | 2s | 98.49 | Ducea et al. (2004) |
| M01-04 | Bt-granodio | 35.1 | 1.20 | 2s | 98.49 | Ducea et al. (2004) |
| M01-04 | Bt-granodio | 35.7 | 1.10 | 2s | 98.49 | Ducea et al. (2004) |
| M01-04 | Bt-granodio | 35.1 | 1.10 | 2s | 98.49 | Ducea et al. (2004) |
| M01-04 | Bt-granodio | 34.2 | 1.90 | 2s | 98.49 | Ducea et al. (2004) |
| M01-04 | Bt-granodio | 36.1 | 1.30 | 2s | 98.49 | Ducea et al. (2004) |
| M01-04 | Bt-granodio | 33.4 | 1.20 | 2s | 98.49 | Ducea et al. (2004) |
| M01-04 | Bt-granodio | 35.0 | 1.00 | 2s | 98.49 | Ducea et al. (2004) |
| M002re | Orthogneiss | 136.7 | 3.40 | 2s | 141.13 | Ducea et al. (2004) |
| M002re | Orthogneiss | 133.0 | 3.00 | 2s | 141.13 | Ducea et al. (2004) |
| M002re | Orthogneiss | 136.7 | 3.10 | 2s | 141.13 | Ducea et al. (2004) |
| M002re | Orthogneiss | 130.4 | 5.90 | 2s | 141.13 | Ducea et al. (2004) |
| M002re | Orthogneiss | 128.0 | 5.50 | 2s | 141.13 | Ducea et al. (2004) |
| M002re | Orthogneiss | 133.3 | 5.60 | 2s | 141.13 | Ducea et al. (2004) |
| M002re | Orthogneiss | 138.7 | 5.90 | 2s | 141.13 | Ducea et al. (2004) |
| M002re | Orthogneiss | 132.2 | 5.40 | 2s | 141.13 | Ducea et al. (2004) |
| M002re | Orthogneiss | 135.8 | 5.60 | 2s | 141.13 | Ducea et al. (2004) |
| M002re | Orthogneiss | 137.7 | 5.70 | 2s | 141.13 | Ducea et al. (2004) |
| M002re | Orthogneiss | 144.1 | 6.00 | 2s | 141.13 | Ducea et al. (2004) |
| M002re | Orthogneiss | 139.4 | 3.10 | 2s | 141.13 | Ducea et al. (2004) |
| M002re | Orthogneiss | 136.0 | 2.90 | 2s | 141.13 | Ducea et al. (2004) |
| M002re | Orthogneiss | 139.4 | 3.10 | 2s | 141.13 | Ducea et al. (2004) |
| M002re | Orthogneiss | 142.1 | 3.20 | 2s | 141.13 | Ducea et al. (2004) |
| M002re | Orthogneiss | 140.9 | 3.10 | 2s | 141.13 | Ducea et al. (2004) |
| M002re | Orthogneiss | 135.2 | 3.20 | 2s | 141.13 | Ducea et al. (2004) |
| M002re | Orthogneiss | 137.7 | 2.90 | 2s | 141.13 | Ducea et al. (2004) |
| M002re | Orthogneiss | 141.7 | 2.90 | 2s | 141.13 | Ducea et al. (2004) |
| M002re | Orthogneiss | 138.4 | 2.80 | 2s | 141.13 | Ducea et al. (2004) |
| M002re | Orthogneiss | 134.8 | 2.80 | 2s | 141.13 | Ducea et al. (2004) |
| M002re | Orthogneiss | 121.9 | 3.30 | 2s | 141.13 | Ducea et al. (2004) |
| M002re | Orthogneiss | 125.5 | 2.70 | 2s | 141.13 | Ducea et al. (2004) |
| M002re | Orthogneiss | 133.6 | 2.80 | 2s | 141.13 | Ducea et al. (2004) |
| M002re | Orthogneiss | 130.9 | 3.30 | 2s | 141.13 | Ducea et al. (2004) |
| M002re | Orthogneiss | 137.1 | 3.30 | 2s | 141.13 | Ducea et al. (2004) |
| M002re | Orthogneiss | 139.5 | 3.00 | 2s | 141.13 | Ducea et al. (2004) |
| M002re | Orthogneiss | 133.8 | 2.80 | 2s | 141.13 | Ducea et al. (2004) |
| M002re | Orthogneiss | 123.8 | 2.90 | 2s | 141.13 | Ducea et al. (2004) |
| M002re | Orthogneiss | 132.0 | 3.10 | 2s | 141.13 | Ducea et al. (2004) |
| M002re | Orthogneiss | 140.6 | 3.10 | 2s | 141.13 | Ducea et al. (2004) |
| M002re | Orthogneiss | 131.2 | 3.00 | 2s | 141.13 | Ducea et al. (2004) |
| M002re | Orthogneiss | 140.9 | 3.30 | 2s | 141.13 | Ducea et al. (2004) |
| M01-11 | Orthogneiss | 918.0 | 20.40 | 2s | 469.47 | Ducea et al. (2004) |
| M01-11 | Orthogneiss | 1008.6 | 24.80 | 2s | 469.47 | Ducea et al. (2004) |
| M01-11 | Orthogneiss | 1059.1 | 24.20 | 2s | 469.47 | Ducea et al. (2004) |
| M01-11 | Orthogneiss | 1040.7 | 23.40 | 2s | 469.47 | Ducea et al. (2004) |
| M01-11 | Orthogneiss | 1029.8 | 23.40 | 2s | 469.47 | Ducea et al. (2004) |
| M01-11 | Orthogneiss | 1134.7 | 27.20 | 2s | 469.47 | Ducea et al. (2004) |
| M01-11 | Orthogneiss | 1162.7 | 27.00 | 2s | 469.47 | Ducea et al. (2004) |
| M01-11 | Orthogneiss | 1078.4 | 24.80 | 2s | 469.47 | Ducea et al. (2004) |
| M01-11 | Orthogneiss | 1038.4 | 24.10 | 2s | 469.47 | Ducea et al. (2004) |
| M01-11 | Orthogneiss | 1049.8 | 23.90 | 2s | 469.47 | Ducea et al. (2004) |
| M01-11 | Orthogneiss | 1135.5 | 26.10 | 2s | 469.47 | Ducea et al. (2004) |
| M01-11 | Orthogneiss | 1145.4 | 26.30 | 2s | 469.47 | Ducea et al. (2004) |
| M01-11 | Orthogneiss | 1112.5 | 26.40 | 2s | 469.47 | Ducea et al. (2004) |
| M01-11 | Orthogneiss | 1119.2 | 26.80 | 2s | 469.47 | Ducea et al. (2004) |
| M01-11 | Orthogneiss | 1095.5 | 24.50 | 2s | 469.47 | Ducea et al. (2004) |
| M01-11 | Orthogneiss | 1114.5 | 25.30 | 2s | 469.47 | Ducea et al. (2004) |
| M01-11 | Orthogneiss | 1058.2 | 25.00 | 2s | 469.47 | Ducea et al. (2004) |
| M01-11 | Orthogneiss | 1091.8 | 24.70 | 2s | 469.47 | Ducea et al. (2004) |
| M01-11 | Orthogneiss | 1251.9 | 28.40 | 2s | 469.47 | Ducea et al. (2004) |
| M01-11 | Orthogneiss | 1041.9 | 23.30 | 2s | 469.47 | Ducea et al. (2004) |
| M01-11 | Orthogneiss | 1173.4 | 27.60 | 2s | 469.47 | Ducea et al. (2004) |
| M01-11 | Orthogneiss | 1132.6 | 31.80 | 2s | 469.47 | Ducea et al. (2004) |
| M01-11 | Orthogneiss | 1013.8 | 22.80 | 2s | 469.47 | Ducea et al. (2004) |
| M01-11 | Orthogneiss | 1084.1 | 25.10 | 2s | 469.47 | Ducea et al. (2004) |
| M01-11 | Orthogneiss | 1084.4 | 26.10 | 2s | 469.47 | Ducea et al. (2004) |
| M01-11 | Orthogneiss | 1050.7 | 26.50 | 2s | 469.47 | Ducea et al. (2004) |
| M01-11 | Orthogneiss | 1209.2 | 30.10 | 2s | 469.47 | Ducea et al. (2004) |
| M01-11 | Orthogneiss | 1031.8 | 25.20 | 2s | 469.47 | Ducea et al. (2004) |
| M01-11 | Orthogneiss | 1099.3 | 25.60 | 2s | 469.47 | Ducea et al. (2004) |
| M01-11 | Orthogneiss | 1155.8 | 28.60 | 2s | 469.47 | Ducea et al. (2004) |
| M01-14 | Orthogneiss | 1182.4 | 26.60 | 2s | 464.37 | Ducea et al. (2004) |
| M01-14 | Orthogneiss | 1172.0 | 26.60 | 2s | 464.37 | Ducea et al. (2004) |
| M01-14 | Orthogneiss | 1043.6 | 25.40 | 2s | 464.37 | Ducea et al. (2004) |
| M01-14 | Orthogneiss | 1053.0 | 23.30 | 2s | 464.37 | Ducea et al. (2004) |
| M01-14 | Orthogneiss | 1208.5 | 43.10 | 2s | 464.37 | Ducea et al. (2004) |
| M01-14 | Orthogneiss | 1105.6 | 24.80 | 2s | 464.37 | Ducea et al. (2004) |
| M01-14 | Orthogneiss | 1041.3 | 24.00 | 2s | 464.37 | Ducea et al. (2004) |
| M01-14 | Orthogneiss | 995.3 | 22.10 | 2s | 464.37 | Ducea et al. (2004) |
| M01-14 | Orthogneiss | 1231.5 | 29.70 | 2s | 464.37 | Ducea et al. (2004) |
| M01-14 | Orthogneiss | 1159.8 | 26.30 | 2s | 464.37 | Ducea et al. (2004) |
| M01-14 | Orthogneiss | 1004.5 | 22.20 | 2s | 464.37 | Ducea et al. (2004) |
| M01-14 | Orthogneiss | 1060.3 | 24.00 | 2s | 464.37 | Ducea et al. (2004) |
| M01-14 | Orthogneiss | 1043.4 | 23.30 | 2s | 464.37 | Ducea et al. (2004) |
| M01-14 | Orthogneiss | 1103.1 | 25.50 | 2s | 464.37 | Ducea et al. (2004) |
| M01-14 | Orthogneiss | 1063.8 | 24.10 | 2s | 464.37 | Ducea et al. (2004) |
| M01-14 | Orthogneiss | 1053.9 | 24.80 | 2s | 464.37 | Ducea et al. (2004) |
| M01-14 | Orthogneiss | 1068.7 | 25.30 | 2s | 464.37 | Ducea et al. (2004) |
| M01-14 | Orthogneiss | 1197.7 | 26.90 | 2s | 464.37 | Ducea et al. (2004) |
| M01-14 | Orthogneiss | 1007.8 | 23.20 | 2s | 464.37 | Ducea et al. (2004) |
| M01-14 | Orthogneiss | 1115.7 | 25.50 | 2s | 464.37 | Ducea et al. (2004) |
| M01-14 | Orthogneiss | 1007.8 | 23.20 | 2s | 464.37 | Ducea et al. (2004) |
| M01-14 | Orthogneiss | 1381.2 | 35.20 | 2s | 464.37 | Ducea et al. (2004) |
| M01-14 | Orthogneiss | 1174.8 | 26.80 | 2s | 464.37 | Ducea et al. (2004) |
| M01-14 | Orthogneiss | 1103.3 | 25.80 | 2s | 464.37 | Ducea et al. (2004) |
| M01-14 | Orthogneiss | 1197.0 | 27.70 | 2s | 464.37 | Ducea et al. (2004) |
| M01-14 | Orthogneiss | 1253.1 | 28.80 | 2s | 464.37 | Ducea et al. (2004) |
| M01-14 | Orthogneiss | 1070.9 | 24.40 | 2s | 464.37 | Ducea et al. (2004) |
| M01-14 | Orthogneiss | 1020.9 | 25.30 | 2s | 464.37 | Ducea et al. (2004) |
| M01-14 | Orthogneiss | 1279.8 | 32.10 | 2s | 464.37 | Ducea et al. (2004) |
| M01-14 | Orthogneiss | 1152.4 | 25.70 | 2s | 464.37 | Ducea et al. (2004) |
| M01-16 | Orthogneiss | 1084.6 | 29.70 | 2s | 469.47 | Ducea et al. (2004) |
| M01-16 | Orthogneiss | 1206.1 | 28.60 | 2s | 469.47 | Ducea et al. (2004) |
| M01-16 | Orthogneiss | 1223.2 | 60.60 | 2s | 469.47 | Ducea et al. (2004) |
| M01-16 | Orthogneiss | 1124.5 | 25.50 | 2s | 469.47 | Ducea et al. (2004) |
| M01-16 | Orthogneiss | 1015.3 | 23.80 | 2s | 469.47 | Ducea et al. (2004) |
| M01-16 | Orthogneiss | 1179.6 | 28.20 | 2s | 469.47 | Ducea et al. (2004) |
| M01-16 | Orthogneiss | 1188.3 | 27.70 | 2s | 469.47 | Ducea et al. (2004) |
| M01-16 | Orthogneiss | 1240.2 | 28.20 | 2s | 469.47 | Ducea et al. (2004) |
| M01-16 | Orthogneiss | 1041.6 | 36.80 | 2s | 469.47 | Ducea et al. (2004) |
| M01-16 | Orthogneiss | 1134.0 | 27.20 | 2s | 469.47 | Ducea et al. (2004) |
| M01-16 | Orthogneiss | 1127.2 | 27.30 | 2s | 469.47 | Ducea et al. (2004) |
| M01-16 | Orthogneiss | 1100.2 | 25.20 | 2s | 469.47 | Ducea et al. (2004) |
| M01-16 | Orthogneiss | 1090.5 | 24.20 | 2s | 469.47 | Ducea et al. (2004) |
| M01-16 | Orthogneiss | 1080.7 | 25.90 | 2s | 469.47 | Ducea et al. (2004) |
| M01-16 | Orthogneiss | 1108.1 | 26.20 | 2s | 469.47 | Ducea et al. (2004) |
| M01-16 | Orthogneiss | 1063.1 | 24.00 | 2s | 469.47 | Ducea et al. (2004) |
| M01-16 | Orthogneiss | 1119.5 | 27.80 | 2s | 469.47 | Ducea et al. (2004) |
| M01-16 | Orthogneiss | 1106.7 | 27.20 | 2s | 469.47 | Ducea et al. (2004) |
| M01-16 | Orthogneiss | 1085.3 | 25.10 | 2s | 469.47 | Ducea et al. (2004) |
| M01-16 | Orthogneiss | 1220.8 | 29.10 | 2s | 469.47 | Ducea et al. (2004) |
| M01-16 | Orthogneiss | 1058.3 | 27.50 | 2s | 469.47 | Ducea et al. (2004) |
| M01-16 | Orthogneiss | 1104.1 | 30.80 | 2s | 469.47 | Ducea et al. (2004) |
| M01-16 | Orthogneiss | 1162.2 | 26.00 | 2s | 469.47 | Ducea et al. (2004) |
| M01-16 | Orthogneiss | 1120.1 | 25.10 | 2s | 469.47 | Ducea et al. (2004) |
| M01-17 | Orthogneiss | 270.1 | 5.70 | 2s | 382.98 | Ducea et al. (2004) |
| M01-17 | Orthogneiss | 274.3 | 5.70 | 2s | 382.98 | Ducea et al. (2004) |
| M01-17 | Orthogneiss | 264.3 | 5.70 | 2s | 382.98 | Ducea et al. (2004) |
| M01-17 | Orthogneiss | 640.0 | 16.30 | 2s | 382.98 | Ducea et al. (2004) |
| M01-17 | Orthogneiss | 256.7 | 5.80 | 2s | 382.98 | Ducea et al. (2004) |
| M01-17 | Orthogneiss | 259.3 | 6.50 | 2s | 382.98 | Ducea et al. (2004) |
| M01-17 | Orthogneiss | 347.5 | 7.80 | 2s | 382.98 | Ducea et al. (2004) |
| M01-17 | Orthogneiss | 734.2 | 18.30 | 2s | 382.98 | Ducea et al. (2004) |
| M01-17 | Orthogneiss | 277.1 | 6.60 | 2s | 382.98 | Ducea et al. (2004) |
| M01-17 | Orthogneiss | 284.3 | 6.70 | 2s | 382.98 | Ducea et al. (2004) |
| M01-17 | Orthogneiss | 860.9 | 19.00 | 2s | 382.98 | Ducea et al. (2004) |
| M01-17 | Orthogneiss | 277.9 | 5.90 | 2s | 382.98 | Ducea et al. (2004) |
| M01-17 | Orthogneiss | 273.0 | 5.80 | 2s | 382.98 | Ducea et al. (2004) |
| M01-17 | Orthogneiss | 316.9 | 9.20 | 2s | 382.98 | Ducea et al. (2004) |
| M01-17 | Orthogneiss | 286.1 | 6.20 | 2s | 382.98 | Ducea et al. (2004) |
| M01-17 | Orthogneiss | 272.7 | 6.90 | 2s | 382.98 | Ducea et al. (2004) |
| M01-17 | Orthogneiss | 216.6 | 4.90 | 2s | 382.98 | Ducea et al. (2004) |
| M01-17 | Orthogneiss | 242.8 | 5.70 | 2s | 382.98 | Ducea et al. (2004) |
| M01-17 | Orthogneiss | 255.3 | 5.80 | 2s | 382.98 | Ducea et al. (2004) |
| M01-17 | Orthogneiss | 278.4 | 5.90 | 2s | 382.98 | Ducea et al. (2004) |
| M01-17 | Orthogneiss | 292.6 | 6.40 | 2s | 382.98 | Ducea et al. (2004) |
| M01-17 | Orthogneiss | 286.2 | 6.20 | 2s | 382.98 | Ducea et al. (2004) |
| M01-17 | Orthogneiss | 247.0 | 6.10 | 2s | 382.98 | Ducea et al. (2004) |
| M01-17 | Orthogneiss | 298.7 | 6.40 | 2s | 382.98 | Ducea et al. (2004) |
| M01-17 | Orthogneiss | 283.4 | 6.30 | 2s | 382.98 | Ducea et al. (2004) |
| M01-17 | Orthogneiss | 267.8 | 6.10 | 2s | 382.98 | Ducea et al. (2004) |
| M01-17 | Orthogneiss | 269.9 | 5.60 | 2s | 382.98 | Ducea et al. (2004) |
| M01-19 | Granodiorite | 665.2 | 21.60 | 2s | 388.33 | Ducea et al. (2004) |
| M01-19 | Granodiorite | 164.9 | 3.60 | 2s | 388.33 | Ducea et al. (2004) |
| M01-19 | Granodiorite | 154.6 | 3.30 | 2s | 388.33 | Ducea et al. (2004) |
| M01-19 | Granodiorite | 144.1 | 3.40 | 2s | 388.33 | Ducea et al. (2004) |
| M01-19 | Granodiorite | 155.4 | 3.30 | 2s | 388.33 | Ducea et al. (2004) |
| M01-19 | Granodiorite | 165.0 | 3.50 | 2s | 388.33 | Ducea et al. (2004) |
| M01-19 | Granodiorite | 947.9 | 21.20 | 2s | 388.33 | Ducea et al. (2004) |
| M01-19 | Granodiorite | 312.3 | 6.70 | 2s | 388.33 | Ducea et al. (2004) |
| M01-19 | Granodiorite | 170.8 | 3.80 | 2s | 388.33 | Ducea et al. (2004) |
| M01-19 | Granodiorite | 747.3 | 18.00 | 2s | 388.33 | Ducea et al. (2004) |
| M01-19 | Granodiorite | 178.0 | 4.40 | 2s | 388.33 | Ducea et al. (2004) |
| M01-19 | Granodiorite | 222.6 | 5.50 | 2s | 388.33 | Ducea et al. (2004) |
| M01-19 | Granodiorite | 166.6 | 3.50 | 2s | 388.33 | Ducea et al. (2004) |
| M01-19 | Granodiorite | 943.1 | 22.50 | 2s | 388.33 | Ducea et al. (2004) |
| M01-19 | Granodiorite | 147.1 | 3.40 | 2s | 388.33 | Ducea et al. (2004) |
| M01-19 | Granodiorite | 154.1 | 3.30 | 2s | 388.33 | Ducea et al. (2004) |
| M01-19 | Granodiorite | 154.3 | 3.60 | 2s | 388.33 | Ducea et al. (2004) |
| M01-19 | Granodiorite | 139.0 | 3.10 | 2s | 388.33 | Ducea et al. (2004) |
| M01-19 | Granodiorite | 1008.2 | 23.30 | 2s | 388.33 | Ducea et al. (2004) |
| M01-19 | Granodiorite | 191.4 | 4.10 | 2s | 388.33 | Ducea et al. (2004) |
| M01-19 | Granodiorite | 1027.5 | 26.80 | 2s | 388.33 | Ducea et al. (2004) |
| M01-19 | Granodiorite | 195.0 | 7.00 | 2s | 388.33 | Ducea et al. (2004) |
| M01-19 | Granodiorite | 172.1 | 4.00 | 2s | 388.33 | Ducea et al. (2004) |
| M01-19 | Granodiorite | 293.0 | 6.20 | 2s | 388.33 | Ducea et al. (2004) |
| M01-19 | Granodiorite | 264.1 | 7.70 | 2s | 388.33 | Ducea et al. (2004) |
| M01-19 | Granodiorite | 554.6 | 12.90 | 2s | 388.33 | Ducea et al. (2004) |
| M01-19 | Granodiorite | 181.5 | 5.40 | 2s | 388.33 | Ducea et al. (2004) |
| M01-26 | Diorite | 65.9 | 1.60 | 2s | 394.79 | Ducea et al. (2004) |
| M01-26 | Diorite | 846.5 | 20.00 | 2s | 394.79 | Ducea et al. (2004) |
| M01-26 | Diorite | 21.8 | 2.80 | 2s | 394.79 | Ducea et al. (2004) |
| M01-26 | Diorite | 124.1 | 3.30 | 2s | 394.79 | Ducea et al. (2004) |
| M01-26 | Diorite | 44.1 | 4.20 | 2s | 394.79 | Ducea et al. (2004) |
| M01-26 | Diorite | 24.2 | 0.50 | 2s | 394.79 | Ducea et al. (2004) |
| M01-26 | Diorite | 380.6 | 17.10 | 2s | 394.79 | Ducea et al. (2004) |
| M01-26 | Diorite | 40.8 | 1.50 | 2s | 394.79 | Ducea et al. (2004) |
| M01-26 | Diorite | 30.3 | 1.20 | 2s | 394.79 | Ducea et al. (2004) |
| M01-26 | Diorite | 622.5 | 16.10 | 2s | 394.79 | Ducea et al. (2004) |
| M01-26 | Diorite | 905.9 | 22.40 | 2s | 394.79 | Ducea et al. (2004) |
| M01-26 | Diorite | 29.8 | 0.80 | 2s | 394.79 | Ducea et al. (2004) |
| M01-26 | Diorite | 25.3 | 1.00 | 2s | 394.79 | Ducea et al. (2004) |
| M01-26 | Diorite | 521.9 | 12.80 | 2s | 394.79 | Ducea et al. (2004) |
| M01-26 | Diorite | 938.9 | 21.00 | 2s | 394.79 | Ducea et al. (2004) |
| M01-26 | Diorite | 42.4 | 1.30 | 2s | 394.79 | Ducea et al. (2004) |
| M01-26 | Diorite | 773.6 | 17.20 | 2s | 394.79 | Ducea et al. (2004) |
| M01-26 | Diorite | 22.3 | 0.50 | 2s | 394.79 | Ducea et al. (2004) |
| M01-26 | Diorite | 72.1 | 2.10 | 2s | 394.79 | Ducea et al. (2004) |
| M01-26 | Diorite | 31.8 | 1.00 | 2s | 394.79 | Ducea et al. (2004) |
| M01-26 | Diorite | 24.2 | 0.80 | 2s | 394.79 | Ducea et al. (2004) |
| M01-27 | Leucosome? | 33.5 | 0.70 | 2s | 398.27 | Ducea et al. (2004) |
| M01-27 | Leucosome? | 34.6 | 0.90 | 2s | 398.27 | Ducea et al. (2004) |
| M01-27 | Leucosome? | 33.4 | 0.80 | 2s | 398.27 | Ducea et al. (2004) |
| M01-27 | Leucosome? | 36.4 | 1.50 | 2s | 398.27 | Ducea et al. (2004) |
| M01-27 | Leucosome? | 31.1 | 0.70 | 2s | 398.27 | Ducea et al. (2004) |
| M01-27 | Leucosome? | 33.6 | 1.20 | 2s | 398.27 | Ducea et al. (2004) |
| M01-27 | Leucosome? | 33.0 | 0.80 | 2s | 398.27 | Ducea et al. (2004) |
| M01-27 | Leucosome? | 33.1 | 0.90 | 2s | 398.27 | Ducea et al. (2004) |
| M01-27 | Leucosome? | 28.4 | 0.80 | 2s | 398.27 | Ducea et al. (2004) |
| M01-27 | Leucosome? | 27.5 | 0.80 | 2s | 398.27 | Ducea et al. (2004) |
| M01-27 | Leucosome? | 34.6 | 2.00 | 2s | 398.27 | Ducea et al. (2004) |
| M01-27 | Leucosome? | 31.0 | 0.90 | 2s | 398.27 | Ducea et al. (2004) |
| M01-27 | Leucosome? | 33.2 | 1.30 | 2s | 398.27 | Ducea et al. (2004) |
| M01-27 | Leucosome? | 27.0 | 0.60 | 2s | 398.27 | Ducea et al. (2004) |
| M01-27 | Leucosome? | 29.3 | 1.10 | 2s | 398.27 | Ducea et al. (2004) |
| M01-27 | Leucosome? | 31.9 | 1.50 | 2s | 398.27 | Ducea et al. (2004) |
| M01-27 | Leucosome? | 32.4 | 2.30 | 2s | 398.27 | Ducea et al. (2004) |
| M01-27 | Leucosome? | 31.8 | 0.80 | 2s | 398.27 | Ducea et al. (2004) |
| M01-27 | Leucosome? | 33.0 | 2.10 | 2s | 398.27 | Ducea et al. (2004) |
| M01-27 | Leucosome? | 30.0 | 1.10 | 2s | 398.27 | Ducea et al. (2004) |
| M01-27 | Leucosome? | 174.8 | 3.80 | 2s | 398.27 | Ducea et al. (2004) |
| M01-27 | Leucosome? | 31.5 | 1.10 | 2s | 398.27 | Ducea et al. (2004) |
| M01-27 | Leucosome? | 42.2 | 1.30 | 2s | 398.27 | Ducea et al. (2004) |
| M01-27 | Leucosome? | 32.9 | 1.40 | 2s | 398.27 | Ducea et al. (2004) |
| M01-27 | Leucosome? | 1016.3 | 28.00 | 2s | 398.27 | Ducea et al. (2004) |
| M01-28 | Leucosome? | 23.7 | 0.50 | 2s | 381.77 | Ducea et al. (2004) |
| M01-28 | Leucosome? | 96.7 | 2.60 | 2s | 381.77 | Ducea et al. (2004) |
| M01-28 | Leucosome? | 118.4 | 3.40 | 2s | 381.77 | Ducea et al. (2004) |
| M01-28 | Leucosome? | 123.2 | 2.60 | 2s | 381.77 | Ducea et al. (2004) |
| M01-28 | Leucosome? | 125.9 | 3.50 | 2s | 381.77 | Ducea et al. (2004) |
| M01-28 | Leucosome? | 36.3 | 0.90 | 2s | 381.77 | Ducea et al. (2004) |
| M01-28 | Leucosome? | 31.8 | 0.70 | 2s | 381.77 | Ducea et al. (2004) |
| M01-28 | Leucosome? | 37.8 | 0.90 | 2s | 381.77 | Ducea et al. (2004) |
| M01-28 | Leucosome? | 30.6 | 6.40 | 2s | 381.77 | Ducea et al. (2004) |
| M01-28 | Leucosome? | 28.9 | 0.70 | 2s | 381.77 | Ducea et al. (2004) |
| M01-28 | Leucosome? | 121.4 | 3.10 | 2s | 381.77 | Ducea et al. (2004) |
| M01-28 | Leucosome? | 31.8 | 1.20 | 2s | 381.77 | Ducea et al. (2004) |
| M01-28 | Leucosome? | 27.6 | 0.70 | 2s | 381.77 | Ducea et al. (2004) |
| M01-28 | Leucosome? | 66.9 | 2.00 | 2s | 381.77 | Ducea et al. (2004) |
| M01-28 | Leucosome? | 122.8 | 3.00 | 2s | 381.77 | Ducea et al. (2004) |
| M01-28 | Leucosome? | 57.5 | 1.20 | 2s | 381.77 | Ducea et al. (2004) |
| M01-28 | Leucosome? | 62.5 | 1.40 | 2s | 381.77 | Ducea et al. (2004) |
| M01-28 | Leucosome? | 1051.0 | 26.50 | 2s | 381.77 | Ducea et al. (2004) |
| M01-28 | Leucosome? | 55.5 | 1.30 | 2s | 381.77 | Ducea et al. (2004) |
| M01-28 | Leucosome? | 49.0 | 1.30 | 2s | 381.77 | Ducea et al. (2004) |
| M01-28 | Leucosome? | 100.9 | 3.00 | 2s | 381.77 | Ducea et al. (2004) |
| M01-28 | Leucosome? | 38.1 | 1.70 | 2s | 381.77 | Ducea et al. (2004) |
| Rb109 | Orthogneiss | 54.1 | 1.00 | 2s | 101.48 | Estrada-Carmona et al. (2016) |
| Rb109 | Orthogneiss | 54.4 | 1.00 | 2s | 101.48 | Estrada-Carmona et al. (2016) |
| Rb109 | Orthogneiss | 52.3 | 1.00 | 2s | 101.48 | Estrada-Carmona et al. (2016) |
| Rb109 | Orthogneiss | 89.0 | 3.00 | 2s | 101.48 | Estrada-Carmona et al. (2016) |
| Rb109 | Orthogneiss | 54.5 | 2.00 | 2s | 101.48 | Estrada-Carmona et al. (2016) |
| Rb109 | Orthogneiss | 133.0 | 3.00 | 2s | 101.48 | Estrada-Carmona et al. (2016) |
| Rb109 | Orthogneiss | 122.8 | 3.00 | 2s | 101.48 | Estrada-Carmona et al. (2016) |
| Rb109 | Orthogneiss | 124.0 | 3.00 | 2s | 101.48 | Estrada-Carmona et al. (2016) |
| Rb109 | Orthogneiss | 54.9 | 1.00 | 2s | 101.48 | Estrada-Carmona et al. (2016) |
| Rb109 | Orthogneiss | 61.9 | 2.00 | 2s | 101.48 | Estrada-Carmona et al. (2016) |
| Rb109 | Orthogneiss | 52.3 | 1.00 | 2s | 101.48 | Estrada-Carmona et al. (2016) |
| Rb109 | Orthogneiss | 53.8 | 1.00 | 2s | 101.48 | Estrada-Carmona et al. (2016) |
| Rb109 | Orthogneiss | 55.2 | 1.00 | 2s | 101.48 | Estrada-Carmona et al. (2016) |
| Rb109 | Orthogneiss | 57.3 | 2.00 | 2s | 101.48 | Estrada-Carmona et al. (2016) |
| Rb109 | Orthogneiss | 66.4 | 2.00 | 2s | 101.48 | Estrada-Carmona et al. (2016) |
| Rb109 | Orthogneiss | 126.4 | 3.00 | 2s | 101.48 | Estrada-Carmona et al. (2016) |
| Rb109 | Orthogneiss | 53.7 | 1.00 | 2s | 101.48 | Estrada-Carmona et al. (2016) |
| Rb109 | Orthogneiss | 118.3 | 3.00 | 2s | 101.48 | Estrada-Carmona et al. (2016) |
| Rb109 | Orthogneiss | 55.5 | 1.00 | 2s | 101.48 | Estrada-Carmona et al. (2016) |
| Rb109 | Orthogneiss | 54.7 | 1.00 | 2s | 101.48 | Estrada-Carmona et al. (2016) |
| Rb109 | Orthogneiss | 53.6 | 1.00 | 2s | 101.48 | Estrada-Carmona et al. (2016) |
| Rb109 | Orthogneiss | 54.4 | 1.00 | 2s | 101.48 | Estrada-Carmona et al. (2016) |
| Rb109 | Orthogneiss | 61.8 | 2.00 | 2s | 101.48 | Estrada-Carmona et al. (2016) |
| Rb109 | Orthogneiss | 54.5 | 1.00 | 2s | 101.48 | Estrada-Carmona et al. (2016) |
| Rb109 | Orthogneiss | 54.4 | 1.00 | 2s | 101.48 | Estrada-Carmona et al. (2016) |
| Rb109 | Orthogneiss | 53.9 | 1.00 | 2s | 101.48 | Estrada-Carmona et al. (2016) |
| Rb109 | Orthogneiss | 56.3 | 2.00 | 2s | 101.48 | Estrada-Carmona et al. (2016) |
| Rb109 | Orthogneiss | 101.8 | 3.00 | 2s | 101.48 | Estrada-Carmona et al. (2016) |
| Rb109 | Orthogneiss | 120.3 | 3.00 | 2s | 101.48 | Estrada-Carmona et al. (2016) |
| Rb109 | Orthogneiss | 123.5 | 3.00 | 2s | 101.48 | Estrada-Carmona et al. (2016) |
| Rb109 | Orthogneiss | 57.0 | 1.00 | 2s | 101.48 | Estrada-Carmona et al. (2016) |
| Rb109 | Orthogneiss | 132.5 | 3.00 | 2s | 101.48 | Estrada-Carmona et al. (2016) |
| Rb109 | Orthogneiss | 54.3 | 1.00 | 2s | 101.48 | Estrada-Carmona et al. (2016) |
| Rb109 | Orthogneiss | 53.9 | 1.00 | 2s | 101.48 | Estrada-Carmona et al. (2016) |
| Rb109 | Orthogneiss | 50.5 | 2.00 | 2s | 101.48 | Estrada-Carmona et al. (2016) |
| Rb109 | Orthogneiss | 61.5 | 2.00 | 2s | 101.48 | Estrada-Carmona et al. (2016) |
| Rb109 | Orthogneiss | 121.0 | 3.00 | 2s | 101.48 | Estrada-Carmona et al. (2016) |
| Rb109 | Orthogneiss | 121.2 | 4.00 | 2s | 101.48 | Estrada-Carmona et al. (2016) |
| Rb109 | Orthogneiss | 123.8 | 4.00 | 2s | 101.48 | Estrada-Carmona et al. (2016) |
| Rb109 | Orthogneiss | 128.6 | 5.00 | 2s | 101.48 | Estrada-Carmona et al. (2016) |
| Rb109 | Orthogneiss | 54.0 | 1.00 | 2s | 101.48 | Estrada-Carmona et al. (2016) |
| Rb109 | Orthogneiss | 55.1 | 1.00 | 2s | 101.48 | Estrada-Carmona et al. (2016) |
| Rb109 | Orthogneiss | 57.9 | 8.00 | 2s | 101.48 | Estrada-Carmona et al. (2016) |
| Rb109 | Orthogneiss | 54.6 | 1.00 | 2s | 101.48 | Estrada-Carmona et al. (2016) |
| Rb109 | Orthogneiss | 54.8 | 1.00 | 2s | 101.48 | Estrada-Carmona et al. (2016) |
| Rb109 | Orthogneiss | 96.4 | 2.00 | 2s | 101.48 | Estrada-Carmona et al. (2016) |
| Rb109 | Orthogneiss | 54.4 | 1.00 | 2s | 101.48 | Estrada-Carmona et al. (2016) |
| Rb109 | Orthogneiss | 125.4 | 3.00 | 2s | 101.48 | Estrada-Carmona et al. (2016) |
| Rb109 | Orthogneiss | 54.3 | 1.00 | 2s | 101.48 | Estrada-Carmona et al. (2016) |
| Xo0222 | Orthogneiss | 120.8 | 5.00 | 2s | 99.42 | Estrada-Carmona et al. (2016) |
| Xo0222 | Orthogneiss | 2720.0 | 110.00 | 2s | 99.42 | Estrada-Carmona et al. (2016) |
| Xo0222 | Orthogneiss | 125.1 | 4.00 | 2s | 99.42 | Estrada-Carmona et al. (2016) |
| Xo0222 | Orthogneiss | 109.9 | 3.00 | 2s | 99.42 | Estrada-Carmona et al. (2016) |
| Xo0222 | Orthogneiss | 121.9 | 3.00 | 2s | 99.42 | Estrada-Carmona et al. (2016) |
| Xo0222 | Orthogneiss | 118.9 | 4.00 | 2s | 99.42 | Estrada-Carmona et al. (2016) |
| Xo0222 | Orthogneiss | 125.3 | 4.00 | 2s | 99.42 | Estrada-Carmona et al. (2016) |
| Xo0222 | Orthogneiss | 127.4 | 3.00 | 2s | 99.42 | Estrada-Carmona et al. (2016) |
| Xo0222 | Orthogneiss | 121.2 | 3.00 | 2s | 99.42 | Estrada-Carmona et al. (2016) |
| Xo0222 | Orthogneiss | 123.6 | 4.00 | 2s | 99.42 | Estrada-Carmona et al. (2016) |
| Xo0222 | Orthogneiss | 119.9 | 3.00 | 2s | 99.42 | Estrada-Carmona et al. (2016) |
| Xo0222 | Orthogneiss | 126.0 | 4.00 | 2s | 99.42 | Estrada-Carmona et al. (2016) |
| Xo0222 | Orthogneiss | 100.7 | 5.00 | 2s | 99.42 | Estrada-Carmona et al. (2016) |
| Xo0222 | Orthogneiss | 87.6 | 2.00 | 2s | 99.42 | Estrada-Carmona et al. (2016) |
| Xo0222 | Orthogneiss | 125.7 | 3.00 | 2s | 99.42 | Estrada-Carmona et al. (2016) |
| Xo0222 | Orthogneiss | 123.4 | 4.00 | 2s | 99.42 | Estrada-Carmona et al. (2016) |
| Xo0222 | Orthogneiss | 118.3 | 6.00 | 2s | 99.42 | Estrada-Carmona et al. (2016) |
| Xo0222 | Orthogneiss | 97.2 | 3.00 | 2s | 99.42 | Estrada-Carmona et al. (2016) |
| Xo0222 | Orthogneiss | 119.1 | 3.00 | 2s | 99.42 | Estrada-Carmona et al. (2016) |
| Xo0222 | Orthogneiss | 299.0 | 10.00 | 2s | 99.42 | Estrada-Carmona et al. (2016) |
| Xo0222 | Orthogneiss | 124.0 | 3.00 | 2s | 99.42 | Estrada-Carmona et al. (2016) |
| Xo0222 | Orthogneiss | 118.7 | 3.00 | 2s | 99.42 | Estrada-Carmona et al. (2016) |
| Xo0222 | Orthogneiss | 116.2 | 4.00 | 2s | 99.42 | Estrada-Carmona et al. (2016) |
| Xo0222 | Orthogneiss | 134.3 | 5.00 | 2s | 99.42 | Estrada-Carmona et al. (2016) |
| Xo0222 | Orthogneiss | 119.6 | 3.00 | 2s | 99.42 | Estrada-Carmona et al. (2016) |
| Xo0222 | Orthogneiss | 129.3 | 4.00 | 2s | 99.42 | Estrada-Carmona et al. (2016) |
| Xo0222 | Orthogneiss | 123.3 | 4.00 | 2s | 99.42 | Estrada-Carmona et al. (2016) |
| Xo0222 | Orthogneiss | 115.5 | 4.00 | 2s | 99.42 | Estrada-Carmona et al. (2016) |
| Xo0222 | Orthogneiss | 125.2 | 3.00 | 2s | 99.42 | Estrada-Carmona et al. (2016) |
| Xo0222 | Orthogneiss | 121.2 | 3.00 | 2s | 99.42 | Estrada-Carmona et al. (2016) |
| Xo0222 | Orthogneiss | 117.3 | 3.00 | 2s | 99.42 | Estrada-Carmona et al. (2016) |
| Xo0222 | Orthogneiss | 3310.0 | 330.00 | 2s | 99.42 | Estrada-Carmona et al. (2016) |
| Xo0222 | Orthogneiss | 121.4 | 4.00 | 2s | 99.42 | Estrada-Carmona et al. (2016) |
| Xo0222 | Orthogneiss | 118.5 | 4.00 | 2s | 99.42 | Estrada-Carmona et al. (2016) |
| Xo0222 | Orthogneiss | 123.4 | 4.00 | 2s | 99.42 | Estrada-Carmona et al. (2016) |
| Xo0222 | Orthogneiss | 119.2 | 3.00 | 2s | 99.42 | Estrada-Carmona et al. (2016) |
| Xo0222 | Orthogneiss | 116.9 | 3.00 | 2s | 99.42 | Estrada-Carmona et al. (2016) |
| Xo0222 | Orthogneiss | 121.4 | 3.00 | 2s | 99.42 | Estrada-Carmona et al. (2016) |
| Xo0222 | Orthogneiss | 119.1 | 4.00 | 2s | 99.42 | Estrada-Carmona et al. (2016) |
| Xo0222 | Orthogneiss | 121.0 | 3.00 | 2s | 99.42 | Estrada-Carmona et al. (2016) |
| Xo0222 | Orthogneiss | 122.8 | 3.00 | 2s | 99.42 | Estrada-Carmona et al. (2016) |
| Xo0222 | Orthogneiss | 118.4 | 3.00 | 2s | 99.42 | Estrada-Carmona et al. (2016) |
| Xo0222 | Orthogneiss | 114.5 | 4.00 | 2s | 99.42 | Estrada-Carmona et al. (2016) |
| Xo0222 | Orthogneiss | 122.8 | 3.00 | 2s | 99.42 | Estrada-Carmona et al. (2016) |
| Xo0222 | Orthogneiss | 123.2 | 3.00 | 2s | 99.42 | Estrada-Carmona et al. (2016) |
| Xo0222 | Orthogneiss | 123.8 | 3.00 | 2s | 99.42 | Estrada-Carmona et al. (2016) |
| Xo0222 | Orthogneiss | 125.9 | 4.00 | 2s | 99.42 | Estrada-Carmona et al. (2016) |
| Xo0222 | Orthogneiss | 118.8 | 4.00 | 2s | 99.42 | Estrada-Carmona et al. (2016) |
| Xo0230 | Orthogneiss | 125.9 | 5.00 | 2s | 76.22 | Estrada-Carmona et al. (2016) |
| Xo0230 | Orthogneiss | 127.2 | 4.00 | 2s | 76.22 | Estrada-Carmona et al. (2016) |
| Xo0230 | Orthogneiss | 121.4 | 3.00 | 2s | 76.22 | Estrada-Carmona et al. (2016) |
| Xo0230 | Orthogneiss | 113.3 | 3.00 | 2s | 76.22 | Estrada-Carmona et al. (2016) |
| Xo0230 | Orthogneiss | 127.4 | 3.00 | 2s | 76.22 | Estrada-Carmona et al. (2016) |
| Xo0230 | Orthogneiss | 123.6 | 3.00 | 2s | 76.22 | Estrada-Carmona et al. (2016) |
| Xo0230 | Orthogneiss | 125.3 | 4.00 | 2s | 76.22 | Estrada-Carmona et al. (2016) |
| Xo0230 | Orthogneiss | 118.0 | 3.00 | 2s | 76.22 | Estrada-Carmona et al. (2016) |
| Xo0230 | Orthogneiss | 121.8 | 3.00 | 2s | 76.22 | Estrada-Carmona et al. (2016) |
| Xo0230 | Orthogneiss | 121.9 | 4.00 | 2s | 76.22 | Estrada-Carmona et al. (2016) |
| Xo0230 | Orthogneiss | 123.7 | 3.00 | 2s | 76.22 | Estrada-Carmona et al. (2016) |
| Xo0230 | Orthogneiss | 122.8 | 4.00 | 2s | 76.22 | Estrada-Carmona et al. (2016) |
| Xo0230 | Orthogneiss | 127.0 | 3.00 | 2s | 76.22 | Estrada-Carmona et al. (2016) |
| Xo0230 | Orthogneiss | 116.2 | 3.00 | 2s | 76.22 | Estrada-Carmona et al. (2016) |
| Xo0230 | Orthogneiss | 123.3 | 3.00 | 2s | 76.22 | Estrada-Carmona et al. (2016) |
| Xo0230 | Orthogneiss | 127.1 | 3.00 | 2s | 76.22 | Estrada-Carmona et al. (2016) |
| Xo0230 | Orthogneiss | 119.4 | 3.00 | 2s | 76.22 | Estrada-Carmona et al. (2016) |
| Xo0230 | Orthogneiss | 123.6 | 3.00 | 2s | 76.22 | Estrada-Carmona et al. (2016) |
| Xo0230 | Orthogneiss | 121.7 | 4.00 | 2s | 76.22 | Estrada-Carmona et al. (2016) |
| Xo0230 | Orthogneiss | 122.3 | 4.00 | 2s | 76.22 | Estrada-Carmona et al. (2016) |
| Xo0230 | Orthogneiss | 123.8 | 3.00 | 2s | 76.22 | Estrada-Carmona et al. (2016) |
| Xo0230 | Orthogneiss | 122.1 | 3.00 | 2s | 76.22 | Estrada-Carmona et al. (2016) |
| Xo0230 | Orthogneiss | 139.4 | 4.00 | 2s | 76.22 | Estrada-Carmona et al. (2016) |
| Xo0230 | Orthogneiss | 123.7 | 4.00 | 2s | 76.22 | Estrada-Carmona et al. (2016) |
| Xo0230 | Orthogneiss | 146.0 | 10.00 | 2s | 76.22 | Estrada-Carmona et al. (2016) |
| Xo0230 | Orthogneiss | 122.3 | 3.00 | 2s | 76.22 | Estrada-Carmona et al. (2016) |
| Xo0230 | Orthogneiss | 124.0 | 4.00 | 2s | 76.22 | Estrada-Carmona et al. (2016) |
| Xo0230 | Orthogneiss | 128.0 | 3.00 | 2s | 76.22 | Estrada-Carmona et al. (2016) |
| Xo0230 | Orthogneiss | 122.3 | 3.00 | 2s | 76.22 | Estrada-Carmona et al. (2016) |
| Xo0230 | Orthogneiss | 109.2 | 4.00 | 2s | 76.22 | Estrada-Carmona et al. (2016) |
| Xo0230 | Orthogneiss | 118.2 | 3.00 | 2s | 76.22 | Estrada-Carmona et al. (2016) |
| Xo0230 | Orthogneiss | 126.0 | 3.00 | 2s | 76.22 | Estrada-Carmona et al. (2016) |
| Xo0230 | Orthogneiss | 123.0 | 3.00 | 2s | 76.22 | Estrada-Carmona et al. (2016) |
| Xo0230 | Orthogneiss | 124.4 | 3.00 | 2s | 76.22 | Estrada-Carmona et al. (2016) |
| Xo0230 | Orthogneiss | 109.5 | 3.00 | 2s | 76.22 | Estrada-Carmona et al. (2016) |
| Xo0230 | Orthogneiss | 124.9 | 3.00 | 2s | 76.22 | Estrada-Carmona et al. (2016) |
| Xo0230 | Orthogneiss | 111.5 | 4.00 | 2s | 76.22 | Estrada-Carmona et al. (2016) |
| Xo0230 | Orthogneiss | 131.3 | 4.00 | 2s | 76.22 | Estrada-Carmona et al. (2016) |
| Xo0230 | Orthogneiss | 121.8 | 3.00 | 2s | 76.22 | Estrada-Carmona et al. (2016) |
| Xo0230 | Orthogneiss | 120.7 | 3.00 | 2s | 76.22 | Estrada-Carmona et al. (2016) |
| Xo0230 | Orthogneiss | 124.7 | 3.00 | 2s | 76.22 | Estrada-Carmona et al. (2016) |
| Xo0230 | Orthogneiss | 122.1 | 3.00 | 2s | 76.22 | Estrada-Carmona et al. (2016) |
| Xo0230 | Orthogneiss | 138.9 | 4.00 | 2s | 76.22 | Estrada-Carmona et al. (2016) |
| Xo0230 | Orthogneiss | 66.6 | 3.00 | 2s | 76.22 | Estrada-Carmona et al. (2016) |
| Xo0230 | Orthogneiss | 125.3 | 5.00 | 2s | 76.22 | Estrada-Carmona et al. (2016) |
| Xo0230 | Orthogneiss | 119.9 | 3.00 | 2s | 76.22 | Estrada-Carmona et al. (2016) |
| Xo0230 | Orthogneiss | 126.0 | 3.00 | 2s | 76.22 | Estrada-Carmona et al. (2016) |
| Xo0230 | Orthogneiss | 109.9 | 4.00 | 2s | 76.22 | Estrada-Carmona et al. (2016) |
| Xo0230 | Orthogneiss | 124.5 | 3.00 | 2s | 76.22 | Estrada-Carmona et al. (2016) |
| Rb71 | Orthogneiss | 123.8 | 4.00 | 2s | 85.21 | Estrada-Carmona et al. (2016) |
| Rb71 | Orthogneiss | 128.2 | 4.00 | 2s | 85.21 | Estrada-Carmona et al. (2016) |
| Rb71 | Orthogneiss | 124.4 | 5.00 | 2s | 85.21 | Estrada-Carmona et al. (2016) |
| Rb71 | Orthogneiss | 126.6 | 4.00 | 2s | 85.21 | Estrada-Carmona et al. (2016) |
| Rb71 | Orthogneiss | 124.9 | 4.00 | 2s | 85.21 | Estrada-Carmona et al. (2016) |
| Rb71 | Orthogneiss | 122.3 | 4.00 | 2s | 85.21 | Estrada-Carmona et al. (2016) |
| Rb71 | Orthogneiss | 121.9 | 4.00 | 2s | 85.21 | Estrada-Carmona et al. (2016) |
| Rb71 | Orthogneiss | 128.3 | 4.00 | 2s | 85.21 | Estrada-Carmona et al. (2016) |
| Rb71 | Orthogneiss | 125.9 | 4.00 | 2s | 85.21 | Estrada-Carmona et al. (2016) |
| Rb71 | Orthogneiss | 124.9 | 5.00 | 2s | 85.21 | Estrada-Carmona et al. (2016) |
| Rb71 | Orthogneiss | 123.4 | 8.00 | 2s | 85.21 | Estrada-Carmona et al. (2016) |
| Rb71 | Orthogneiss | 133.6 | 4.00 | 2s | 85.21 | Estrada-Carmona et al. (2016) |
| Rb71 | Orthogneiss | 129.3 | 4.00 | 2s | 85.21 | Estrada-Carmona et al. (2016) |
| Rb71 | Orthogneiss | 124.6 | 6.00 | 2s | 85.21 | Estrada-Carmona et al. (2016) |
| Rb71 | Orthogneiss | 134.6 | 5.00 | 2s | 85.21 | Estrada-Carmona et al. (2016) |
| Rb71 | Orthogneiss | 135.6 | 5.00 | 2s | 85.21 | Estrada-Carmona et al. (2016) |
| Rb71 | Orthogneiss | 125.8 | 4.00 | 2s | 85.21 | Estrada-Carmona et al. (2016) |
| Rb71 | Orthogneiss | 129.8 | 7.00 | 2s | 85.21 | Estrada-Carmona et al. (2016) |
| Rb71 | Orthogneiss | 122.9 | 4.00 | 2s | 85.21 | Estrada-Carmona et al. (2016) |
| Rb71 | Orthogneiss | 132.3 | 4.00 | 2s | 85.21 | Estrada-Carmona et al. (2016) |
| Rb71 | Orthogneiss | 125.3 | 4.00 | 2s | 85.21 | Estrada-Carmona et al. (2016) |
| Rb71 | Orthogneiss | 127.5 | 5.00 | 2s | 85.21 | Estrada-Carmona et al. (2016) |
| Rb71 | Orthogneiss | 124.1 | 4.00 | 2s | 85.21 | Estrada-Carmona et al. (2016) |
| Rb71 | Orthogneiss | 105.5 | 8.00 | 2s | 85.21 | Estrada-Carmona et al. (2016) |
| Rb71 | Orthogneiss | 130.3 | 4.00 | 2s | 85.21 | Estrada-Carmona et al. (2016) |
| Rb71 | Orthogneiss | 130.1 | 4.00 | 2s | 85.21 | Estrada-Carmona et al. (2016) |
| Rb71 | Orthogneiss | 133.7 | 4.00 | 2s | 85.21 | Estrada-Carmona et al. (2016) |
| Rb71 | Orthogneiss | 148.2 | 5.00 | 2s | 85.21 | Estrada-Carmona et al. (2016) |
| Rb71 | Orthogneiss | 102.1 | 3.00 | 2s | 85.21 | Estrada-Carmona et al. (2016) |
| Rb71 | Orthogneiss | 134.5 | 5.00 | 2s | 85.21 | Estrada-Carmona et al. (2016) |
| Rb71 | Orthogneiss | 134.1 | 5.00 | 2s | 85.21 | Estrada-Carmona et al. (2016) |
| Rb71 | Orthogneiss | 129.1 | 4.00 | 2s | 85.21 | Estrada-Carmona et al. (2016) |
| Rb71 | Orthogneiss | 130.3 | 5.00 | 2s | 85.21 | Estrada-Carmona et al. (2016) |
| Rb71 | Orthogneiss | 130.1 | 4.00 | 2s | 85.21 | Estrada-Carmona et al. (2016) |
| Rb71 | Orthogneiss | 132.8 | 5.00 | 2s | 85.21 | Estrada-Carmona et al. (2016) |
| Rb71 | Orthogneiss | 129.9 | 4.00 | 2s | 85.21 | Estrada-Carmona et al. (2016) |
| Rb71 | Orthogneiss | 92.7 | 3.00 | 2s | 85.21 | Estrada-Carmona et al. (2016) |
| Rb71 | Orthogneiss | 125.7 | 4.00 | 2s | 85.21 | Estrada-Carmona et al. (2016) |
| Rb71 | Orthogneiss | 132.7 | 4.00 | 2s | 85.21 | Estrada-Carmona et al. (2016) |
| Rb71 | Orthogneiss | 136.0 | 5.00 | 2s | 85.21 | Estrada-Carmona et al. (2016) |
| Rb71 | Orthogneiss | 135.1 | 5.00 | 2s | 85.21 | Estrada-Carmona et al. (2016) |
| Rb76 | Orthogneiss | 128.6 | 4.00 | 2s | 100.85 | Estrada-Carmona et al. (2016) |
| Rb76 | Orthogneiss | 128.2 | 4.00 | 2s | 100.85 | Estrada-Carmona et al. (2016) |
| Rb76 | Orthogneiss | 134.1 | 5.00 | 2s | 100.85 | Estrada-Carmona et al. (2016) |
| Rb76 | Orthogneiss | 124.6 | 4.00 | 2s | 100.85 | Estrada-Carmona et al. (2016) |
| Rb76 | Orthogneiss | 133.7 | 4.00 | 2s | 100.85 | Estrada-Carmona et al. (2016) |
| Rb76 | Orthogneiss | 140.4 | 5.00 | 2s | 100.85 | Estrada-Carmona et al. (2016) |
| Rb76 | Orthogneiss | 119.9 | 4.00 | 2s | 100.85 | Estrada-Carmona et al. (2016) |
| Rb76 | Orthogneiss | 120.7 | 4.00 | 2s | 100.85 | Estrada-Carmona et al. (2016) |
| Rb76 | Orthogneiss | 132.0 | 4.00 | 2s | 100.85 | Estrada-Carmona et al. (2016) |
| Rb76 | Orthogneiss | 119.4 | 4.00 | 2s | 100.85 | Estrada-Carmona et al. (2016) |
| Rb76 | Orthogneiss | 118.8 | 4.00 | 2s | 100.85 | Estrada-Carmona et al. (2016) |
| Rb76 | Orthogneiss | 127.6 | 4.00 | 2s | 100.85 | Estrada-Carmona et al. (2016) |
| Rb76 | Orthogneiss | 119.8 | 4.00 | 2s | 100.85 | Estrada-Carmona et al. (2016) |
| Rb76 | Orthogneiss | 134.0 | 4.00 | 2s | 100.85 | Estrada-Carmona et al. (2016) |
| Rb76 | Orthogneiss | 130.2 | 4.00 | 2s | 100.85 | Estrada-Carmona et al. (2016) |
| Rb76 | Orthogneiss | 119.8 | 4.00 | 2s | 100.85 | Estrada-Carmona et al. (2016) |
| Rb76 | Orthogneiss | 129.4 | 4.00 | 2s | 100.85 | Estrada-Carmona et al. (2016) |
| Rb76 | Orthogneiss | 121.2 | 4.00 | 2s | 100.85 | Estrada-Carmona et al. (2016) |
| Rb76 | Orthogneiss | 135.8 | 4.00 | 2s | 100.85 | Estrada-Carmona et al. (2016) |
| Rb76 | Orthogneiss | 130.6 | 4.00 | 2s | 100.85 | Estrada-Carmona et al. (2016) |
| Rb76 | Orthogneiss | 130.4 | 4.00 | 2s | 100.85 | Estrada-Carmona et al. (2016) |
| Rb76 | Orthogneiss | 133.7 | 4.00 | 2s | 100.85 | Estrada-Carmona et al. (2016) |
| Rb76 | Orthogneiss | 132.8 | 4.00 | 2s | 100.85 | Estrada-Carmona et al. (2016) |
| Rb76 | Orthogneiss | 61.5 | 3.00 | 2s | 100.85 | Estrada-Carmona et al. (2016) |
| Rb76 | Orthogneiss | 132.7 | 4.00 | 2s | 100.85 | Estrada-Carmona et al. (2016) |
| Rb76 | Orthogneiss | 120.1 | 4.00 | 2s | 100.85 | Estrada-Carmona et al. (2016) |
| Rb76 | Orthogneiss | 136.7 | 4.00 | 2s | 100.85 | Estrada-Carmona et al. (2016) |
| Rb76 | Orthogneiss | 117.8 | 4.00 | 2s | 100.85 | Estrada-Carmona et al. (2016) |
| Rb76 | Orthogneiss | 133.7 | 4.00 | 2s | 100.85 | Estrada-Carmona et al. (2016) |
| Rb76 | Orthogneiss | 128.2 | 4.00 | 2s | 100.85 | Estrada-Carmona et al. (2016) |
| Rb76 | Orthogneiss | 134.4 | 4.00 | 2s | 100.85 | Estrada-Carmona et al. (2016) |
| Rb76 | Orthogneiss | 119.2 | 4.00 | 2s | 100.85 | Estrada-Carmona et al. (2016) |
| Rb76 | Orthogneiss | 117.3 | 4.00 | 2s | 100.85 | Estrada-Carmona et al. (2016) |
| Rb76 | Orthogneiss | 132.3 | 4.00 | 2s | 100.85 | Estrada-Carmona et al. (2016) |
| Rb76 | Orthogneiss | 115.2 | 4.00 | 2s | 100.85 | Estrada-Carmona et al. (2016) |
| Rb76 | Orthogneiss | 130.7 | 4.00 | 2s | 100.85 | Estrada-Carmona et al. (2016) |
| Rb76 | Orthogneiss | 133.0 | 4.00 | 2s | 100.85 | Estrada-Carmona et al. (2016) |
| Rb76 | Orthogneiss | 132.8 | 4.00 | 2s | 100.85 | Estrada-Carmona et al. (2016) |
| Rb76 | Orthogneiss | 130.3 | 4.00 | 2s | 100.85 | Estrada-Carmona et al. (2016) |
| Rb76 | Orthogneiss | 131.9 | 4.00 | 2s | 100.85 | Estrada-Carmona et al. (2016) |
| Rb76 | Orthogneiss | 128.8 | 4.00 | 2s | 100.85 | Estrada-Carmona et al. (2016) |
| Rb76 | Orthogneiss | 125.4 | 4.00 | 2s | 100.85 | Estrada-Carmona et al. (2016) |
| Rb76 | Orthogneiss | 136.0 | 4.00 | 2s | 100.85 | Estrada-Carmona et al. (2016) |
| Rb76 | Orthogneiss | 120.4 | 4.00 | 2s | 100.85 | Estrada-Carmona et al. (2016) |
| Rb76 | Orthogneiss | 132.1 | 4.00 | 2s | 100.85 | Estrada-Carmona et al. (2016) |
| Rb76 | Orthogneiss | 56.0 | 2.00 | 2s | 100.85 | Estrada-Carmona et al. (2016) |
| Rb76 | Orthogneiss | 135.2 | 4.00 | 2s | 100.85 | Estrada-Carmona et al. (2016) |
| Rb76 | Orthogneiss | 129.7 | 4.00 | 2s | 100.85 | Estrada-Carmona et al. (2016) |
| Rb76 | Orthogneiss | 130.1 | 4.00 | 2s | 100.85 | Estrada-Carmona et al. (2016) |
| Rb76 | Orthogneiss | 134.1 | 4.00 | 2s | 100.85 | Estrada-Carmona et al. (2016) |
| Rb76 | Orthogneiss | 129.6 | 4.00 | 2s | 100.85 | Estrada-Carmona et al. (2016) |
| Rb76 | Orthogneiss | 128.9 | 4.00 | 2s | 100.85 | Estrada-Carmona et al. (2016) |
| Rb76 | Orthogneiss | 127.5 | 4.00 | 2s | 100.85 | Estrada-Carmona et al. (2016) |
| Rb76 | Orthogneiss | 119.8 | 4.00 | 2s | 100.85 | Estrada-Carmona et al. (2016) |
| Rb76 | Orthogneiss | 134.5 | 4.00 | 2s | 100.85 | Estrada-Carmona et al. (2016) |
| Rb76 | Orthogneiss | 135.7 | 4.00 | 2s | 100.85 | Estrada-Carmona et al. (2016) |
| Rb76 | Orthogneiss | 130.8 | 4.00 | 2s | 100.85 | Estrada-Carmona et al. (2016) |
| Rb76 | Orthogneiss | 121.0 | 4.00 | 2s | 100.85 | Estrada-Carmona et al. (2016) |
| Rb105 | Leucosome | 112.0 | 4.00 | 2s | 72.16 | Estrada-Carmona et al. (2016) |
| Rb105 | Leucosome | 130.6 | 3.00 | 2s | 72.16 | Estrada-Carmona et al. (2016) |
| Rb105 | Leucosome | 124.7 | 3.00 | 2s | 72.16 | Estrada-Carmona et al. (2016) |
| Rb105 | Leucosome | 59.7 | 1.00 | 2s | 72.16 | Estrada-Carmona et al. (2016) |
| Rb105 | Leucosome | 131.4 | 3.00 | 2s | 72.16 | Estrada-Carmona et al. (2016) |
| Rb105 | Leucosome | 130.3 | 3.00 | 2s | 72.16 | Estrada-Carmona et al. (2016) |
| Rb105 | Leucosome | 140.9 | 3.00 | 2s | 72.16 | Estrada-Carmona et al. (2016) |
| Rb105 | Leucosome | 117.6 | 6.00 | 2s | 72.16 | Estrada-Carmona et al. (2016) |
| Rb105 | Leucosome | 128.9 | 3.00 | 2s | 72.16 | Estrada-Carmona et al. (2016) |
| Rb105 | Leucosome | 137.1 | 3.00 | 2s | 72.16 | Estrada-Carmona et al. (2016) |
| Rb105 | Leucosome | 128.3 | 3.00 | 2s | 72.16 | Estrada-Carmona et al. (2016) |
| Rb105 | Leucosome | 64.2 | 3.00 | 2s | 72.16 | Estrada-Carmona et al. (2016) |
| Rb105 | Leucosome | 144.6 | 3.00 | 2s | 72.16 | Estrada-Carmona et al. (2016) |
| Rb105 | Leucosome | 110.2 | 2.00 | 2s | 72.16 | Estrada-Carmona et al. (2016) |
| Rb105 | Leucosome | 61.6 | 1.00 | 2s | 72.16 | Estrada-Carmona et al. (2016) |
| Rb105 | Leucosome | 130.9 | 3.00 | 2s | 72.16 | Estrada-Carmona et al. (2016) |
| Rb105 | Leucosome | 117.8 | 3.00 | 2s | 72.16 | Estrada-Carmona et al. (2016) |
| Rb105 | Leucosome | 126.6 | 3.00 | 2s | 72.16 | Estrada-Carmona et al. (2016) |
| Rb105 | Leucosome | 123.7 | 3.00 | 2s | 72.16 | Estrada-Carmona et al. (2016) |
| Rb105 | Leucosome | 76.8 | 2.00 | 2s | 72.16 | Estrada-Carmona et al. (2016) |
| Rb105 | Leucosome | 95.1 | 2.00 | 2s | 72.16 | Estrada-Carmona et al. (2016) |
| Rb105 | Leucosome | 114.9 | 2.00 | 2s | 72.16 | Estrada-Carmona et al. (2016) |
| Rb105 | Leucosome | 101.1 | 3.00 | 2s | 72.16 | Estrada-Carmona et al. (2016) |
| Rb105 | Leucosome | 140.0 | 4.00 | 2s | 72.16 | Estrada-Carmona et al. (2016) |
| Rb105 | Leucosome | 131.2 | 3.00 | 2s | 72.16 | Estrada-Carmona et al. (2016) |
| Rb105 | Leucosome | 140.2 | 4.00 | 2s | 72.16 | Estrada-Carmona et al. (2016) |
| Rb105 | Leucosome | 139.6 | 3.00 | 2s | 72.16 | Estrada-Carmona et al. (2016) |
| Rb105 | Leucosome | 136.1 | 3.00 | 2s | 72.16 | Estrada-Carmona et al. (2016) |
| Rb105 | Leucosome | 130.0 | 3.00 | 2s | 72.16 | Estrada-Carmona et al. (2016) |
| Rb105 | Leucosome | 136.1 | 3.00 | 2s | 72.16 | Estrada-Carmona et al. (2016) |
| Rb105 | Leucosome | 121.0 | 3.00 | 2s | 72.16 | Estrada-Carmona et al. (2016) |
| Rb105 | Leucosome | 111.7 | 2.00 | 2s | 72.16 | Estrada-Carmona et al. (2016) |
| Rb105 | Leucosome | 116.6 | 3.00 | 2s | 72.16 | Estrada-Carmona et al. (2016) |
| Rb105 | Leucosome | 116.2 | 3.00 | 2s | 72.16 | Estrada-Carmona et al. (2016) |
| Rb105 | Leucosome | 130.0 | 3.00 | 2s | 72.16 | Estrada-Carmona et al. (2016) |
| Rb105 | Leucosome | 100.4 | 5.00 | 2s | 72.16 | Estrada-Carmona et al. (2016) |
| Rb105 | Leucosome | 129.2 | 3.00 | 2s | 72.16 | Estrada-Carmona et al. (2016) |
| Rb105 | Leucosome | 111.2 | 3.00 | 2s | 72.16 | Estrada-Carmona et al. (2016) |
| Rb105 | Leucosome | 115.6 | 3.00 | 2s | 72.16 | Estrada-Carmona et al. (2016) |
| Rb105 | Leucosome | 106.1 | 3.00 | 2s | 72.16 | Estrada-Carmona et al. (2016) |
| Rb105 | Leucosome | 58.6 | 1.00 | 2s | 72.16 | Estrada-Carmona et al. (2016) |
| Rb105 | Leucosome | 76.8 | 2.00 | 2s | 72.16 | Estrada-Carmona et al. (2016) |
| Rb105 | Leucosome | 109.4 | 2.00 | 2s | 72.16 | Estrada-Carmona et al. (2016) |
| Rb105 | Leucosome | 114.6 | 2.00 | 2s | 72.16 | Estrada-Carmona et al. (2016) |
| Rb105 | Leucosome | 111.5 | 3.00 | 2s | 72.16 | Estrada-Carmona et al. (2016) |
| Rb105 | Leucosome | 126.4 | 3.00 | 2s | 72.16 | Estrada-Carmona et al. (2016) |
| Rb105 | Leucosome | 130.6 | 3.00 | 2s | 72.16 | Estrada-Carmona et al. (2016) |
| Rb105 | Leucosome | 122.1 | 3.00 | 2s | 72.16 | Estrada-Carmona et al. (2016) |
| Rb105 | Leucosome | 129.8 | 3.00 | 2s | 72.16 | Estrada-Carmona et al. (2016) |
| Rb105 | Leucosome | 59.3 | 2.00 | 2s | 72.16 | Estrada-Carmona et al. (2016) |
| Rb105 | Leucosome | 131.4 | 3.00 | 2s | 72.16 | Estrada-Carmona et al. (2016) |
| Rb105 | Leucosome | 136.6 | 3.00 | 2s | 72.16 | Estrada-Carmona et al. (2016) |
| Rb105 | Leucosome | 102.3 | 3.00 | 2s | 72.16 | Estrada-Carmona et al. (2016) |
| Rb105 | Leucosome | 69.8 | 4.00 | 2s | 72.16 | Estrada-Carmona et al. (2016) |
| Rb105 | Leucosome | 128.6 | 4.00 | 2s | 72.16 | Estrada-Carmona et al. (2016) |
| Rb105 | Leucosome | 122.7 | 4.00 | 2s | 72.16 | Estrada-Carmona et al. (2016) |
| Rb105 | Leucosome | 125.4 | 3.00 | 2s | 72.16 | Estrada-Carmona et al. (2016) |
| Rb105 | Leucosome | 118.5 | 2.00 | 2s | 72.16 | Estrada-Carmona et al. (2016) |
| 277 | Intrusive | 29.0 | 1.00 | 2s | 416.13 | Keppie et al. (2012) |
| 277 | Intrusive | 27.0 | 1.00 | 2s | 416.13 | Keppie et al. (2012) |
| 277 | Intrusive | 28.0 | 1.00 | 2s | 416.13 | Keppie et al. (2012) |
| 277 | Intrusive | 28.0 | 1.00 | 2s | 416.13 | Keppie et al. (2012) |
| 277 | Intrusive | 29.0 | 1.00 | 2s | 416.13 | Keppie et al. (2012) |
| 277 | Intrusive | 27.0 | 1.00 | 2s | 416.13 | Keppie et al. (2012) |
| 277 | Intrusive | 27.0 | 1.00 | 2s | 416.13 | Keppie et al. (2012) |
| 277 | Intrusive | 27.0 | 1.00 | 2s | 416.13 | Keppie et al. (2012) |
| 277 | Intrusive | 29.0 | 1.00 | 2s | 416.13 | Keppie et al. (2012) |
| 277 | Intrusive | 29.0 | 1.00 | 2s | 416.13 | Keppie et al. (2012) |
| 277 | Intrusive | 29.0 | 40.00 | 2s | 416.13 | Keppie et al. (2012) |
| 277 | Intrusive | 28.0 | 1.00 | 2s | 416.13 | Keppie et al. (2012) |
| 277 | Intrusive | 27.0 | 1.00 | 2s | 416.13 | Keppie et al. (2012) |
| 277 | Intrusive | 27.0 | 1.00 | 2s | 416.13 | Keppie et al. (2012) |
| 277 | Intrusive | 28.0 | 1.00 | 2s | 416.13 | Keppie et al. (2012) |
| 277 | Intrusive | 27.0 | 1.00 | 2s | 416.13 | Keppie et al. (2012) |
| 277 | Intrusive | 27.0 | 4.00 | 2s | 416.13 | Keppie et al. (2012) |
| 277 | Intrusive | 28.0 | 1.00 | 2s | 416.13 | Keppie et al. (2012) |
| 277 | Intrusive | 28.0 | 2.00 | 2s | 416.13 | Keppie et al. (2012) |
| 246 | Intrusive | 31.0 | 1.00 | 2s | 416.13 | Keppie et al. (2012) |
| 246 | Intrusive | 32.0 | 2.00 | 2s | 416.13 | Keppie et al. (2012) |
| 246 | Intrusive | 30.0 | 1.00 | 2s | 416.13 | Keppie et al. (2012) |
| 246 | Intrusive | 31.0 | 1.00 | 2s | 416.13 | Keppie et al. (2012) |
| 246 | Intrusive | 30.0 | 1.00 | 2s | 416.13 | Keppie et al. (2012) |
| 246 | Intrusive | 31.0 | 1.00 | 2s | 416.13 | Keppie et al. (2012) |
| 246 | Intrusive | 30.0 | 1.00 | 2s | 416.13 | Keppie et al. (2012) |
| 246 | Intrusive | 32.0 | 1.00 | 2s | 416.13 | Keppie et al. (2012) |
| 246 | Intrusive | 31.0 | 1.00 | 2s | 416.13 | Keppie et al. (2012) |
| 246 | Intrusive | 30.0 | 1.00 | 2s | 416.13 | Keppie et al. (2012) |
| 246 | Intrusive | 30.0 | 1.00 | 2s | 416.13 | Keppie et al. (2012) |
| 246 | Intrusive | 29.0 | 1.00 | 2s | 416.13 | Keppie et al. (2012) |
| 246 | Intrusive | 32.0 | 1.00 | 2s | 416.13 | Keppie et al. (2012) |
| 246 | Intrusive | 30.0 | 1.00 | 2s | 416.13 | Keppie et al. (2012) |
| 246 | Intrusive | 29.0 | 1.00 | 2s | 416.13 | Keppie et al. (2012) |
| 246 | Intrusive | 31.0 | 1.00 | 2s | 416.13 | Keppie et al. (2012) |
| 246 | Intrusive | 31.0 | 1.00 | 2s | 416.13 | Keppie et al. (2012) |
| 246 | Intrusive | 30.0 | 1.00 | 2s | 416.13 | Keppie et al. (2012) |
| 246 | Intrusive | 30.0 | 1.00 | 2s | 416.13 | Keppie et al. (2012) |
| 246 | Intrusive | 30.0 | 1.00 | 2s | 416.13 | Keppie et al. (2012) |
| 246 | Intrusive | 30.0 | 1.00 | 2s | 416.13 | Keppie et al. (2012) |
| 246 | Intrusive | 33.0 | 1.00 | 2s | 416.13 | Keppie et al. (2012) |
| 246 | Intrusive | 30.0 | 1.00 | 2s | 416.13 | Keppie et al. (2012) |
| 246 | Intrusive | 30.0 | 1.00 | 2s | 416.13 | Keppie et al. (2012) |
| 281 | Intrusive | 905.0 | 10.00 | 2s | 416.13 | Keppie et al. (2012) |
| 281 | Intrusive | 1035.0 | 7.00 | 2s | 416.13 | Keppie et al. (2012) |
| 281 | Intrusive | 1013.0 | 8.00 | 2s | 416.13 | Keppie et al. (2012) |
| 281 | Intrusive | 30.0 | 1.00 | 2s | 416.13 | Keppie et al. (2012) |
| 281 | Intrusive | 29.0 | 1.00 | 2s | 416.13 | Keppie et al. (2012) |
| 281 | Intrusive | 27.0 | 1.00 | 2s | 416.13 | Keppie et al. (2012) |
| 281 | Intrusive | 30.0 | 1.00 | 2s | 416.13 | Keppie et al. (2012) |
| 281 | Intrusive | 29.0 | 3.00 | 2s | 416.13 | Keppie et al. (2012) |
| 281 | Intrusive | 890.0 | 16.00 | 2s | 416.13 | Keppie et al. (2012) |
| 281 | Intrusive | 871.0 | 94.00 | 2s | 416.13 | Keppie et al. (2012) |
| 281 | Intrusive | 1107.0 | 37.00 | 2s | 416.13 | Keppie et al. (2012) |
| 281 | Intrusive | 175.0 | 6.00 | 2s | 416.13 | Keppie et al. (2012) |
| 312 | Intrusive | 26.0 | 1.00 | 2s | 455.38 | Keppie et al. (2012) |
| 312 | Intrusive | 25.0 | 1.00 | 2s | 455.38 | Keppie et al. (2012) |
| 312 | Intrusive | 26.0 | 1.00 | 2s | 455.38 | Keppie et al. (2012) |
| 312 | Intrusive | 25.0 | 1.00 | 2s | 455.38 | Keppie et al. (2012) |
| 312 | Intrusive | 31.0 | 1.00 | 2s | 455.38 | Keppie et al. (2012) |
| 312 | Intrusive | 596.0 | 6.00 | 2s | 455.38 | Keppie et al. (2012) |
| 312 | Intrusive | 941.0 | 10.00 | 2s | 455.38 | Keppie et al. (2012) |
| 312 | Intrusive | 25.0 | 1.00 | 2s | 455.38 | Keppie et al. (2012) |
| 312 | Intrusive | 1050.0 | 9.00 | 2s | 455.38 | Keppie et al. (2012) |
| 312 | Intrusive | 26.0 | 1.00 | 2s | 455.38 | Keppie et al. (2012) |
| 312 | Intrusive | 28.0 | 1.00 | 2s | 455.38 | Keppie et al. (2012) |
| 312 | Intrusive | 26.0 | 1.00 | 2s | 455.38 | Keppie et al. (2012) |
| 312 | Intrusive | 25.0 | 1.00 | 2s | 455.38 | Keppie et al. (2012) |
| 312 | Intrusive | 24.0 | 1.00 | 2s | 455.38 | Keppie et al. (2012) |
| 312 | Intrusive | 26.0 | 1.00 | 2s | 455.38 | Keppie et al. (2012) |
| 312 | Intrusive | 32.0 | 1.00 | 2s | 455.38 | Keppie et al. (2012) |
| 312 | Intrusive | 24.0 | 1.00 | 2s | 455.38 | Keppie et al. (2012) |
| 312 | Intrusive | 88.0 | 8.00 | 2s | 455.38 | Keppie et al. (2012) |
| 312 | Intrusive | 26.0 | 1.00 | 2s | 455.38 | Keppie et al. (2012) |
| 312 | Intrusive | 124.0 | 1.00 | 2s | 455.38 | Keppie et al. (2012) |
| 312 | Intrusive | 28.0 | 5.00 | 2s | 455.38 | Keppie et al. (2012) |
| 312 | Intrusive | 26.0 | 1.00 | 2s | 455.38 | Keppie et al. (2012) |
| 312 | Intrusive | 25.0 | 1.00 | 2s | 455.38 | Keppie et al. (2012) |
| 312 | Intrusive | 25.0 | 1.00 | 2s | 455.38 | Keppie et al. (2012) |

**References**

Ducea, M.N., Gehrels, G.E., Shoemaker, S., Ruiz, J., Valencia, V.A. (2004). Geologic evolution of the Xolapa Complex, southern Mexico: Evidence from U-Pb zircon geochronology. Geological Society of America Bulletin, v. 116, p. 1016-1025.

Estrada-Carmona, J., Solari, L.A., Ortega-Obregón, C. (2016). Petrochronology of the migmatization event of the Xolapa Complex, Mexico, microchemistry and equilibrium growth of zircon and garnet. International Geology Review, v. 58, p. 1382-1397.

Hernández-Pineda, G. A., Solari, L. A., Gómez-Tuena, A., Méndez-Cárdenas, D.L., Pérez-Arvizu, O. (2011). Petrogenesis and thermobarometry of the∼ 50 Ma rapakivi granite-syenite Acapulco intrusive: Implications for post-Laramide magmatism in southern Mexico. Geosphere, v. 7, p. 1419-1438.

Keppie, D.F., Hynes, A.J., Lee, J.K., Norman, M. (2012). Oligocene‐Miocene back‐thrusting in southern Mexico linked to the rapid subduction erosion of a large forearc block. Tectonics, v. 31.

Latorre, C.I., 2018. Analisis de fabricas de las migmatitas del Oligoceno del terreno Xolapa, localizadas entre Puerto Escondido y San Gabriel Mixtepec, Oaxaca, Mexico. [M. Sc. thesis]. Juriquilla, Queretaro, Universidad Nacional Autonoma de Mexico, Centro de Geociencias. 67 p.

Peña-Alonso, T.A., Estrada-Carmona, J., Molina-Garza, R.S., Solari, L., Levresse, G., Latorre, C. (2017). Lateral spreading of the middle to lower crust inferred from Paleocene migmatites in the Xolapa Complex (Puerto Escondido, Mexico): Gravitational collapse of a Laramide orogen?. Tectonophysics, v. 706, p. 143-163.

Pérez-Gutiérrez, R., Solari, L.A., Tuena, A.G., Martens, U.C., 2009. Mesozoic geologic evolution of the Xolapa migmatitic complex north of Acapulco, southern Mexico: Implications for paleogeographic reconstructions. Revista Mexicana de Ciencias Geológicas, v. 26, p. 201-221.

Talavera-Mendoza, O., Ruiz, J., Corona-Chavez, P., Gehrels, G.E., Sarmiento-Villagrana, A., García-Díaz, J.L., Salgado-Souto, S.A. (2013). Origin and provenance of basement metasedimentary rocks from the Xolapa Complex: New constraints on the Chortis–southern Mexico connection. Earth and Planetary Science Letters, v. 369, p. 188-199.

Valencia, V.A., Ducea, M.N., Mendoza, O.T., Gehrels, G., Ruiz, J., Shoemaker, S. (2009). U-Pb geochronology of granitoids in the north-western boundary of the Xolapa Terrane. Revista Mexicana de Ciencias Geológicas, v. 26, p. 189-200.
